# Development of high-voltage bipolar redox-active organic molecules through the electronic coupling of catholyte and anolyte structures†

**DOI:** 10.1039/d2sc03450f

**Published:** 2022-09-01

**Authors:** Jacob S. Tracy, Elena S. Horst, Vladislav A. Roytman, F. Dean Toste

**Affiliations:** Chemical Science Division, Lawrence Berkeley National Laboratory, University of California Berkeley CA 94720-1460 USA fdtoste@berkeley.edu; Department of Chemistry, University of California Berkeley CA 94720-1460 USA; Joint Center for Energy Storage Research (JCESR) 9700 South Cass Avenue Argonne Illinois 60439 USA

## Abstract

All-organic non-aqueous redox flow batteries (O-NRFBs) are a promising technology for grid-scale energy storage. However, most examples of high-voltage (>2 V) O-NRFBs rely upon the use of distinct anolytes and catholytes separated by a membrane or porous separator which can result in crossover of redox active material from one side of the battery to the other. The resulting electrolyte mixing leads to irreversible reductions in energy density and capacity. A potentially attractive solution to overcome this crossover issue is the implementation of symmetric flow batteries where a single bipolar molecule functions as both an anolyte and a catholyte. Herein, we report the development of a new class of bipolar redox active materials for use in such symmetric flow batteries through the electronic coupling of phenothiazine catholytes and phthalimide anolytes. Such a strategy results in hybrid molecules possessing higher cell voltages than what could be obtained together by their uncoupled building blocks. Performance in flow batteries is demonstrated for two members of this new class of molecules, with the highest performing candidate featuring a Δ*E* of 2.31 V and demonstrating 93.6% average coulombic efficiency, 86.8% energy efficiency, and 68.6% capacity retention over the course of 275 charge–discharge cycles and 5 cell polarity reversals. Finally, the superior performance of symmetric O-NRFBs is experimentally confirmed by comparing these results to an asymmetric flow battery constructed with a distinct phenothiazine catholyte and a distinct phthalimide anolyte on opposing sides of the cell.

## Abstract 2

A new class of bipolar redox active molecules with enhanced voltages is reported *via* the electronic coupling of phthalimide anolytes and phenothiazine catholytes. Their performance is tested under relevant nonaqueous redox flow battery conditions.

## Graphical abstract

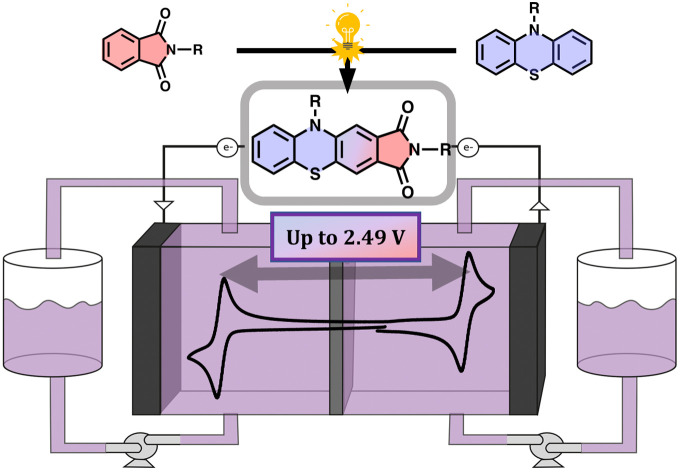

## Introduction

As the world moves away from carbonized fuel sources in an attempt to reduce greenhouse gas emissions, energy generation from solar and wind-based sources is expected to dramatically increase.^1^ However, significant reliance upon such intermittent energy sources can result in a destabilization of the energy grid unless sufficient grid-scale energy storage is available to balance generation and demand.^2^ Redox flow batteries (RFBs) represent a promising electrical energy storage technology to serve this balancing role, as the technology's decoupling of battery power and total capacity allows for greater levels of engineering flexibility in their incorporation within the power grid.^3^ Already, aqueous redox flow batteries (ARFB) based upon vanadium redox couples are being commercialized and actively employed in grid management.^4^ Despite the appeal of these systems, they tend to suffer from relatively low energy densities in part due to the narrow thermodynamic electrochemical window of water (<1.5 V).

Non-aqueous redox flow batteries (NRFB) offer the opportunity for higher energy densities owing to electrochemical windows that can exceed 5 V.^5^ Additionally, the use of organic solvents can be coupled with redox active organic materials (ROMs) in all-organic non-aqueous redox flow batteries (O-NRFBs) to allow for high levels of control over electronic and physical properties through precise molecular engineering.^6^ However, the use of small molecules in O-NRFBs to achieve high voltage batteries most often requires an “asymmetric” battery setup that uses different ROMs on the anolyte and catholyte side of the battery.^7^ As a result, entropically-driven crossover of ROMs from one side of the battery to the other results in lower coulombic efficiency,^8^ a permanent reduction in battery capacity and energy density,^9^ and presents new paths for chemical degradation caused by electrolyte mixing.^10^

A variety of strategies, each with their own disadvantages, have been employed to counteract the effects of crossover. Ion-exchange membranes, extensively used in the context of aqueous redox flow batteries,^11^ are currently limited in O-NRFBs by chemical stability concerns, low ionic conductivity, and high rates of swelling.^12^ As a result, O-NRFBs often resort to utilizing mesoporous battery separators whose pore sizes are too large to offer sufficiently high levels of selectivity for transport of the supporting electrolyte over the small molecule catholyte and anolyte species.^9^ The use of large redox active polymers and oligomers in conjunction with size exclusion membranes has shown promising initial results to overcome this lack of selectivity, but significant synthetic challenges remain to further refine these systems to achieve high energy density and reasonable transport properties.^13–15^ Finally, simply mixing the small molecule anolyte and catholyte materials on both sides of the battery, potentially in the form of eutectic mixtures, has also been used to reduce crossover. However, this strategy can lead to high viscosities,^16^ theoretically reduced energy density from lower solubilities as a result of more species on each side of the separator requiring solvation, chemical compatibility issues,^10^ and the build-up of large concentration gradients between cells during cycling that require electrolyte rebalancing.^9^

One technologically simple strategy to avoid the permanent loss of battery capacity resulting from crossover is the use of bipolar redox-active molecules (BRMs).^17^ In such a “symmetric” battery construction, a ROM capable of undergoing reversible oxidation and reduction is utilized as the active electrolyte on both the catholyte and anolyte side of the battery. As a result, net crossover theoretically results in no changes to energy density and the impacts of crossover become limited to decreases in coulombic efficiency. Additionally, crossover rates themselves become minimized with BRMs through a reduction of chemical gradients across the battery, which approach zero in the fully discharged state.

While significant progress has been accomplished discovering ROMs that result in large cell potentials, the discovery of new BRMs not reliant upon the simple stitching of two distinct redox active molecules together *via* an electronically insulating tether has seen markedly less activity.^18^ This is especially true with regards to BRMs capable of producing cell voltages >2 V.^19^ Our strategy to develop new high voltage BRMs sought to capitalize on the extensive body of work surrounding the development of asymmetric O-NRFBs by electronically coupling known catholyte and anolyte structures into a single molecule. By electronically coupling the two materials in close structural proximity, it was envisioned that the opposing electronic natures of the catholyte scaffold – which tends to be electron rich – and the anolyte scaffold – which tends to be electron-deficient – would have a synergistic effect on the resulting electrochemical potentials of the merged molecule and result in increased battery voltages when compared to what could be achieved in analogous asymmetric or tethered BRM battery systems.

Specifically, the electron-rich nature of the catholyte scaffold was envisioned to raise the energy of the resulting merged LUMO while the electron-deficient nature of the anolyte scaffold would lower the energy of the merged HOMO, resulting in an overall increase in cell potential. Additionally, if electronic coupling is done through the fusion of commonly shared motifs – *e.g.*, coupling aromatic rings from a catholyte and anolyte in a way in which one of these aromatic rings is made redundant – lower molecular weight BRMs with potentially higher theoretical energy densities would be possible as compared to analogous systems that link catholytes and anolytes together through insulating tethers which add molecular weight. Such an overall strategy is supported by the theoretic work of Fornari, de Silva, and co-workers in aqueous RFBs^20^ and was recently, and independent of our efforts, demonstrated within O-NRFBs by the combined groups of Zhang, Xu, and Zhao.^19*g*^

**Fig. 1:**
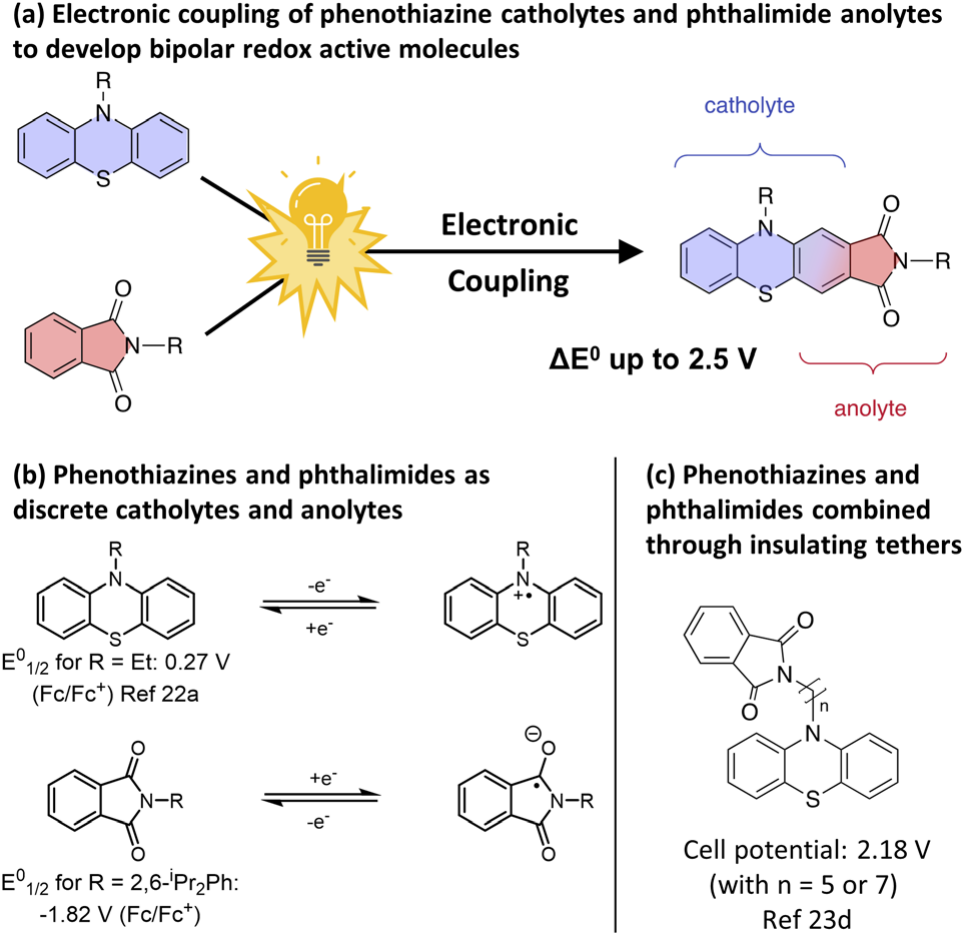
(a) Formation of bipolar redox active molecules through the electronic coupling of phenothiazine catholytes and phthalimide anolytes. (b) Phenothiazines and phthalimides as catholytes and anolytes. (c) Formation of bipolar redox active molecules through the combination of a phenothiazine and a phthalimide *via* an insulating tether.

To test our strategy, we proposed the merger of the phenothiazine class of catholyte with the phthalimide class of anolyte. The favourable voltages of their redox couples and their high levels of electrochemical reversibility have resulted in these scaffolds being independently employed numerous times in asymmetric O-NRFB systems (Fig. 1b)^7*a*,21,22^ and in the context of BRMs through the use of electronically insulating linkers (Fig. 1c).^23^ However, the electronic merger of these two systems had not been explored and represented an excellent test case for our proposed strategy.

**Fig. 2:**
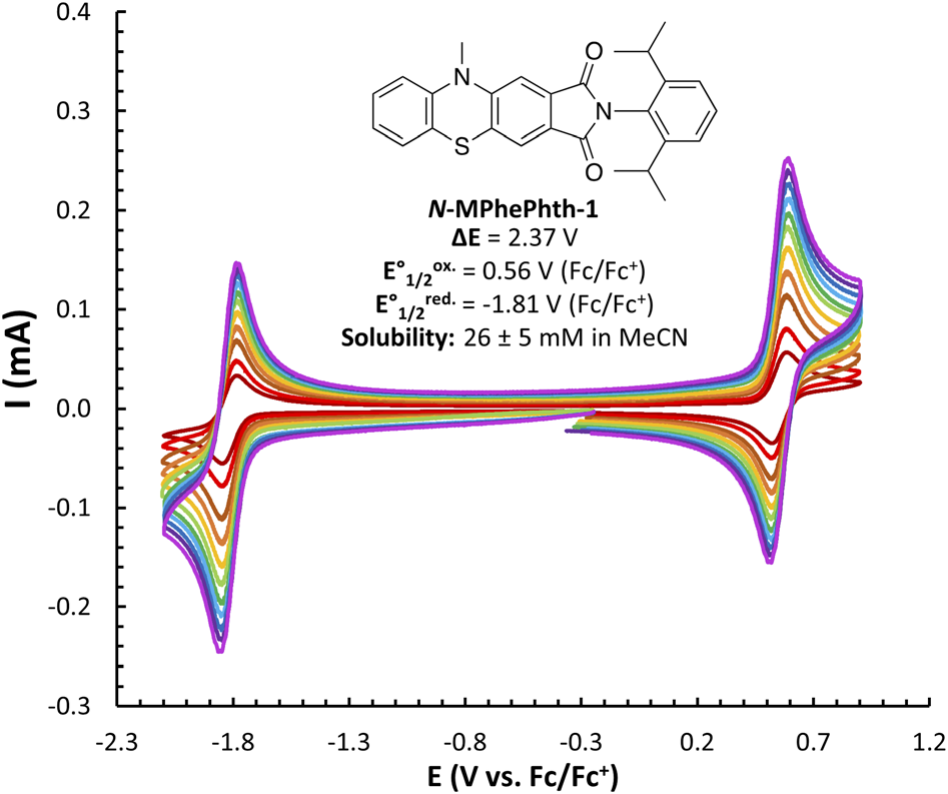
CV studies of *N*-MPhePhtha-1 (<5 mM in 0.5 M TBAPF_6_/MeCN) with scan rates varying from 50 to 1000 mV s^−1^. Solubility is an average of three measurements.

**Fig. 3:**
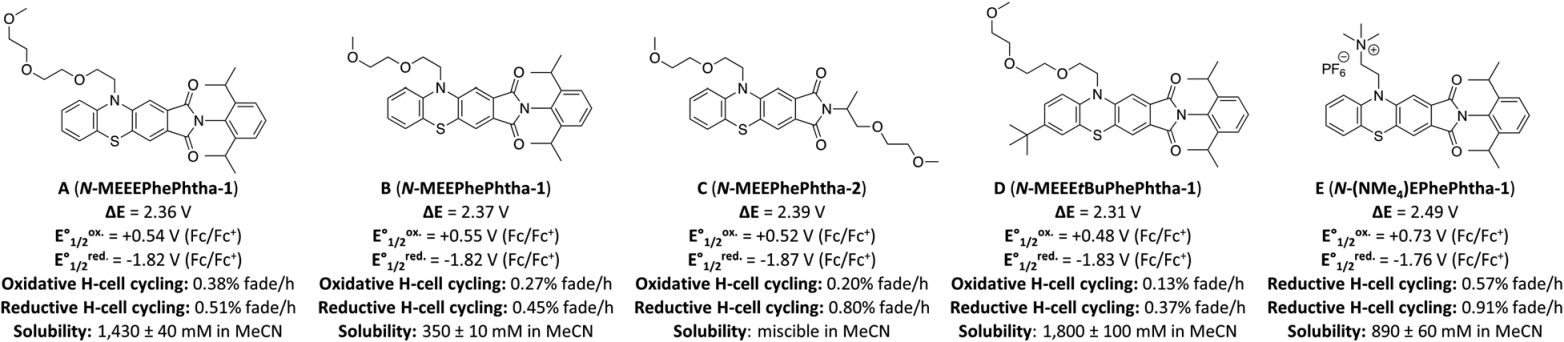
Library of phenothiazine/phthalimide hybrids (PhePhtha) along with measured potentials, rates of capacity fade in static H-cell cycling for oxidation and reduction, and solubility in acetonitrile. Static cycling was performed with 5 mM of active material in 0.5 M TBAPF_6_/MeCN for a total of 100 cycles. Rates of fade are given in % discharge capacity lost per hour, averaged over the 100 cycles, and normalized relative to the theoretical discharge capacity. Solubilities in acetonitrile without supporting electrolyte are the average of three measurements.

We began our studies by synthesizing a hybrid phenothiazine/phthalimide structure (*N*-MPhePhtha-1) bearing an *N*-methyl group on the phenothiazine portion and a 2,6-diisopropylphenyl moiety on the phthalimide. Data from cyclic voltammetry in 0.5 M TBAPF_6_/MeCN showed reversible oxidation (0.56 V *vs.* Fc/Fc^+^) and reduction (−1.81 V *vs.* Fc/Fc^+^) couples with a Δ*E*^0^ of 2.37 V across a range of scan rates (Fig. 2). This Δ*E*^0^ represents a 290 mV improvement in voltage as compared to batteries that can be made from analogous phenothiazine catholytes and phthalimide anolytes which are not electronically coupled (Fig. 1b). Unfortunately, the low solubility of *N*-MPhePhtha-1 in 0.5 M TBAPF_6_/MeCN (<5 mM) and in acetonitrile alone (26 ± 5 mM) prevented the study of longer-term cycling stability. In order to overcome solubility limitations, the imide and phenothiazine nitrogen atoms were used as easy points of synthetic modifications to introduce solubilizing groups such as poly ethers and ammonium salts. For each of these compounds, the voltage gap between oxidation and reduction was assessed through CV studies while electrochemical reversibility and stability were assessed *via* static galvanostatic charge–discharge cycling (100 cycles, 5 mM active material in 0.5 M TBAPF_6_/MeCN) in a 3-electrode H-cell equipped with a glass-frit separator (Fig. 3).

**Fig. 4:**
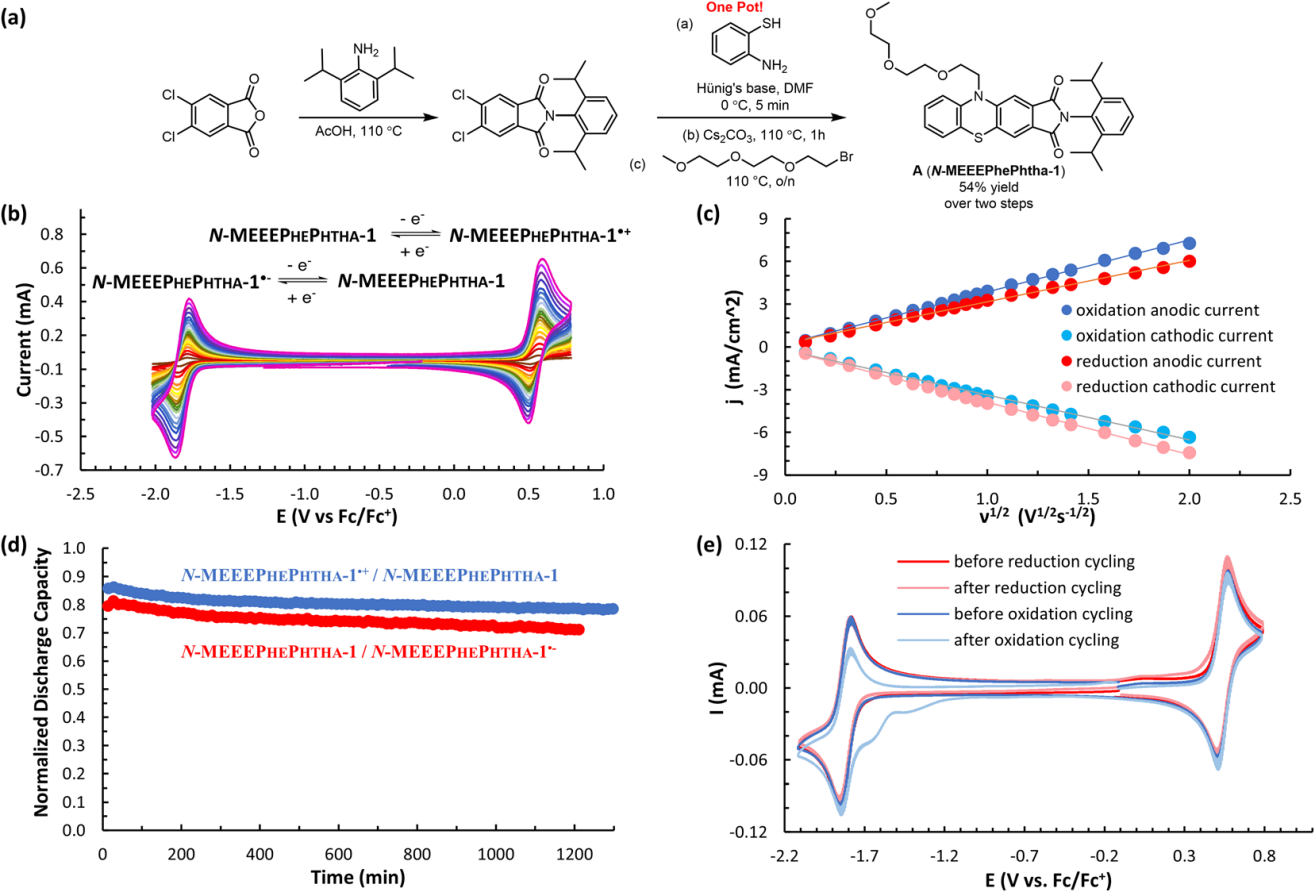
(a) Two-pot synthesis of A (*N*-MEEEPhePtha-1) from commercially available starting materials. (b) CV studies of A (*N*-MEEEPhePtha-1) (5 mM in 0.5 M TBAPF_6_/MeCN) with scan rates varying from 10 to 4000 mV s^−1^. (c) Plots of anodic and cathodic peak current densities (j) *vs.* the square root of the sweep rate (*ν*^1/2^) for oxidation (blue) and reduction (red) of A (*N*-MEEEPhePtha-1). (d) Plot of discharge capacity (normalized to theoretical capacity) *vs.* time (total of 100 cycles) for H-cell cycling of A (*N*-MEEEPhePtha-1) (5 mM in 0.5 M TBAPF_6_/MeCN) for both oxidation (blue) and reduction (red). The 100 cycles cover a time range of 21 hours for oxidation and 20 hours for reduction. (e) CVs (100 mV s^−1^) before and after H-cell cycling of A (*N*-MEEEPhePtha-1).

While all of these groups resulted in significantly improved solubility, A (*N*-MEEEPhePhtha-1), featuring an 2-(2-(2-methoxyethoxy)ethoxy)ethyl group on the phenothiazine nitrogen, proved especially attractive due to its high solubility (1430 ± 40 mM in MeCN), high stability towards reduction (0.51% normalized capacity loss per hour) and oxidation (0.38% normalized capacity loss per hour) (Fig. 3 and 4d), and its ease of synthetic accessibility *via* an operationally simple two-pot procedure from relatively inexpensive and commercially available starting materials (Fig. 4a). We further studied A (*N*-MEEEPhePhtha-1) through scan rate-dependent cyclic voltammetry and found oxidation and reduction to both be transport-limited redox processes (Fig. 4b and c) with an average diffusion coefficient of 6.3 × 10^−6^ cm^2^ s^−1^ as determined from the Randle–Ševčík equation and a heterogeneous electron-transfer rate of 8.3 × 10^−2^ cm s^−1^ for oxidation and 8.2 × 10^−2^ cm s^−1^ for reduction as determined by the Nicholson method.^24^ These data for the other compounds studied are available in the ESI.†

**Fig. 5:**
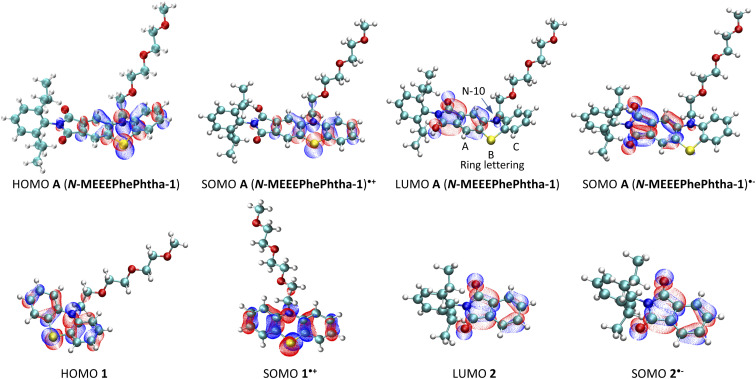
Frontier molecular orbitals for A (*N*-MEEEPhePtha-1), A (*N*-MEEEPhePtha-1)˙^−^, A (*N*-MEEEPhePtha-1)˙^+^, 1, 1˙^+^, 2, and 2˙^−^.

**Fig. 6:**
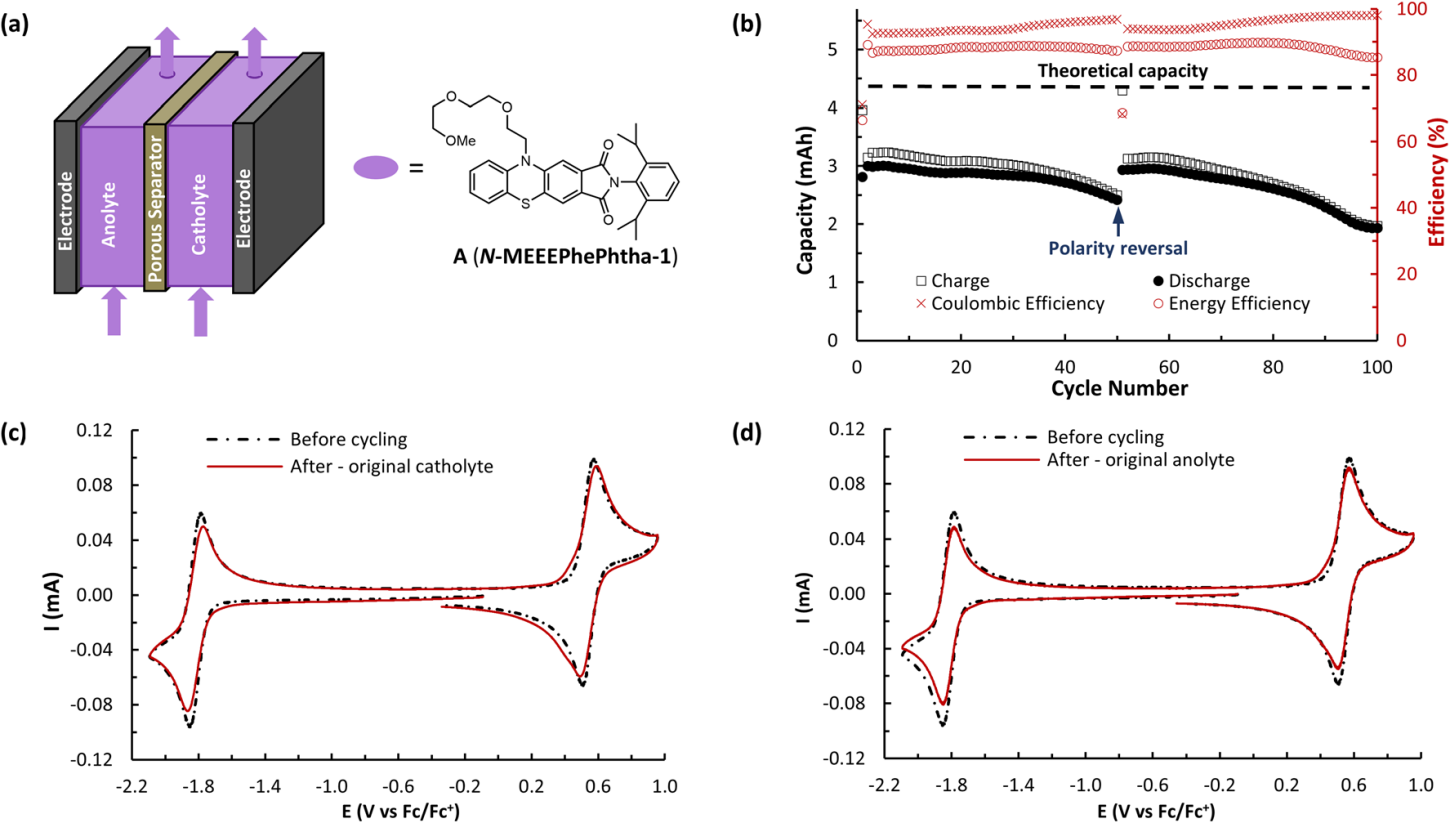
(a) Simplified flow cell schematic showing a symmetrical design featuring A (*N*-MEEEPhePtha-1) as the BRM. (b) Flow cycling data showing charge and discharge capacities and coulombic and energy efficiencies. Both cell reservoirs were charged with 6.5 mL of 25 mM of A (*N*-MEEEPhePtha-1) in 0.5 M TBAPF_6_/MeCN. Charging and discharging were performed at 10 mA cm^−2^ with voltage cut offs ±350 mV from the cell's Δ*E*^0^ of 2.36 V. The 100 cycles cover a time of approximately 22 hours. (c) CVs (100 mV s^−1^) before and after flow cell cycling of A (*N*-MEEEPhePtha-1). Material analysed was removed from the reservoir that served as the catholyte for cycles 1–50 and the anolyte for cycles 51–100. All solutions were diluted in a 1 : 4 ratio with 0.5 M TBAPF_6_/MeCN before data acquisition. (d) CVs (100 mV s^−1^) before and after flow cell cycling of A (*N*-MEEEPhePtha-1). Material analysed was removed from the reservoir that served as the anolyte for cycles 1–50 and the catholyte for cycles 51–100. All solutions were diluted in a 1 : 4 ratio with 0.5 M TBAPF_6_/MeCN before data acquisition.

**Fig. 7:**
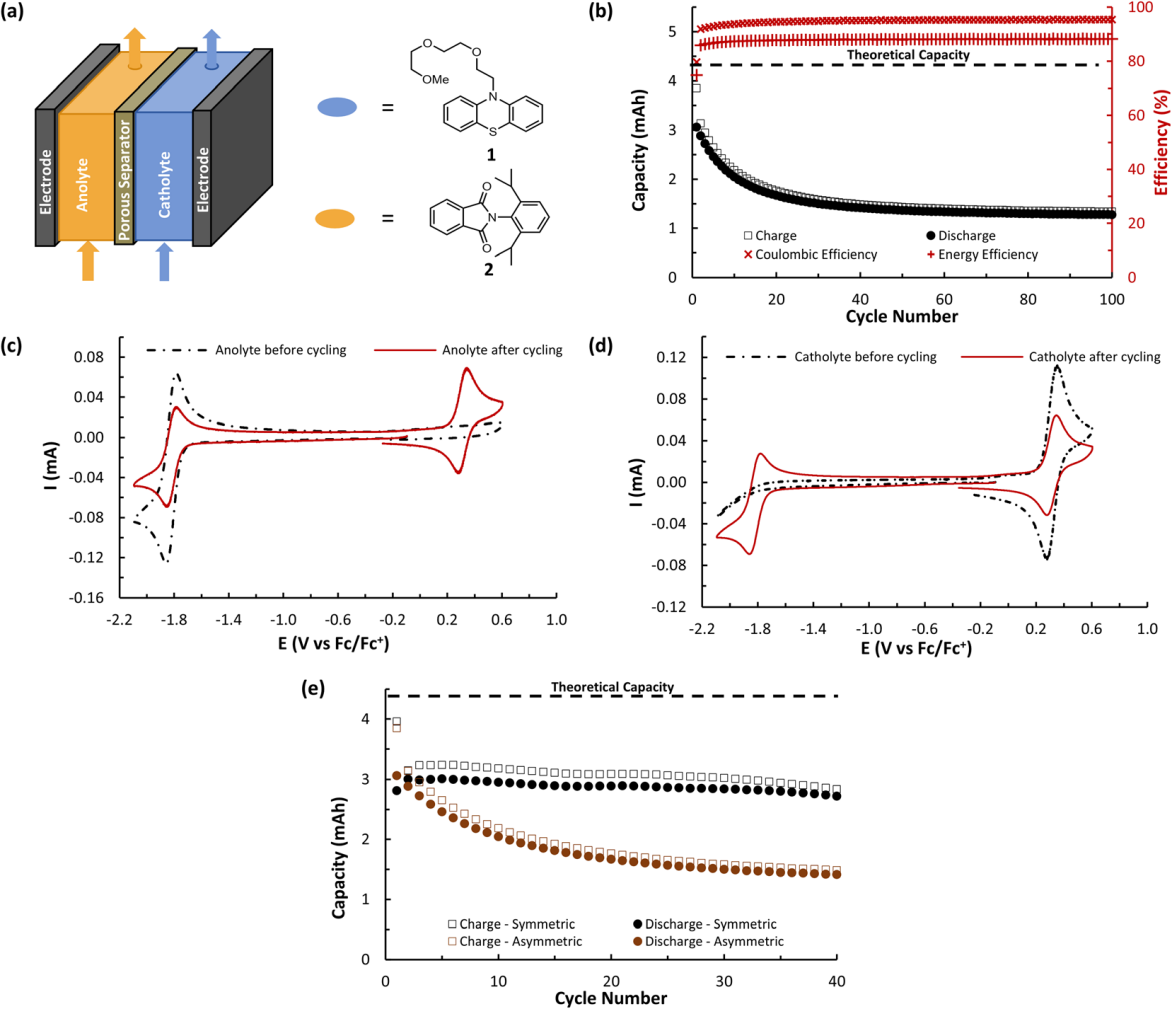
(a) Simplified schematic showing an asymmetric flow cell design featuring phenothiazine 1 as catholyte and phthalimide 2 as anolyte. (b) Flow cycling data showing charge and discharge capacities as well as coulombic and energy efficiencies. The catholyte cell reservoir was charged with 6.5 mL of 25 mM of phenothiazine 1 in 0.5 M TBAPF_6_/MeCN while the anolyte cell reservoir was charged with 6.5 mL of 25 mM of phthalimide 2 in 0.5 M TBAPF_6_/MeCN. Charging and discharging was performed at 10 mA cm^−2^ with voltage cut offs ±350 mV from the cell's theoretical Δ*E*^0^ of 2.13 V. The 100 cycles cover a time range of over 12 hours. (c) CVs (100 mV s^−1^) before and after flow cell cycling of the anolyte reservoir. All solutions were diluted in a 1 : 4 ratio with 0.5 M TBAPF_6_/MeCN before data acquisition. (d) CVs (100 mV s^−1^) before and after flow cell cycling of the catholyte reservoir. All solutions were diluted in a 1 : 4 ratio with 0.5 M TBAPF_6_/MeCN before data acquisition. (e) Overlay of flow cycling data showing charge and discharge capacities over the first 40 cycles for the symmetric flow battery featuring A (*N*-MEEEPhePtha-1) (black markers) and the asymmetric flow battery featuring phenothiazine 1 and phthalimide 2 (brown markers).

DFT calculations were then performed on A (*N*-MEEEPhePhtha-1), A (*N*-MEEEPhePhtha-1)˙^−^, and A (*N*-MEEEPhePhtha-1)˙^+^ to learn about their electronic structures and how they compare to analogously substituted phenothiazine 1 and phthalimide 2 lacking electronic coupling (Fig. 5). Qualitatively, the HOMO of A (*N*-MEEEPhePhtha-1) largely reflects that of phenothiazine 1, with minor additional contributions from the imide N. Likewise, the LUMO of A (*N*-MEEEPhePhtha-1) resembles that of phthalimide 2, with contributions to the orbital largely resulting from the imide and ring A with additional minor contributions from N-10 and ring C. Similar conclusions come from comparing SOMOs of the corresponding radical anions and cations. Quantitatively, our strategy of electronic coupling was calculated to have a beneficial impact of 340 mV on overall cell potential with 290 mV resulting from an increase in oxidation potential of A (*N*-MEEEPhePhtha-1) relative to phenothiazine 1 and just 50 mV resulting from a decrease in reduction potential of A (*N*-MEEEPhePhtha-1) relative to phthalimide 2 (see ESI† for details on calculations). This is in reasonable agreement with experimental CVs showing an improvement in theoretical cell potential of 220 mV, with all of this enhancement resulting from an increase in oxidation potential of A (*N*-MEEEPhePhtha-1) relative to phenothiazine 1 (*vide infra*, Fig. 6 and 7 for relevant CVs). These results, showing much of the beneficial improvement in cell potential of A (*N*-MEEEPhePhtha-1) resulting from an increase in its oxidation potential, may indicate that future efforts aimed at coupling phthalimide anolytes with more electron-donating catholyte fragments may result in even larger voltage enhancements by improving both the oxidation and reduction potentials of the hybrid molecules. Similar calculations on D (*N*-MEEE*t*BuPhePhtha-1) are available in the ESI.†

The ability of A (*N*-MEEEPhePhtha-1) to act as a BRM under flow conditions was next explored utilizing a flow battery design previously described by Brushett and coworkers (Fig. 6).^22*b*,25^ The battery was assembled utilizing a Daramic-175 porous separator and the catholyte and anolyte reservoirs were both filled with 6.5 mL of 25 mM A (*N*-MEEEPhePhtha-1) in 0.5 M TBAPF_6_/MeCN. Charging and discharging was performed at constant current densities of 10 mA cm^−2^ until reaching voltage cut offs ±350 mV from the cell's theoretical Δ*E*^0^ of 2.36 V. The utilization rate, based upon discharge capacity, reached a peak of 69.0% during cycle 5 and a capacity retention of 80% was observed after 50 cycles (Fig. 6b). At this point, the cell polarity was reversed in order to further highlight the bipolar nature of the BRM and an additional 50 charge–discharge cycles were performed. The reversal in polarity resulted in near complete recovery of lost capacity, reaching a utilization rate of 67.8% by cycle 56, which equates to 98.3% capacity retention. This ability to rebalance cells through a reversal in polarity and recover capacity loss due to chemical imbalances across the cell highlights an additional benefit of BRMs. At the end of 100 cycles, capacity retention was 64.3% relative to the peak discharge in cycle 5. Average coulombic efficiency was 94.6% while average energy efficiency was 85.3%. Post analysis CVs of the two reservoirs showed some evidence of decomposition by-products, as observed by shoulders on the oxidation redox couple and a reduction in peak currents as compared to that observed before flow cycling (Fig. 6c and d).

A (*N*-MEEEPhePhtha-1)'s hybrid phenothiazine-phthalimide nature also provided a unique opportunity to experimentally compare the performance of an O-NRFB featuring a BRM with that of an analogous battery setup in an asymmetric fashion featuring discrete anolyte (phthalimide) and catholyte (phenothiazine) molecules on opposing sides of the separator. To do so, an asymmetric battery was assembled utilizing phenothiazine 1 and phthalimide 2 with analogous *N*-substitution as A (*N*-MEEEPhePhtha-1) (Fig. 7a). These substitutions were conserved to ensure the results would allow for a direct comparison that better isolates the effect of symmetric BRM *vs.* asymmetric ROM flow batteries. By analogy to the symmetric flow battery, the catholyte reservoir was loaded with 6.5 mL of 25 mM phenothiazine 1 in 0.5 M TBAPF_6_/MeCN while the anolyte reservoir was loaded with 6.5 mL of 25 mM phthalimide 2 in 0.5 M TBAPF_6_/MeCN. Charging rates were 10 mA cm^−2^ with voltage cut offs ±350 mV from the cell's Δ*E*^0^ of 2.13 V. Peak utilization of 70.4% and overall coulombic (94.7%) and energy (87.8%) efficiencies were comparable to the performance of the symmetric flow battery featuring A (*N*-MEEEPhePhtha-1). However, a rapid decline in discharge capacity was observed following the first cycle in the asymmetric flow battery, with 50.2% capacity retention being reached after just 27 cycles. The rate of capacity decline then flattened until reaching a final 41.7% capacity retention after 100 cycles. Such a rapid decline in capacity that flattens with time is consistent with entropically-driven mixing of catholyte and anolyte materials until a homogenous mixture is obtained.^9^ This interpretation is supported by post-cycling CV analysis which shows a near uniform distribution of catholyte and anolyte across both battery reservoirs (Fig. 7c and d). These sets of experiments provide direct experimental evidence supporting the value of BRMs as a tool to reduce the impacts of crossover in O-NARFBs (Fig. 7e).

**Fig. 8:**
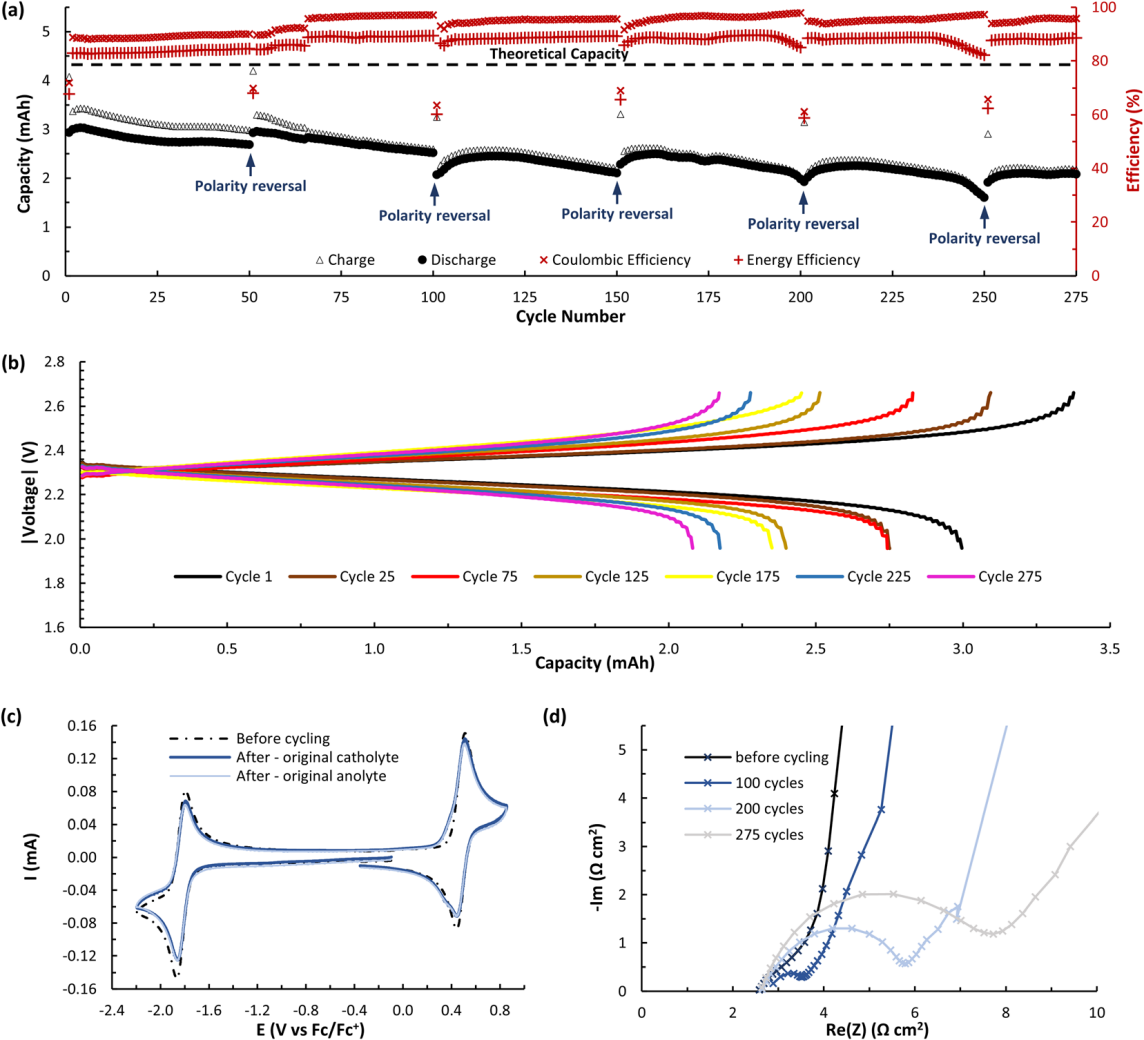
(a) Flow cycling data showing charge and discharge capacities as well as coulombic and energy efficiencies for a symmetric flow cell featuring D (*N*-MEEE*t*BuPhePhtha-1) as the BRM. Both flow cell reservoirs were charged with 6.5 mL of 25 mM of D (*N*-MEEE*t*BuPhePhtha-1) in 0.5 M TBAPF_6_/MeCN. Charging and discharging was performed at 10 mA cm^−2^ with voltage cut offs ±350 mV from the cell's theoretical Δ*E*^0^ of 2.31 V. The 275 cycles include polarity reversals every 50 cycles and cover a time range of approximately 57 hours. (b) Charge–discharge curves for cycles 1, 25, 75, 125, 175, 225, and 275. Absolute values for voltage are shown for ease of comparison as cycles 75, 175, and 275 were performed with reversed cell polarities. (c) CVs before and after flow cell cycling of D (*N*-MEEE*t*BuPhePhtha-1). All solutions were diluted in a 3 : 7 ratio with 0.5 M TBAPF_6_/MeCN before data acquisition. Original catholyte refers to the reservoir that served as catholyte for cycles 1–50 and original anolyte refers to the reservoir that served as the anolyte for cycles 1–50. (d) Electrochemical impedance spectroscopy (EIS) on the flow cell before cycling and after cycles 100, 200, and 275.

While A (*N*-MEEEPhePhtha-1) provided proof of principal that our PhePhtha molecules are capable of acting as BRMs in a flow battery environment, we sought to further improve long-term cycling stability. To do so, we turned our attention to D (*N*-MEEE*t*BuPhePhtha-1), which features a *t*-butyl group at the 7-position of the aromatic core. This structural modification was not only associated with higher cycling stability in H-cell galvanostatic charge–discharge experiments, but also resulted in improved solubility, likely due to increased steric repulsion reducing intermolecular pi-stacking (Fig. 3). A flow battery was setup under identical conditions as that with A (*N*-MEEEPhePhtha-1) and polarity reversals were similarly performed every 50 cycles. During flow operation, a peak utilization rate of 69.6% was obtained after 4 cycles. Consistent with the static H-cell data, D (*N*-MEEE*t*BuPhePhtha-1) showed significantly higher flow cycling stability with a capacity retention of 83.0% after 100 cycles. This retention represents a doubling of flow cycling stability as compared to A (*N*-MEEEPhePhtha-1) (64.3% capacity retention after 100 cycles). Flow cycling continued with D (*N*-MEEE*t*BuPhePhtha-1) for a total of 275 cycles, including 5 polarity reversals, with an average coulombic efficiency of 93.6%, an average energy efficiency of 86.8%, and a 275-cycle capacity retention of 68.6% (Fig. 8a). Post-cycling CVs indicate an average loss in concentration of ROM of just 17% over these 275 cycles based upon a comparison of peak currents before and after cycling (Fig. 8c). The higher capacity loss (31.4%) over 275 cycles as compared to the reduction in concentration of ROM (17%) can be explained *via* small leaks observed during the operation of the flow cell as well as by the potential formation of insoluble deposits on/within the separator and electrodes. The formation of such deposits is supported by electrochemical impedance spectroscopy which shows a consistent increase in cell resistance over the course of flow cell operation (Fig. 8d). There is some preliminary evidence from post cycling visual examination of membranes (see ESI Fig. SI-28†) that this fouling may be originating from the radical anion through either a direct interaction with the membrane or *via* a slowly accumulating decomposition product. This suggests that future work on optimizing this class of BRMs should focus on further improvements in radical anion stability as well as on better understanding causes of and strategies to mitigate membrane and electrode fouling.

## Conclusions

In conclusion, we successfully implemented a strategy of electronically coupling phenothiazine catholytes with phthalimide anolytes by fusing a commonly shared aromatic ring to develop a new class of bipolar redox active molecules. The resulting PhenPhtha class of molecules possesses an increased cell potential when compared to the uncoupled parent molecules, with much of this improvement resulting from an increase in oxidation potential. Structure–activity studies found that solubility of these compounds could be greatly enhanced through the incorporation of a polyether group while both stability and solubility could be improved through the incorporation of a bulky *t*-butyl group. Successful symmetric battery performance was demonstrated under relevant flow conditions utilizing two PhenPhtha molecules. In the case of D (*N*-MEEE*t*BuPhePhtha-1), high levels of stability and efficiency were observed over 275 cycles and the bipolar nature of these molecules was highlighted through five polarity reversals within this demonstration. Finally, the hybrid nature of the PhenPhtha molecules allowed for a direct comparison to be made between symmetric redox flow batteries featuring BRMs and asymmetric flow batteries featuring distinct catholytes and anolytes separated on opposing sides of the battery. These results provide direct experimental support for the superior performance of symmetric battery construction in O-NRFBs which results from eliminating significant and irreversible capacity loss associated with crossover of ROMs in asymmetric cell constructions.

## Data availability

Experimental setups and procedures, characterization data, and computational details are available in the ESI.†

## Author contributions

Conceptualization of the project was performed by Jacob Tracy. Experiments were planned and conducted by Jacob Tracy and Elena Horst under the supervision of F. Dean Toste. Calculations were performed by Vladislav Roytman. Writing was done by Jacob Tracy and Elena Horst with review by F. Dean Toste.

## Conflicts of interest

There are no conflicts to declare.

## Supplementary Material

SC-013-D2SC03450F-s001
