# Supplementary material for: Development of high-voltage bipolar redox-active organic molecules through the electronic coupling of catholyte and anolyte structures
Source: Chem Sci. 2022 Sep 1;13(36):10806–14. doi: 10.1039/d2sc03450f (PMC9491095; doi:10.1039/d2sc03450f)
Supplement: SC-013-D2SC03450F-s001 [file SC-013-D2SC03450F-s001.pdf]

Electronic Supplementary Material (ESI) for Chemical Science.
This journal is © The Royal Society of Chemistry 2022

Supporting Information

Development of High-Voltage Bipolar Redox-Active Organic Molecules Through the Electronic Coupling of Catholyte and Anolyte Structures

Jacob S. Tracy,[†a,b,c] Elena S. Horst,[†b,c] Vladislav A. Roytman[b] and F. Dean Toste[*a,b,c]

[a] Chemical Science Division, Lawrence Berkeley National Laboratory, University of California
Berkeley, CA 94720-1460, United States.
[b] Department of Chemistry, University of California, Berkeley
Berkeley, CA 94720-1460, United States.
[c] Joint Center for Energy Storage Research (JCESR),
9700 South Cass Avenue, Argonne, Illinois, 60439, United States.
† These authors contributed equally to this work.
*fdtoste@berkeley.edu

Table of Contents

I. General Information……………………………………………………..S2
II. Experimental Procedures and Compound Characterization……….S3
III. Electrochemical Investigations...……………………………………S13
IV. Computational Details……...…………………………………………..S33
V. SI References……...…………………………………...……………..S45
VI. Copies of NMR Spectra…………………………………………...S46

I. General Information

All reactions were carried out in oven- or flame-dried glassware sealed with rubber septa, kept under a positive pressure of nitrogen, and stirred with a Teflon-coated magnetic stir bar. Unless otherwise noted, reagents were obtained from commercial sources and used without further purification. Dry tetrahydrofuran (THF), toluene, dimethylfomamide (DMF) and acetonitrile (MeCN) used for synthesis were obtained by passing these previously degassed solvents through activated alumina columns under argon. Reactions were monitored by thin layer chromatography (TLC) on Supelco glass backed TLC plates (210-270 μm thickness, 60 Å porosity, F-254 indicator) and visualized by UV irradiation (254 nm) or aqueous potassium permanganate solution followed by heating. Solvents were removed under reduced pressure with a rotary evaporator and compounds dried on high vacuum on a Schlenk line.

^{1}H-NMR, ^{13}C-NMR, and ^{19}F-NMR spectra were acquired with Bruker spectrometers operating at 400 or 500 MHz for ^{1}H-NMR, 126 MHz for ^{13}C-NMR and 376 MHz for ^{19}F-NMR. Chemical shifts are reported relative to the residual solvent signal ($CDCl_3$: ^{1}H-NMR: $\delta = 7.26$ ppm; ^{13}C-NMR $\delta = 77.16$ ppm or DMSO: ^{1}H-NMR: $\delta = 2.50$ ppm; ^{13}C-NMR: $\delta = 39.52$ ppm). Multiplicities were indicated with s = singlet, d = doublet, t = triplet, q = quartet, h=heptet, m = multiplet, br = broad resonance. For flash column chromatography, the automatic chromatography system CombiFlash® NextGen 100 (UV light detection: 254 nm and 280 nm) with pre-packed columns (silica gel, 40 μm, 60 Å) was used. The eluting solvents along with the corresponding linear gradients are listed individually for each compound. High-resolution mass spectra (HRMS) were acquired on a Perkin Elmer UHPLC-TOF (ESI) at the Lawrence Berkeley National Laboratory Catalysis Laboratory located in the Department of Chemistry at the University of California, Berkeley.

II. Experimental Procedures and Compound Characterization

5,6-dichloro-2-(2,6-diisopropylphenyl)isoindoline-1,3-dione (**SI-1**)

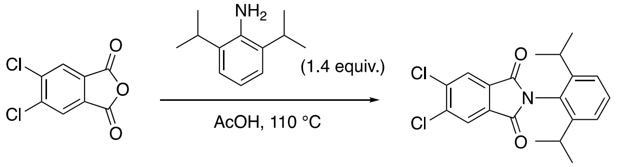

A round-bottom flask equipped with a magnetic stir bar and reflux condenser was placed under a nitrogen atmosphere and charged with 4,5-dichlorophthalic anhydride (2.17 g, 10.0 mmol) and acetic acid (22.5 mL). 2,6-Diisopropylaniline (2.61 mL, 14 mmol) was added dropwise, and the reaction mixture was stirred overnight in an oil bath at 110 °C. At this point, the reaction mixture was cooled to rt and poured into an aqueous solution of HCl (1 M, 250 mL). The resulting precipitate was collected by filtration and washed with water (500 mL). Drying the compound under vacuum afforded the product as a white solid (3.63 g, 9.65 mmol, 96%). ^{1}H-NMR (500 MHz, $CDCl_3$) δ(ppm) = 8.06 (s, 2H), 7.47 (t, *J* = 7.8 Hz, 1H), 7.29 (d, *J* = 7.8 Hz, 2H), 2.64 (h, *J* = 6.9 Hz, 2H), 1.16 (d, *J* = 6.9 Hz, 12H). ^{13}C-NMR (126 MHz, $CDCl_3$) δ (ppm) = 166.5, 147.3, 139.6, 131.1, 130.6, 126.5, 126.2, 124.2, 29.6, 24.1. R_f (*n*-hexanes/ethyl acetate 40:1) = 0.35. Data are consistent with those reported in the literature.[1]

5-((2-aminophenyl)thio)-6-chloro-2-(2,6-diisopropylphenyl)isoindoline-1,3-dione (**SI-2**)

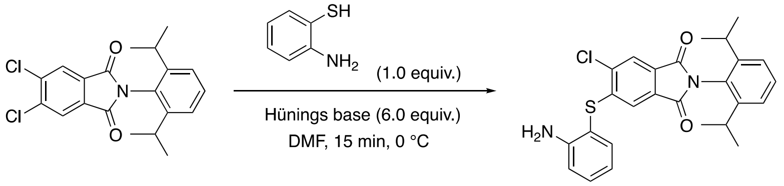

An oven-dried round-bottom flask equipped with a magnetic stir bar was charged with 5,6-dichloro-2-(2,6-diisopropylphenyl)isoindoline-1,3-dione (1.4 g, 3.7 mmol). The flask was placed under a nitrogen atmosphere and DMF (30 mL) was added. The reaction mixture was cooled to 0 °C (ice bath) and Hüning's base (3.9 mL, 22 mmol) was added followed by the dropwise addition of *o*-aminothiophenol (0.40 mL, 3.7 mmol). After stirring at 0 °C for 15 minutes, the reaction mixture was warmed to rt, diluted with water (300 mL), and extracted with dichloromethane (3 x 300 mL). The combined organic layers were washed with water (2 x 500 mL) and brine (1 x 500 mL) and dried over magnesium sulfate. The solvent was removed under reduced pressure and the crude product was purified by flash chromatography (SiO_2, ethyl acetate in *n*-hexanes, 0-20%). The product was obtained as a bright yellow solid (1.65 g, 3.55 mmol, 96%). ^{1}H NMR (400 MHz, $CDCl_3$) δ(ppm) = 7.93 (s, 1H), 7.50 – 7.42 (m, 2H), 7.35 (ddd, *J* = 8.6, 7.3, 1.6 Hz, 1H), 7.27 (d, *J* = 7.6 Hz, 2H), 7.18 (s, 1H), 6.91 – 6.81 (m, 2H), 4.79 – 3.58 (br, 2H), 2.64 (h, *J* = 6.8 Hz, 2H), 1.14 (d, *J* = 4.3 Hz, 6H), 1.12 (d, *J* = 4.4 Hz, 6H).^{13}C NMR (126 MHz, $CDCl_3$) δ 167.4, 167.1, 149.5, 147.3, 146.2, 137.9, 136.5, 133.1, 130.6, 130.4, 129.0, 126.8, 125.0, 124.1, 121.0, 119.8, 116.3, 110.0, 29.4, 24.1, 24.1. R_f (*n*-hexanes/ethyl acetate 10:1) = 0.30. Data are consistent with those reported in the literature.[1]

2-(2,6-diisopropylphenyl)pyrrolo[3,4-b]phenothiazine-1,3(2H,10H)-dione (**SI-3**)

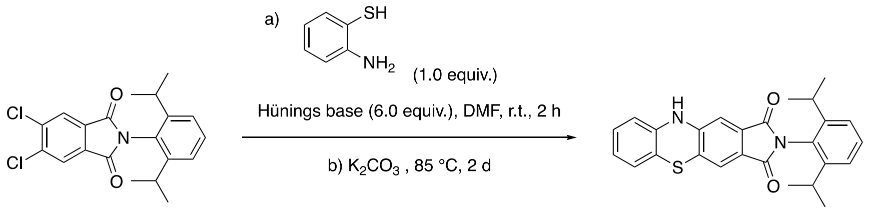

A 25 mL multi-neck round-bottom flask equipped with a magnetic stir bar was placed under a nitrogen atmosphere and charged with 5,6-dichloro-2-(2,6-diisopropylphenyl)isoindoline-1,3-dionen (376 mg, 1.00 mmol) followed by DMF (8 mL). Hüning's base (1.05 mL, 6.00 mmol) was then added followed by the dropwise addition of *o*-aminothiophenol (0.11 mL, 1.0 mmol). The reaction was stirred at room temperature for two hours at which point potassium carbonate (622 mg, 4.50 mmol) was added and the resulting suspension was heated to 65 °C via an oil bath for two days. Next, the reaction mixture was diluted with water (80 mL) and extracted with DCM (3 x 100 mL). Centrifugation was used to separate the layers. The combined organic layers were washed with water (2 x 300 mL) and brine (300 mL), dried over magnesium sulfate and the solvent was removed under reduced pressure. Purification with flash chromatography (55 g SiO_2, ethyl acetate in *n*-hexanes, 0-20%) gave the product as a red solid (170 mg, 0.40 mmol, 40%). ^{1}H NMR (400 MHz, $CDCl_3$) δ(ppm) = 7.47 (t, *J* = 7.8 Hz, 1H), 7.43 (s, 1H), 7.30 (d, *J* = 7.8 Hz, 2H), 7.23 (s, 1H), 7.09 (s, 1H), 6.91 – 6.79 (m, 3H), 6.15 – 6.09 (m, 1H), 2.71 (h, *J* = 7.0 Hz, 2H), 1.21 – 1.09 (m, 12H). ^{13}C-NMR (126 MHz, $CDCl_3$) δ (ppm) = 168.7, 167.6, 147.8, 147.6, 139.4, 132.2, 130.2, 128.2, 127.1, 126.7, 125.7, 124.8, 124.1, 122.4, 116.3, 115.2, 108.9, 29.5, 24.1, 24.1. R_f (*n*-hexanes/ethyl acetate 5:1) = 0.35. Data are consistent with those reported in the literature.[1]

2-(2,6-diisopropylphenyl)-10-methylpyrrolo[3,4-b]phenothiazine-1,3(2H,10H)-dione (***N*-MPhePhtha-1**)

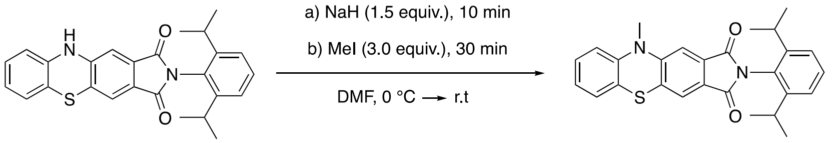

A round-bottom flask equipped with a magnetic stir bar was charged with 2-(2,6-diisopropylphenyl)pyrrolo[3,4-b]phenothiazine-1,3(2H,10H)-dione (218 mg, 0.509 mmol), placed under a nitrogen atmosphere, and DMF (5 mL) was added. Sodium hydride (60% dispersion in mineral oil, 30.8 mg, 0.770 mmol) was then added at 0 °C (ice bath) and the reaction was stirred for 10 min. At this point, methyl iodide (0.095 mL, 1.3 mmol, 3.0 equiv.) was added and the mixture was warmed to room temperature and stirred for 30 min. The reaction mixture was sparged with nitrogen to remove excess methyl iodide, diluted with water (50 mL) and extracted with DCM (3 x 50 mL) and toluene (2 x 50 mL). The combined organic layers were dried over magnesium sulfate and the solvent was removed under reduced pressure. Purification with flash chromatography (55 g SiO_2, ethyl acetate in *n*-hexanes 0 - 20%) gave the product as an orange solid (119 mg, 0.269 mmol, 53%). ^{1}H NMR (400 MHz, $CDCl_3$) δ(ppm) = 7.65 (s, 1H), 7.45 (t, *J* = 7.8 Hz, 1H), 7.29 (d, *J* = 5.2 Hz, 2H), 7.27 (s, 1H), 7.25 – 7.20 (m, 1H), 7.15 (dd, *J* = 7.7, 1.5 Hz, 1H), 7.03 (td, *J* = 7.5, 1.1 Hz, 1H), 6.88 (dd, *J* = 8.2, 1.1 Hz, 1H), 3.49 (s, 3H), 2.69 (h, *J* = 6.8 Hz, 2H), 1.15 (d, *J* = 6.8 Hz, 6H), 1.15 (d, *J* = 6.8 Hz, 6H). ^{13}C-NMR (126 MHz, $CDCl_3$) δ (ppm) = 168.4, 167.7, 151.7, 147.5, 144.2,

132.4, 131.1, 130.3, 128.4, 127.5, 127.2, 125.5, 124.2, 124.1, 122.6, 122.0, 115.0, 108.6, 36.4, 29.5, 24.1, 24.1. R_f (*n*-hexanes/ethyl acetate 5:1) = 0.30. Data are consistent with those reported in the literature.[1]

2-(2,6-diisopropylphenyl)-10-(2-(2-methoxyethoxy)ethyl)pyrrolo[3,4-b]phenothiazine-1,3(2H,10H)-dione (***N*-MEEPhePhtha-1**)

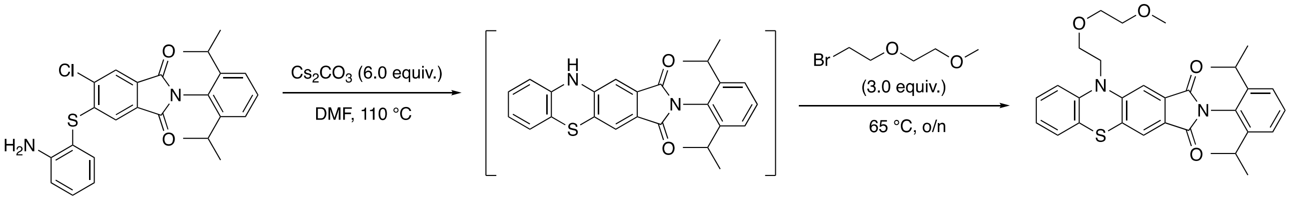

A flame-dried round-bottom flask equipped with a magnetic stir bar was charged with 5-((2-aminophenyl)thio)-6-chloro-2-(2,6-diisopropylphenyl)isoindoline-1,3-dione (1.05 g, 2.26 mmol) and cesium carbonate (4.42 g, 13.6 mmol) and placed under a nitrogen atmosphere. DMF (20 mL) was added and the reaction mixture was heated to 110 °C in an oil bath and stirred for four hours. Next, the reaction was brought to 65 °C (oil bath) and 1-bromo-2-(2-methoxyethoxy)ethane (0.91 mL, 6.8 mmol) was added. The suspension was stirred overnight at this temperature, diluted with water (200 mL) and extracted with DCM (3 x 250 mL). The combined organic layers were washed with brine, dried over magnesium sulfate and the solvent was removed under reduced pressure. The crude product was purified *via* flash chromatography (80 g SiO_2, ethyl acetate in *n*-hexanes, 0-30%) and obtained as an orange solid (970 mg, 1.80 mmol, 80%). ^{1}H-NMR (500 MHz, $CDCl_3$) δ(ppm) = 7.63 (s, 1H), 7.46 – 7.40 (m, 2H), 7.27 (d, J = 7.9 Hz, 2H), 7.20 (dd, J = 1.7, 0.8 Hz, 1H), 7.12 (dd, J = 7.7, 1.5 Hz, 1H), 7.01 (td, J = 7.5, 1.1 Hz, 1H), 6.97 (dd, J = 8.3, 1.1 Hz, 1H), 4.21 (t, J = 6.0 Hz, 2H), 3.92 (t, J = 6.0 Hz, 2H), 3.70 – 3.63 (m, 2H), 3.58 – 3.51 (m, 2H), 3.37 (s, 3H), 2.69 (h, J = 6.8 Hz, 2H), 1.15 (d, J = 6.8 Hz, 6H), 1.15 (d, J = 6.8 Hz, 6H). ^{13}C-NMR (126 MHz, $CDCl_3$) δ (ppm) = 168.2, 167.7, 151.1, 147.5, 143.4, 132.3, 132.1, 130.3, 128.3, 127.7, 127.2, 125.6, 124.4, 124.1, 123.3, 122.9, 116.3, 109.8, 72.1, 70.9, 68.4, 59.3, 48.7, 29.5, 24.1. R_f (*n*-hexanes/ethyl acetate 3.5:1) = 0.30. HRMS (ESI) calculated for $C_{31}H_{34}N_2O_4S$ (H+): 531.2312, found: 531.2306.

2-(2-(2,6-diisopropylphenyl)-1,3-dioxo-2,3-dihydropyrrolo[3,4-*b*]phenothiazin-10(1*H*)-yl)-*N*,*N*,*N*-trimethylethan-1-aminium hexafluorophosphate (***N*-(NMe$_4$)EPhePhtha-1**)

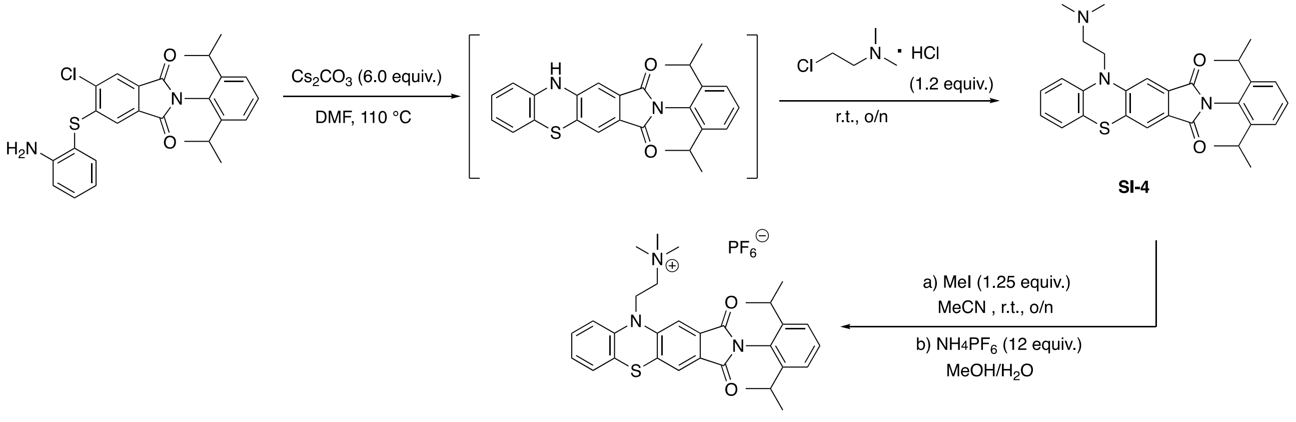

2-(2,6-diisopropylphenyl)-10-(2-(dimethylamino)ethyl)pyrrolo[3,4-b]phenothiazine-1,3(2H,10H)-dione **(SI-4)**: An oven-dried round-bottom flask equipped with a magnetic stir bar was charged with 5-((2-aminophenyl)thio)-6-chloro-2-(2,6-diisopropylphenyl)isoindoline-1,3-dione (600 mg, 1.30 mmol) and cesium carbonate (2.54 g, 7.80 mmol) and placed under a nitrogen atmosphere. DMF (15 mL) was added and the suspension was heated to 110 °C (oil bath) and stirred for 2 hours. At this point, the reaction flask was cooled to room temperature and 2-dimethylaminoethyl chloride hydrochloride (225 mg, 1.56 mmol) was added. The reaction mixture was stirred overnight at room temperature at which point it was diluted with an aqueous solution of sodium bicarbonate (20% saturated, 150 mL) and extracted with ethyl acetate (3 x 300mL). The combined organic layers were washed with brine (200 mL), dried with magnesium sulfate and the solvent was removed under reduced pressure. Purification with flash chromatography (80 g SiO_2, methanol in ethyl acetate, 0-10%) afforded the product as an orange solid (375 mg, 0.750 mmol, 58%). ^{1}H NMR (400 MHz, $CDCl_3$) δ (ppm) = 7.64 (s, 1H), 7.45 (t, *J* = 7.8 Hz, 1H), 7.40 (s, 1H), 7.28 (d, *J* = 7.9 Hz, 2H), 7.22 (td, *J* = 7.7, 1.6 Hz, 1H), 7.14 (dd, *J* = 7.8, 1.6 Hz, 1H), 7.07 – 6.98 (m, 2H), 4.09 (t, *J* = 6.7 Hz, 2H), 2.77 (t, *J* = 6.6 Hz, 2H), 2.68 (h, *J* = 6.8 Hz, 2H), 2.34 (s, 6H), 1.15 (d, *J* = 6.8 Hz, 6H), 1.15 (d, *J* = 6.8 Hz, 6H). R_f (ethyl acetate/methanol 9:1) = 0.30.

2-(2-(2,6-diisopropylphenyl)-1,3-dioxo-2,3-dihydropyrrolo[3,4-*b*]phenothiazin-10(1*H*)-yl)-*N*,*N*,*N*-trimethylethan-1-aminium hexafluorophosphate (***N*-(NMe$_4$)EPhePhtha-1**): An oven-dried round-bottom flask was charged with **SI-4** (320 mg, 0.640 mmol) and placed under a nitrogen atmosphere. Acetonitrile (10 mL) was added followed by methyl iodide (0.050 mL, 0.80 mmol). The suspension became a homogenous solution and the reaction was allowed to stir overnight at room temperature. The volatiles were then removed with a flow of nitrogen and the residue was dissolved in a mixture of water (4 mL) and methanol (35 ml). Ammonium hexafluorophosphate (1.21 g, 7.42 mmol) and water (4mL) were added and the yellow precipitate was filtered and washed with water. Drying the precipitate under vacuum afforded the product as a yellow solid (307 mg, 0.465 mmol, 73 %). ^{1}H NMR (400 MHz, DMSO) δ (ppm) = 7.91 (s, 1H), 7.83 (s, 1H), 7.49 (t, *J* = 7.8 Hz, 1H), 7.42 – 7.28 (m, 5H), 7.17 (t, *J* = 7.4 Hz, 1H), 4.61 (t, *J* = 6.4 Hz, 2H), 3.72 (t, *J* = 6.3 Hz, 2H), 3.17 (s, 9H), 2.62 (h, *J* = 6.8 Hz, 2H), 1.07 (d, *J* = 2.4 Hz, 6H), 1.05 (d, *J* = 2.4 Hz, 6H). ^{19}F NMR (376 MHz, DMSO) δ -70.1 (d, J_{P-F} = 711.3 Hz). ^{13}C NMR (126 MHz, DMSO) δ (ppm) = 167.5, 166.9, 151.1, 146.9, 142.4, 132.6, 131.6, 130.1, 128.7, 128.0, 127.0, 125.4, 124.9, 123.9,

123.8, 123.2, 117.3, 111.6, 61.8, 52.8, 40.9, 28.7, 23.6, 23.6. R_f (ethyl acetate/methanol 9:1) = 0.10. HRMS (ESI) calculated for $C_{31}H_{36}N_3O_2S$ (+): 513.2523, found: 513.2510.

<u>2-(2,6-diisopropylphenyl)-10-(2-(2-(2-methoxyethoxy)ethoxy)ethyl)pyrrolo[3,4-*b*]phenothiazine-1,3(2*H*,10*H*)-dione (***N*-MEEEPhePhtha-1**)</u>

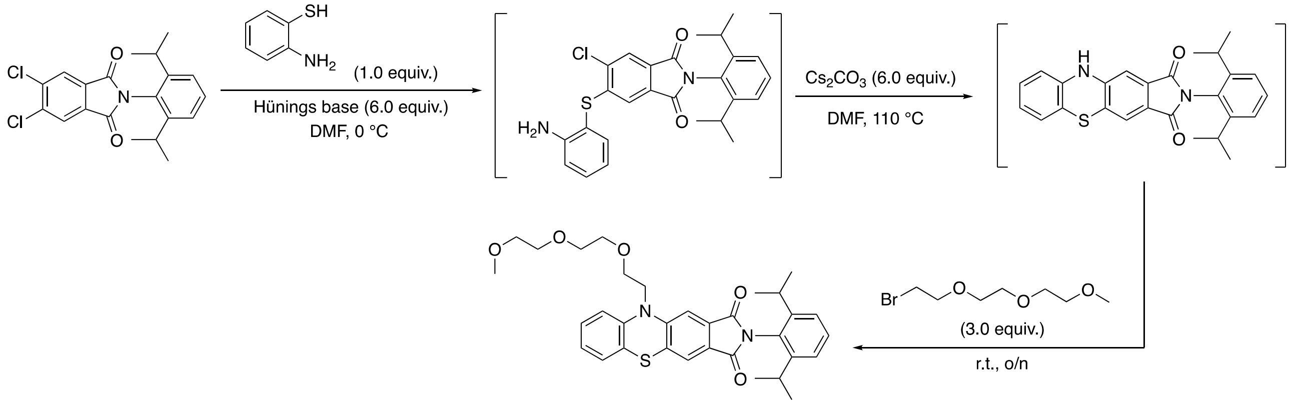

An oven-dried round-bottom flask equipped with a magnetic stir bar under a nitrogen atmosphere was charged with 5,6-dichloro-2-(2,6-diisopropylphenyl)isoindoline-1,3-dione (100 mg, 0.266 mmol) and DMF (27 mL). Hüning's base (0.28 mL, 0.27 mmol) was added, the solution was cooled to 0 °C (ice bath), and *o*-aminothiophenol (0.028 mL, 0.27 mmol) was added dropwise. The reaction mixture was stirred for 15 minutes, then cesium carbonate (520 mg, 1.6 mmol) was added and the suspension was heated to 110 °C (oil bath). Stirring for one hour gave a dark green reaction mixture, which was brought to 65 °C (oil bath). Next, 1-bromo-2-(2-(2-methoxyethoxy)ethoxy)ethane (0.14 mL, 0.80 mmol) was added and the reaction was stirred overnight at 65 °C (oil bath). The red suspension was diluted with water (270 mL) and then extracted with ethyl acetate (3 x 300 mL). The combined organic layers were washed with brine (300 mL), dried over magnesium sulfate and the volatiles were removed under reduced pressure. Purification with flash chromatography (1st column: 55 g SiO_2, ethyl acetate in toluene, 15-35%; 2nd column: 24 g SiO_2, ethyl acetate in toluene 15-35%) gave the product as an orange solid (123 mg, 0.214 mmol, 80%). ^{1}H NMR (500 MHz, $CDCl_3$) δ (ppm) = 7.62 (s, 1H), 7.44 (t, J = 7.8 Hz, 1H), 7.41 (s, 1H), 7.27 (d, J = 7.9 Hz, 2H), 7.20 (td, J = 7.8, 1.6 Hz, 1H), 7.12 (dd, J = 7.7, 1.5 Hz, 1H), 7.01 (td, J = 7.5, 1.0 Hz, 1H), 6.97 (d, J = 8.2 Hz, 1H), 4.20 (t, J = 6.0 Hz, 2H), 3.92 (t, J = 6.0 Hz, 2H), 3.72 – 3.60 (m, 6H), 3.55 – 3.49 (m, 2H), 3.36 (s, 3H), 2.77 – 2.60 (m, 2H), 1.15 (d, J = 6.7 Hz, 6H), 1.15 (d, J = 6.7 Hz, 6H). ^{13}C NMR (126 MHz, $CDCl_3$) δ (ppm) =168.2, 167.6, 151.1, 147.5, 143.4, 132.3, 132.1, 130.3, 128.3, 127.7, 127.2, 125.5, 124.4, 124.1, 123.3, 122.9, 116.3, 109.8, 72.1, 71.0, 70.8, 70.8, 68.3, 59.2, 48.7, 29.5, 24.1. R_f (*n*-hexanes/ethyl acetate 5:1) = 0.30. HRMS (ESI) calculated for $C_{33}H_{38}N_2O_5S$ (Na^+) 597.2394, found: 597.2392.

2-amino-5-(*tert*-butyl)benzenethiol (**SI-5**)

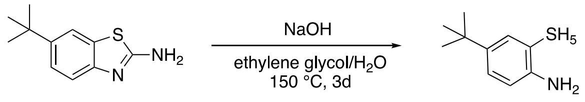

A 250 mL round bottom flask was charged with 2-amino-6-tert-butylbenzothiazole (1.00 g, 4.85 mmol) and placed under a nitrogen atmosphere. After dissolving the solid in ethylene glycol (23 mL), an aqueous solution of sodium hydroxide (18 mL, 10 N) was added to give a suspension. Heating to 135 °C (oil bath) gave a clear solution which was stirred for three days at this elevated temperature. The reaction mixture was brought to room temperature and acidified to pH=6 (aq. HCl, 1M) and extracted with DCM (3 x 150 mL). The combined organic layers were washed with brine (150 mL), dried over MgSO4 and the volatiles were removed under reduced pressure. Purification with flash chromatography (40 g SiO_2, ethyl acetate in *n*-hexane, 0 – 30%) gave the product as a yellow solid (550 mg, 3.05 mmol, 63%). ^{1}H NMR (400 MHz, $CDCl_3$) δ(ppm) = 7.21 – 7.09 (m, 2H), 6.66 (dd, J = 7.6, 1.2 Hz, 1H), 4.15 (s, 2H), 1.17 (s, 9H). R_f (*n*-hexanes/ethyl acetate 3:1) = 0.30. Data are consistent with that reported in the literature.[2]

7-(*tert*-butyl)-2-(2,6-diisopropylphenyl)-10-(2-(2-(2-methoxyethoxy)ethoxy)ethyl)pyrrolo[3,4-*b*]phenothiazine-1,3(2*H*,10*H*)-dione (***N*-MEEE*t*BuPhePhtha-1**)

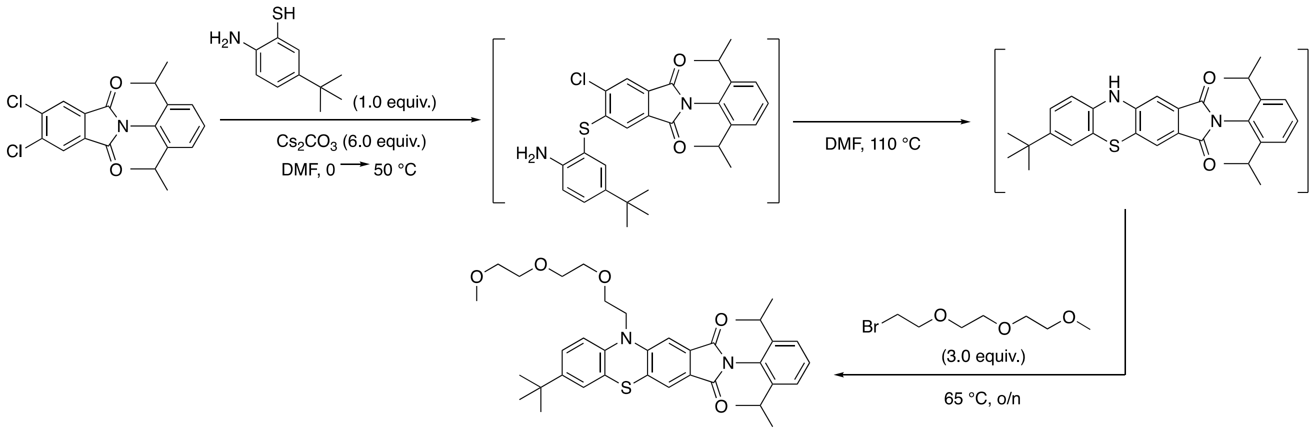

An oven-dried round-bottom flask equipped with a magnetic stir bar under a nitrogen atmosphere was charged with 5,6-dichloro-2-(2,6-diisopropylphenyl)isoindoline-1,3-dione (56 mg, 0.15 mmol), 2-amino-5-(tert-butyl)benzenethiol (27 mg, 0.15 mmol), and DMF (1.5 mL). The solution was cooled to 0 °C (ice bath) and cesium carbonate (290 mg, 0.90 mmol) was added. The reaction mixture was allowed to warm to room temperature and stirred overnight, then heated to 50 °C (oil bath) and stirred for another night. After that, stirring for one hour at 110 °C (oil bath) gave a dark green reaction mixture which was brought to 65 °C (oil bath). At this point, 1-bromo-2-(2-(2-methoxyethoxy)ethoxy)ethane (0.080 mL, 0.45 mmol) was added and the reaction was stirred for another night at 65 °C (oil bath). The suspension was brought to room temperature, diluted with water (15 mL) and extracted with ethyl acetate (3 x 20 mL). The combined organic layers were washed with brine (100 mL), dried over magnesium sulfate and the volatiles were removed under reduced pressure. Purification with flash chromatography (1st column: 40 g SiO_2 ethyl acetate in *n*-hexanes, 0 – 30%, 2nd column: 24 g SiO_2, ethyl acetate in toluene 0 – 20%) gave the product as

an orange solid (47 mg, 0.075 mmol, 50%). ^{1}H NMR (500 MHz, $CDCl_3$) δ (ppm) = 7.62 (s, 1H), 7.44 (s, 1H), 7.38 (s, 1H), 7.27 (d, *J* = 8.1 Hz, 2H), 7.20 (dd, *J* = 8.6, 2.3 Hz, 1H), 7.12 (d, *J* = 2.2 Hz, 1H), 6.90 (d, *J* = 8.6 Hz, 1H), 4.18 (t, *J* = 6.0 Hz, 2H), 3.91 (t, *J* = 6.0 Hz, 2H), 3.71 – 3.60 (m, 6H), 3.55 – 3.51 (m, 2H), 3.36 (s, 3H), 2.69 (h, *J* = 6.8 Hz, 2H), 1.29 (s, 9H), 1.15 (d, *J* = 6.8 Hz, 6H), 1.15 (d, *J* = 6.8 Hz, 6H). ^{13}C NMR (126 MHz, $CDCl_3$) δ (ppm) = 168.3, 167.7, 151.2, 147.8, 147.5, 140.7, 132.3, 131.9, 130.3, 127.2, 125.2, 125.2, 124.8, 124.1, 122.9, 122.5, 115.8, 109.4, 72.1, 71.0, 70.8, 70.8, 68.3, 59.2, 48.6, 34.4, 31.4, 29.5, 24.1. R_f (*n*-hexanes/ethyl acetate 4:1) = 0.35. HRMS (ESI) calculated for $C_{37}H_{46}N_2O_5S$ (Na^+) 653.3020, found: 653.3025.

5,6-dichloro-2-(1-(2-methoxyethoxy)propan-2-yl)isoindoline-1,3-dione (**SI-6**)

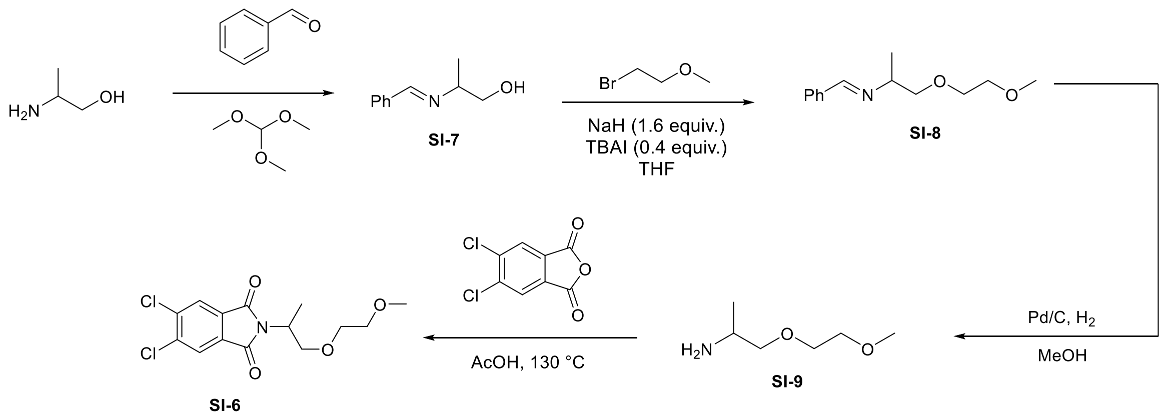

(E)-2-(benzylideneamino)propan-1-ol (**SI-7**): A round-bottom flask was charged with alaninol (8.0 mL, 100 mmol), trimethyl orthoformate (100 mL), and benzaldehyde (10.7 mL, 105 mmol). The reaction was stirred at room temperature overnight at which point the reaction mixture was diluted with DCM (200 mL) and washed with aqueous sodium bicarbonate (saturated, 2 x 80 mL) and brine (80 mL). The resulting organic layer was dried with magnesium sulfate and the solvent was removed under reduced pressure. The crude product was carried forward to the next step. ^{1}H NMR (400 MHz, CDCl3) δ 8.32 (s, 1H), 7.75 – 7.66 (m, 2H), 7.43 – 7.36 (m, 3H), 3.72 – 3.65 (m, 2H), 3.51 (m, 1H), 2.29 (s, 1H), 1.23 – 1.18 (m, 3H). Data are consistent with that reported in the literature.[3]

(E)-N-(1-(2-methoxyethoxy)propan-2-yl)-1-phenylmethanimine (**SI-8**): The crude product **SI-7** (4.6 g) was measured into an oven-dried three-neck round bottom flask and placed under a nitrogen atmosphere. THF (50 mL), tetra-*n*-butylammonium iodide (TBAI) (3.69 g, 10.0 mmol) and 1-bromo-2-methoxyethane (2.82 mL, 30.0 mmol) were then added. The reaction mixture was cooled to 0 °C (ice bath) and sodium hydride (1.6 g, 40 mmol) was added portion-wise. The reaction mixture was then brought to room temperature and stirred overnight. Filtration through Celite and evaporation of the solvent under reduced pressure gave the crude product in 50% purity with the remainder being unalkylated **SI-7**. The crude product was carried forward without purification or characterization.

1-(2-methoxyethoxy)propan-2-amine (**SI-9**): The crude product of **SI-8** (5.6 g) was transferred into a three-neck round bottom flask and placed under a nitrogen atmosphere. Methanol (50 mL) was added followed by palladium on activated carbon (10% wt palladium, 560 mg). The reaction atmosphere was then exchanged for hydrogen and allowed to stir overnight. At this point more palladium on activated carbon

(10% wt palldium, 560 mg) was added to the reaction mixture, a hydrogen atmosphere was reestablished, and the suspension was stirred for another two days. Filtration through a celite plug (with methanol as the eluting solvent) and concentration under reduced pressure gave the product in 66% purity which was carried forward as an impure mixture without further purification or characterization.

<u>5,6-dichloro-2-(1-(2-methoxyethoxy)propan-2-yl)isoindoline-1,3-dione</u> (**SI-6**): A 250 mL round-bottom flask equipped with a magnetic stir bar and reflux condenser was charged with 4,5-dichlorophthalic anhydride (3.32 g, 15.2 mmol) and placed under a nitrogen atmosphere. Acetic acid (38 mL) was added followed by the crude product **SI-9** (1.86 g, 66% purity, 11.0 mmol). The reaction mixture was heated to 110 °C (oil bath) and allowed to stir for 18 h at which point it was cooled to room temperature. The reaction mixture was then poured into an aqueous solution of hydrochloric acid (1M, 300 mL). The aqueous solution was extracted with toluene (3 x 300 mL) and the combined organic layers were washed with brine (250 mL), dried over magnesium sulfate and concentrated under reduced pressure. The crude material was purified *via* successive flash chromatography (1st column:135 g SiO_2, ethyl acetate in *n*-hexanes, 0 – 20%, 2nd column: 80 g SiO_2, ethyl acetate in *n*-hexanes 0 – 20%) to afford the product as an off-white solid (1.8 g, 5.4 mmol, 49%). ^{1}H NMR (400 MHz, $CDCl_3$) δ (ppm) = 7.89 (s, 2H), 4.69 – 4.48 (m, 1H), 4.07 – 3.93 (m, 1H), 3.69 – 3.56 (m, 2H), 3.56 – 3.48 (m, 1H), 3.46 – 3.38 (m, 2H), 3.26 (s, 3H), 1.49 – 1.34 (m, 3H). ^{13}C NMR (126 MHz, $CDCl_3$) δ (ppm) = 166.6, 138.8, 131.4, 125.3, 71.9, 71.4, 70.3, 59.1, 47.0, 15.0. R_f (*n*-hexanes/ethyl acetate 4:1) = 0.30. HRMS (ESI) calculated for $C_{14}H_{15}Cl_2NO_4$ (K^+) 370.0010, found: 370.0007.

<u>5-((2-aminophenyl)thio)-6-chloro-2-(1-(2-methoxyethoxy)propan-2-yl)isoindoline-1,3-dione</u> (**SI-10**)

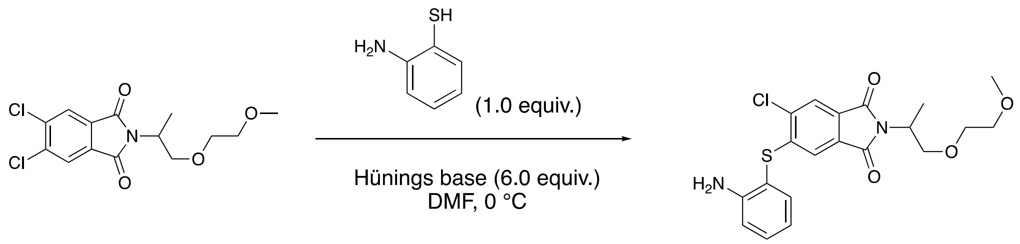

An oven-dried round-bottom flask was charged with 5,6-dichloro-2-(1-(2-methoxyethoxy)propan-2-yl)isoindoline-1,3-dione (**SI-9**) (500 mg, 1.5 mmol) and placed under a nitrogen atmosphere. DMF (12 mL) was added followed by Hüning's base (1.57 mL, 9.00 mmol) at which point the reaction mixture was cooled to 0°C (ice bath). 2-Aminothiophenol (0.16 mL, 1.5 mmol) was then added dropwise and the solution was stirred at 0°C for 5 minutes. The reaction mixture was diluted with water (120 mL) and extracted with DCM (3 x 150 mL). The combined organic layers were washed with brine (150 mL), dried over magnesium sulfate and the solvent was removed under reduced pressure. Flash chromatography (55 g SiO_2, ethyl acetate in *n*-hexane, 0 – 30%) afforded the product as an orange solid (580 mg, 1.4 mg, 93%) which was immediately carried forward to the next step after establishing purity via a ^{1}H NMR. ^{1}H NMR (400 MHz, $CDCl_3$) δ(ppm) = 7.74 (s, 1H), 7.46 – 7.41 (m, 1H), 7.39 – 7.31 (m, 1H), 7.01 (s, 1H), 6.96 – 6.85 (m, 2H), 4.60 – 4.46 (m, 1H), 4.00 – 3.92 (m, 1H), 3.65 – 3.56 (m, 2H), 3.53 – 3.45 (m, 1H), 3.44 – 3.38 (m, 2H), 3.26 (s, 3H), 1.41 – 1.33 (m, 3H). R_f (*n*-hexanes/ethyl acetate 4:1) = 0.25.

10-(2-(2-methoxyethoxy)ethyl)-2-(1-(2-methoxyethoxy)propan-2-yl)pyrrolo[3,4-*b*]phenothiazine-1,3(2*H*,10*H*)-dione (***N*-MEEPhePhtha-2**)

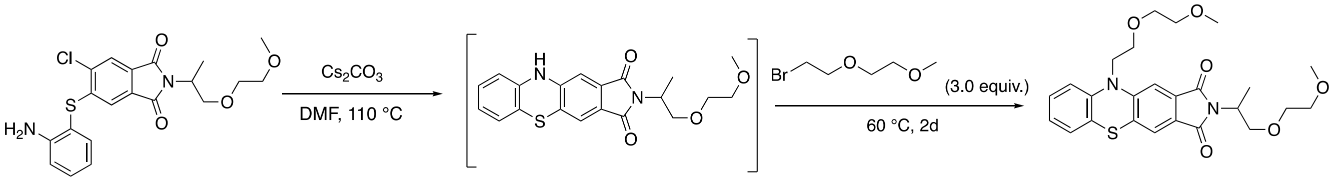

An oven-dried 100 mL round-bottom flask equipped with a magnetic stir bar was charged with **SI-10** (580 mg, 1.38 mmol) and cesium carbonate (2.7 g, 8.3 mmol) and placed under a nitrogen atmosphere. DMF (16 mL) was added and the reaction was stirred at 110°C (oil bath) for 30 minutes. After cooling the reaction to room temperature, 1-bromo-2-(2-methoxyethoxy)ethane (0.56 mL, 4.1 mmol) was added and the reaction mixture was stirred for one day at room temperature and a second day at 60°C (oil bath). Afterwards, the reaction mixture was diluted with water (160 mL) and extracted with DCM (4 x 200 mL). After letting the water layer sit for one day, a new dark red phase formed which was separated and combined with the organic layers. The combined organic layers were washed with brine (200 mL), dried over magnesium sulfate and concentrated under reduced pressure. Flash chromatography (80 g, ethyl acetate in *n*-hexanes, 0-30%) afforded the product as a red viscous oil (450 mg, 0.93 mmol, 67%). ^{1}H NMR (400 MHz, $CDCl_3$) δ (ppm) 7.46 (s, 1H), 7.25 (s, 1H), 7.22 – 7.15 (m, 1H), 7.12 – 7.07 (m, 1H), 7.03 – 6.97 (m, 1H), 6.96 – 6.91 (m, 1H), 4.63 – 4.50 (m, 1H), 4.17 (t, *J* = 6.2 Hz, 2H), 4.01 (t, *J* = 9.7 Hz, 1H), 3.86 (t, *J* = 6.1 Hz, 2H), 3.69 – 3.59 (m, 4H), 3.56 – 3.49 (m, 3H), 3.46 – 3.40 (m, 2H), 3.38 (s, 3H), 3.29 (s, 3H), 1.41 (d, *J* = 7.0 Hz, 3H). ^{13}C NMR (126 MHz, $CDCl_3$) δ (ppm) = 168.3, 167.8, 150.6, 143.5, 132.5, 131.4, 128.2, 127.7, 125.8, 124.2, 123.4, 122.2, 116.2, 109.2, 72.1, 72.0, 71.7, 70.9, 70.3, 68.3, 59.3, 59.1, 48.5, 46.4, 15.2. R_f (*n*-hexanes/ethyl acetate 1:1) = 0.25. HRMS (ESI) calculated for $C_{25}H_{30}N_2O_6S$ (•+) 486.1819, found: 486.1814.

10-(2-(2-(2-methoxyethoxy)ethoxy)ethyl)-10H-phenothiazine (**1**)

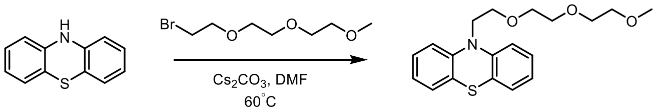

A 100 mL round-bottom flask equipped with a magnetic stir bar was charged with phenothiazine (190 mg, 1.23 mmol) and cesium carbonate (2.40 g, 7.38 mmol) and placed under a nitrogen atmosphere. DMF (10 mL) was added followed by 1-bromo-2-(2-(2-methoxyethoxy)ethoxy)ethane (0.32 mL, 1.8 mmol). The reaction mixture was stirred at 60°C for 24 h. At that point, the reaction was diluted with water (100 mL), extracted with DCM (3 x 250 mL). The combined organic layers were washed with brine (250 mL) and then dried over magnesium sulfate. The crude mixture was concentrated under reduced and purified via flash chromatography (80 g, ethyl acetate in *n*-hexanes, 0-25%) to afford the product as a clear oil (184 mg, 43%). ^{1}H NMR (400 MHz, $CDCl_3$) δ 7.18 – 7.06 (m, 4H), 6.97 – 6.83 (m, 4H), 4.10 (t, *J* = 6.4 Hz, 2H), 3.84 (t, *J* = 6.4 Hz, 2H), 3.74 – 3.59 (m, 6H), 3.57 – 3.49 (m, 2H), 3.37 (s, 3H). ^{13}C NMR (126 MHz, $CDCl_3$) δ 145.0, 127.5, 127.4, 124.7, 122.7, 115.3, 72.0, 70.8, 70.7, 70.6, 68.3, 59.1, 47.5.

2-(2,6-diisopropylphenyl)isoindoline-1,3-dione (**2**)

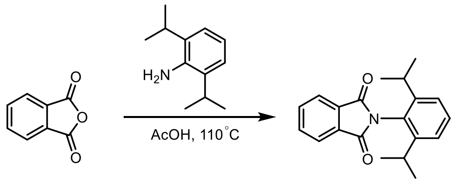

A 100 mL round-bottom flask equipped with a magnetic stir bar and reflux condenser was charged with phthalic anhydride (2.50 g, 17.0 mmol) and placed under a nitrogen atmosphere. Glacial acetic acid (40 mL) was added followed by 2,6-diisopropylaniline (4.50 mL, 23.8 mmol). The reaction mixture was heated at 110°C for 16 hours at which point it was cooled to room temperature and poured into 1 M aqueous hydrochloric acid (700 mL). The resulting white precipitate was collected via filtration and washed with water (1200 mL). After drying the solid on high-vacuum overnight, the material (4.39 g, 84%) was recrystallized from hot ethanol (40 mL) to yield a white crystalline solid (3.03 g, 58% yield). ^{1}H NMR (300 MHz, $CDCl_3$) δ 7.98 (dd, *J* = 5.5, 3.1 Hz, 2H), 7.82 (dd, *J* = 5.5, 3.1 Hz, 2H), 7.47 (dd, *J* = 8.3, 7.2 Hz, 1H), 7.30 (d, *J* = 7.7 Hz, 2H), 2.73 (h, *J* = 6.9 Hz, 2H), 1.18 (d, *J* = 6.9 Hz, 12H). ^{13}C NMR (126 MHz, $CDCl_3$) δ 168.4, 147.4, 134.5, 132.1, 130.3, 127.08, 124.1, 124.0, 29.5, 24.1. Data are consistent with that reported in the literature.[4]

Solubility measurements: Solubility measurements were conducted based upon a slightly modified version of the published method of Sanford and coworkers.[5] In short, the **PhenPhtha** derivative was added to a 1-dram vial with a magnetic stir bar containing 0.2-0.5 mL of MeCN until a solid persisted. This solution was stirred overnight with a magnetic stir bar. If no solid remained after 24 h, additional material was added and once again stirred overnight. This procedure was repeated until solid persisted for 24 h. At that point, stirring was stopped, the resulting solid was allowed to settle, and the clear solution was carefully decanted with a syringe and needle before being filtered through a 0.22 μm syringe filter (RP PTFE, Filtrous Lab Consumables) to remove residual suspended solids. The resulting saturated solution was dispensed in 5-25 μL aliquots into 3 pre-tared 1-dram vials and dried under high vacuum overnight. The resulting mass increase in the vials was converted into moles, divided by the volume dispensed, and averaged over the triplicate experiments.

Electrochemical Investigations:

General methods and materials: Acetonitrile (MeCN) (99.9%, extra dry over molecular sieves) was obtained from Thermo Scientific. Tetrabutylammonium hexafluorophosphate ($TBAPF_6$; >99%, for electrochemical analysis) was obtained from MilliporeSigma™, dried under high vacuum for 48 h at 75 °C and transferred to a N_2-filled glovebox for storage and use. All electrochemical experiments were performed in a N_2 filled glove box with an atmosphere <0.1 ppm oxygen and <0.1 ppm water. Electrolyte solutions were prepared in the glovebox by first drying the acetonitrile over freshly activated 3 Å molecular sieves for at least 24 h. Supporting electrolyte was then added and the solvent/electrolyte mixture was further dried for another 24 h before use. The resulting solvent/electrolyte mixtures were stored over the 3 Å molecular sieves in the glovebox. All potentials are reported relative to the ferrocene/ferrocenium couple (Fc/Fc^+) and this adjustment is made for each sample through the addition of a ferrocene reference at the end of each set of CV experiments.

Cyclic voltammetry: Cyclic voltammetry (CV) experiments were performed with a CH Instruments 760 Bipotentiostat with a three-electrode electrochemical cell. A glassy carbon disk electrode (BASi, 3.0 mm diameter) was used as a working electrode, a Ag/Ag^+ electrode (5 mM $AgBF_4$ in 0.5 M $TBAPF_6$ in MeCN) sealed with a Coralpor frit was used as a non-aqueous quasi-reference electrode (BASi), and platinum mesh was used as a counter electrode. The glassy carbon electrode was polished outside the glovebox using alumina (MicroPolish II, Buehler) in Milli-Q® water before being dried with acetone and brought into the glovebox. Unless otherwise indicated, all CV measurements were performed by dissolving the compound in stock 0.5 M $TBAPF_6$ in acetonitrile to give a concentration of 5 mM.

H-cell cycling: Bulk charge/discharge measurements were carried out in a nitrogen-filled glovebox with a CH Instruments 760 Bipotentiostat in a custom H-cell (pictured below) with a fritted glass separator (P5). The working and counter electrodes were carbon (Duocel® RVC Foam, 100 PPI, 3% relative density). An Ag/Ag^+ quasi-reference electrode (described above) was used on the working side of the H-cell. The active compound was dissolved in 0.5 M $TBAPF_6$ in acetonitrile to give a redox active material concentration of 5 mM. The working chamber of the H-cell was first loaded with 5 mL of the electrolyte/ROM solution while the counter chamber was loaded with 5 mL of only 0.5 M $TBAPF_6$ in acetonitrile. One charging event of the working chamber was completed at which point the solution was removed from the counter chamber and replaced with 5 mL of the electrolyte/ROM solution. A discharge event was then conducted followed by 99 more charge-discharge cycles. Charging and discharging were all conducted with a current of 5 mA and both chambers of the H-cell were continuously stirred with magnetic stir bars. The upper and lower voltage cutoffs were 0.4 V plus and minus the $E_{1/2}$ (determined with CV at a scan rate of 100 mV/s or 500 mV/s). Results of H-cell bulk electrolysis are displayed here in graphs of normalized discharge capacity (normalized relative to theoretical capacity) vs time for 100 charge-discharge cycles. Each data point in these figures represents one cycle. Use of time instead of cycle number in the figures is done in order to provide a more accurate representation of stability.[6]

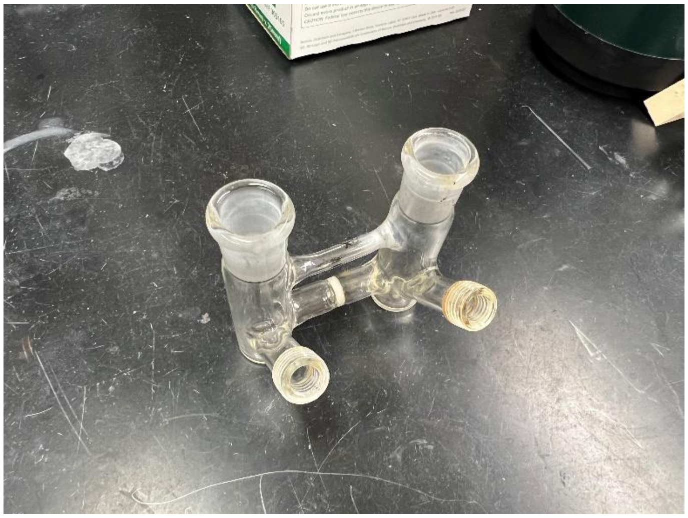

Figure SI-1: Picture of custom H-cell.

Flow Cell Cycling: Data from flow cell cycling was collected on a BioLogic VSP potentiostat (operated in a galvanostatic mode) in a nitrogen filled glovebox. The flow cell design used in these experiments was previously described in the literature by Brushett and coworkers (pictured below).[7] Briefly, the cell utilizes a zero-gap design that features graphite charge collectors built with interdigitated flow fields. Two layers of carbon felt (Sigracet® 29AA) with approximately 20% compression and with an active cross-sectional area of 2.55 cm^2 were utilized as electrodes on each side of the flow cell and held in place with ePTFA gaskets. The two sides of the flow cell were separated by a Daramic® A175 porous separator. All components of the flow cell were dried in an oven overnight, assembled outside of the glovebox, and immediately brought into the glovebox through an antechamber via a 1.5-hour evacuation/nitrogen backfill process. The assembled flow cell was allowed to equilibrate in the glovebox for 24 hours prior to use. Continuous flow was provided by a Cole-Parmer Masterflex® pump featuring a two-channel rotor through a combination of Masterflex compressible pump tubing and PFA flexible tubing. Flow rates were 20 mL/min with charge and discharge rates of 10 mA/cm^2. The upper and lower voltage cutoffs were 0.35 V plus and minus the $\Delta E_{1/2}$ (determined with CV at a scan rate of 100 mV/s). An equilibration period of 15-30 mins was utilized before active charging and discharging, during which the working solutions were flowed through the cell. Details on the loading of each chamber of the flow system are detailed in the main text. During some of the flow cell runs, small amounts of leaking were observed where the charge collectors meet the ePTFA gaskets. Future implementations of this cell design should ensure that the graphite charge collectors have a smooth finish in order to more easily seal with the ePTFA gaskets and the O-rings of the polypropylene electrolyte diffuser. Extra care should also be taken to ensure that the ePTFA gasket and charge collector interfaces remain free of blemishes during assembly and tightening of the flow cell.

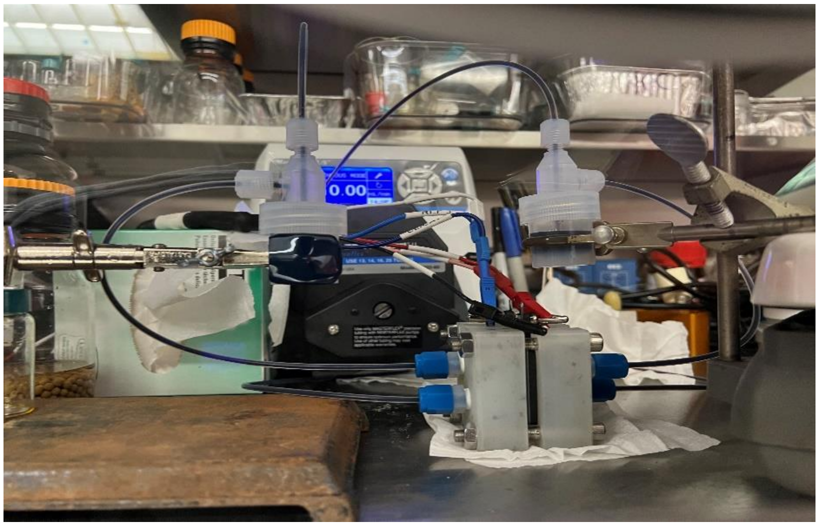

Figure SI-2: Picture of flow cell during operation.

2-(2,6-diisopropylphenyl)-10-methylpyrrolo[3,4-b]phenothiazine-1,3(2H,10H)-dione (***N*-MPhePhtha-1**)

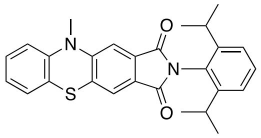

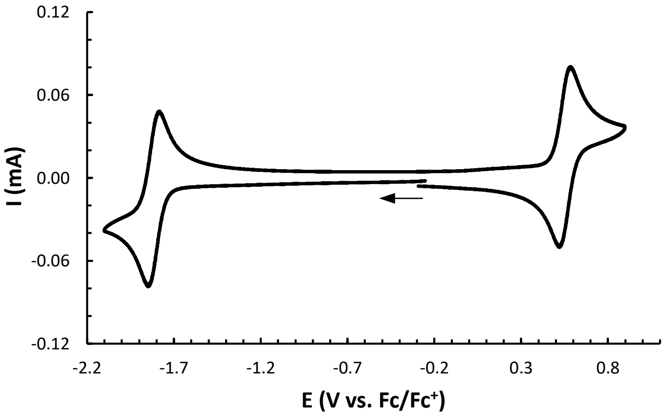

Figure SI-3: Cyclic voltammetry of ***N*-MPhePhtha-1** (<5 mM) in 0.5 M $TBAFPF_6$ in MeCN at a scan rate of 100 mV/s.

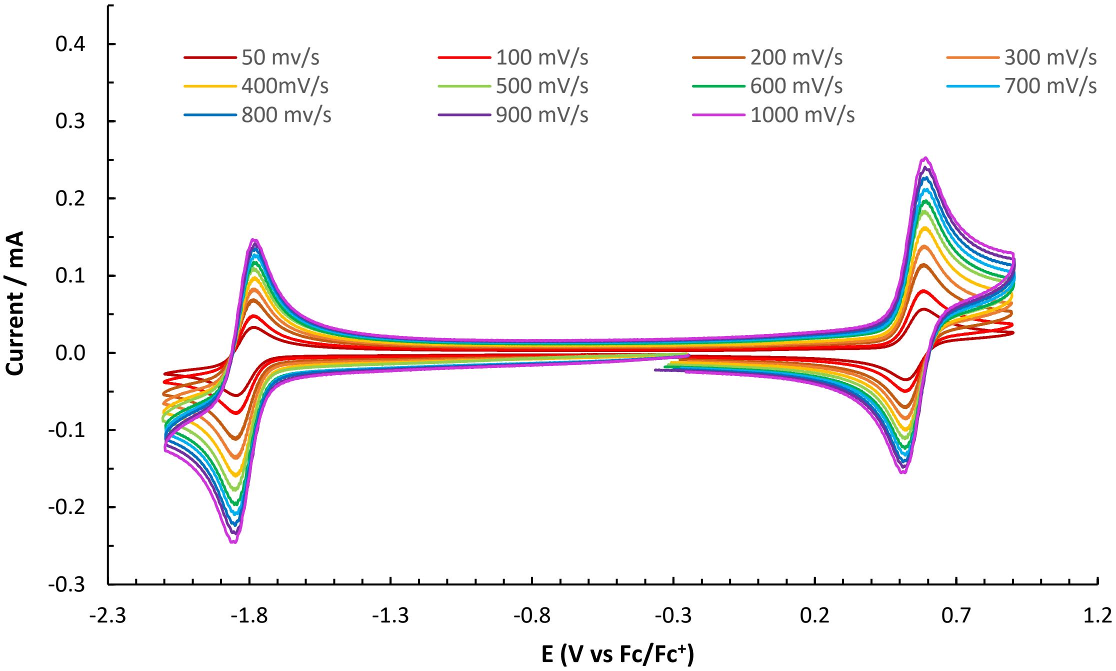

Figure SI-4: CV studies of ***N*-MPhePhtha-1** (<5 mM in 0.5 M $TBAPF_6$/MeCN) with scan rates varying from 50 to 1000 mV/s.

2-(2,6-diisopropylphenyl)-10-(2-(2-methoxyethoxy)ethyl)pyrrolo[3,4-b]phenothiazine-1,3(2H,10H)-dione (***N*-MEEPhePhtha-1**)

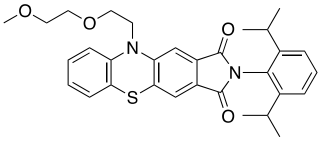

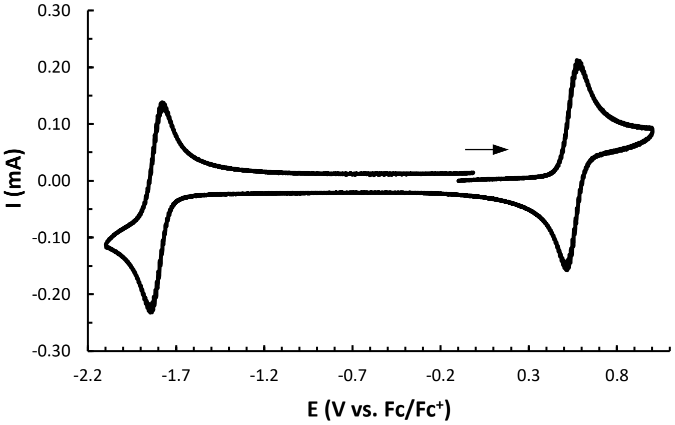

Figure SI-5: Cyclic voltammetry of ***N*-MEEPhePhtha-1**(5 mM) in 0.5 M $TBAFPF_6$ in acetonitrile at a scan rate of 500 mV/s.

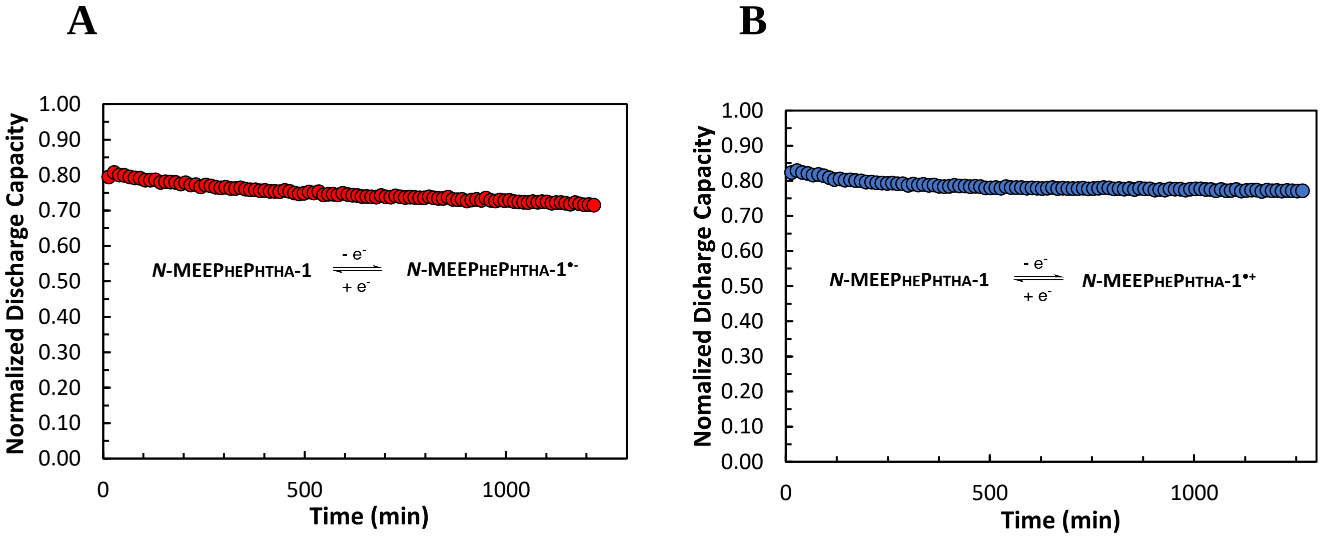

Figure SI-6: H-Cell cycling of ***N*-MEEPhePhtha-1** (5 mM) in 0.5 M $TBAFPF_6$ in acetonitrile at a current of 5 mA. A: Reductive cycling (100 cycles) and B: Oxidative cycling (100 cycles).

2-(2,6-diisopropylphenyl)-10-(2-(2-(2-methoxyethoxy)ethoxy)ethyl)pyrrolo[3,4-*b*]phenothiazine-1,3(2*H*,10*H*)-dione (***N*-MEEEPhePhtha-1**)

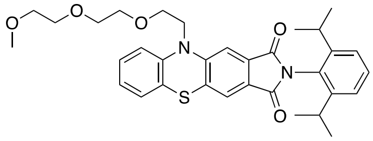

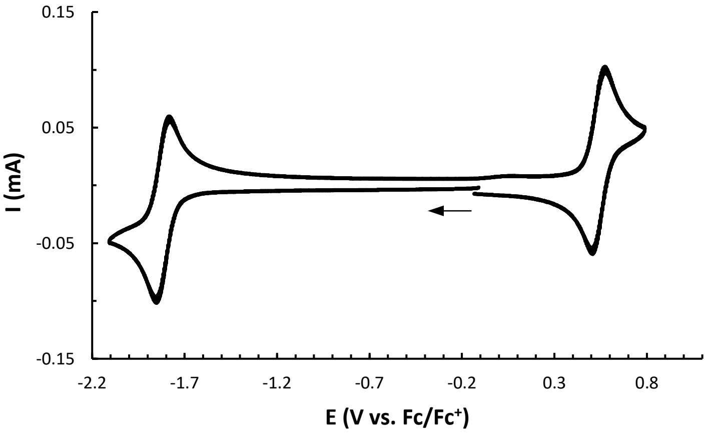

FigureSI-7: Cyclic voltammetry of ***N*-MEEEPhePhtha-1** (5 mM) at 100 mv/s in 0.5 M TBAFPF$_6$ in acetonitrile.

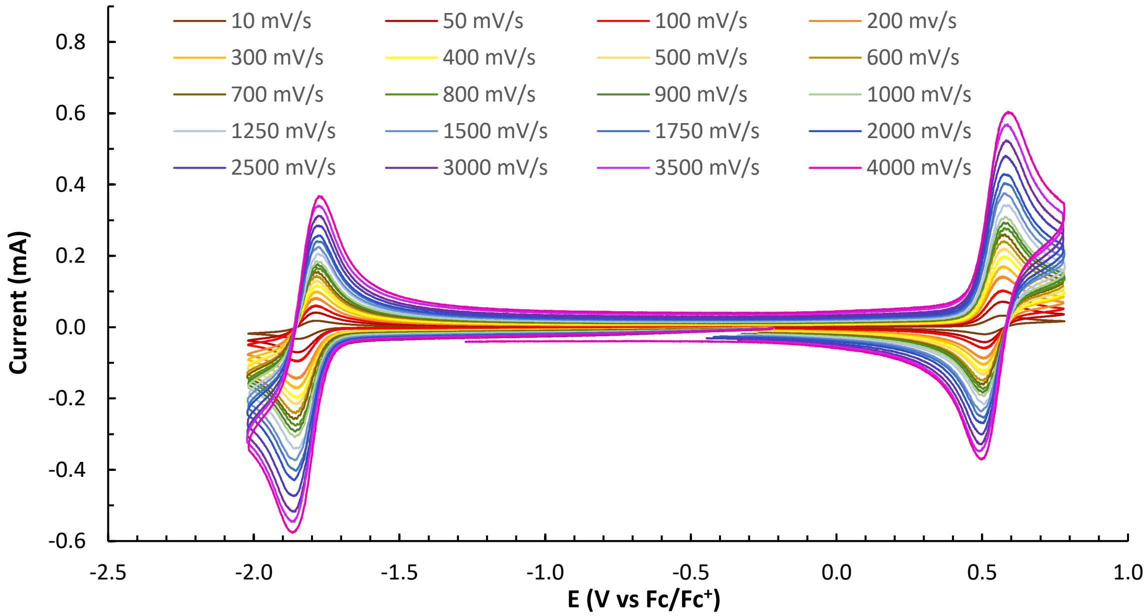

Figure SI-8: CV studies of ***N*-MEEEPhePhtha-1** (5 mM in 0.5 M TBAPF$_6$/MeCN) with scan rates varying from 10 to 4000 mV/s.

A **B**

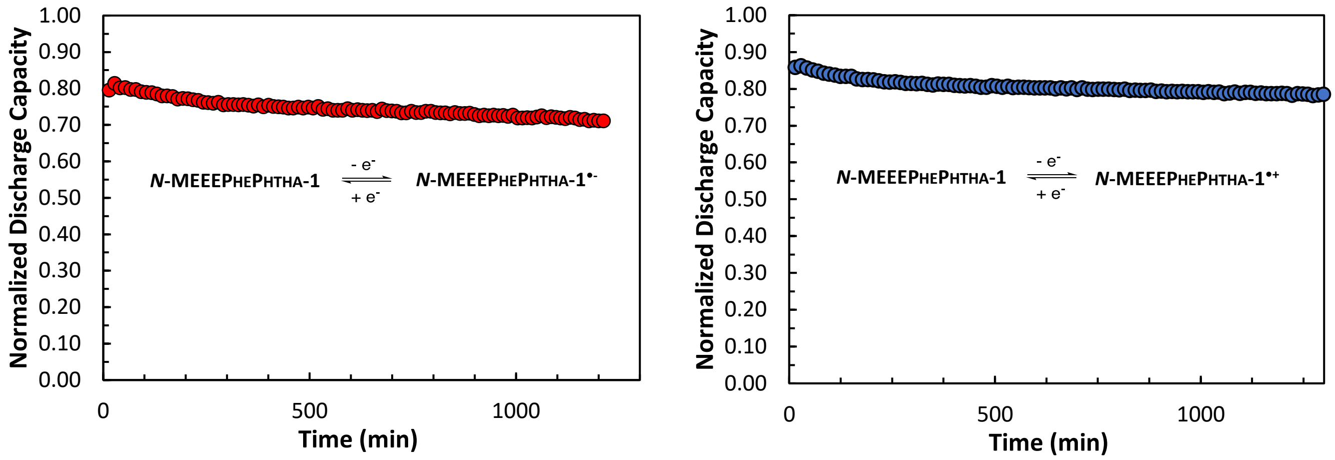

Figure SI-9: H-Cell cycling of ***N*-MEEEPhePhtha-1** (5 mM) in 0.5 M $TBAFPF_6$ in acetonitrile at a current of 5 mA. A: Reductive cycling (100 cycles) and B: Oxidative cycling (100 cycles).

10-(2-(2-methoxyethoxy)ethyl)-2-(1-(2-methoxyethoxy)propan-2-yl)pyrrolo[3,4-*b*]phenothiazine-1,3(2*H*,10*H*)-dione (***N*-MEEPhePhtha-2)**

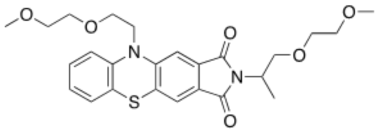

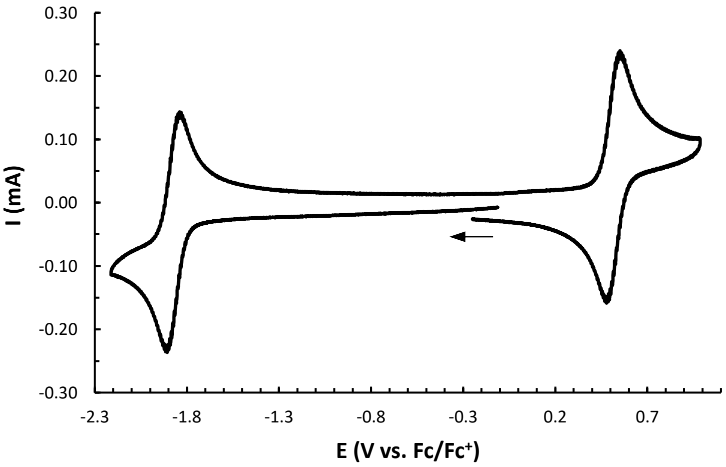

Figure SI-10: Cyclic voltammetry of ***N*-MEEPhePhtha-2** (5 mM) in 0.5 M $TBAFPF_6$ in acetonitrile at a scan rate of 500 mV/s.

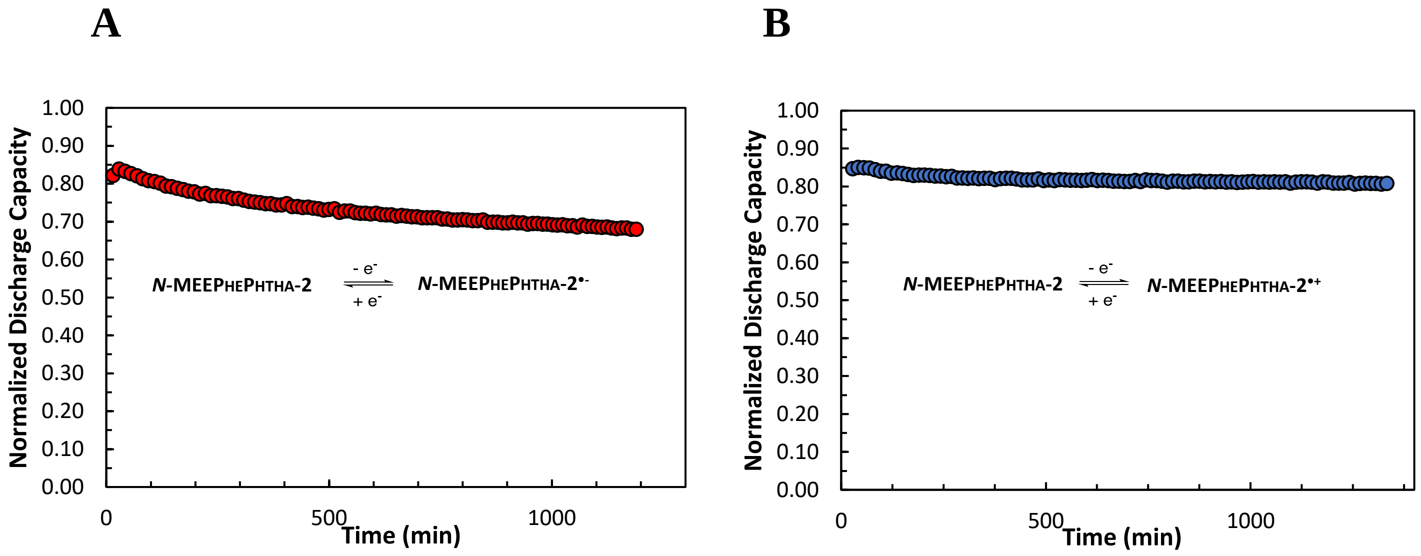

Figure SI-11: H-Cell cycling of ***N*-MEEPhePhtha-2** (5 mM) in 0.5 M $TBAFPF_6$ in acetonitrile at a current of 5 mA. A: Reductive cycling (100 cycles) and B: Oxidative cycling (100 cycles).

7-(*tert*-butyl)-2-(2,6-diisopropylphenyl)-10-(2-(2-(2-methoxyethoxy)ethoxy)ethyl)pyrrolo[3,4-*b*]phenothiazine-1,3(2*H*,10*H*)-dione (***N*-MEEE*t*BuPhePhtha-1**)

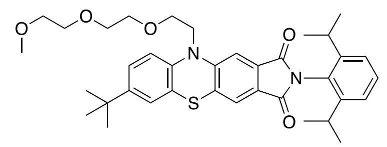

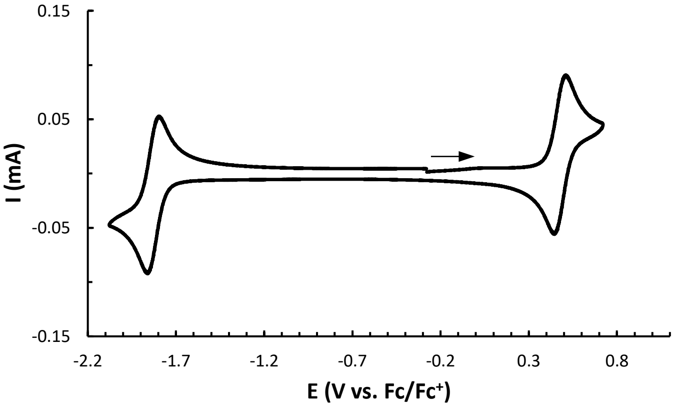

Figure SI-12: Cyclic voltammetry of ***N*-MEEE*t*BuPhePhtha-1** (5 mM) in 0.5 M $TBAFPF_6$ in acetonitrile at a scan rate of 100 mV/s.

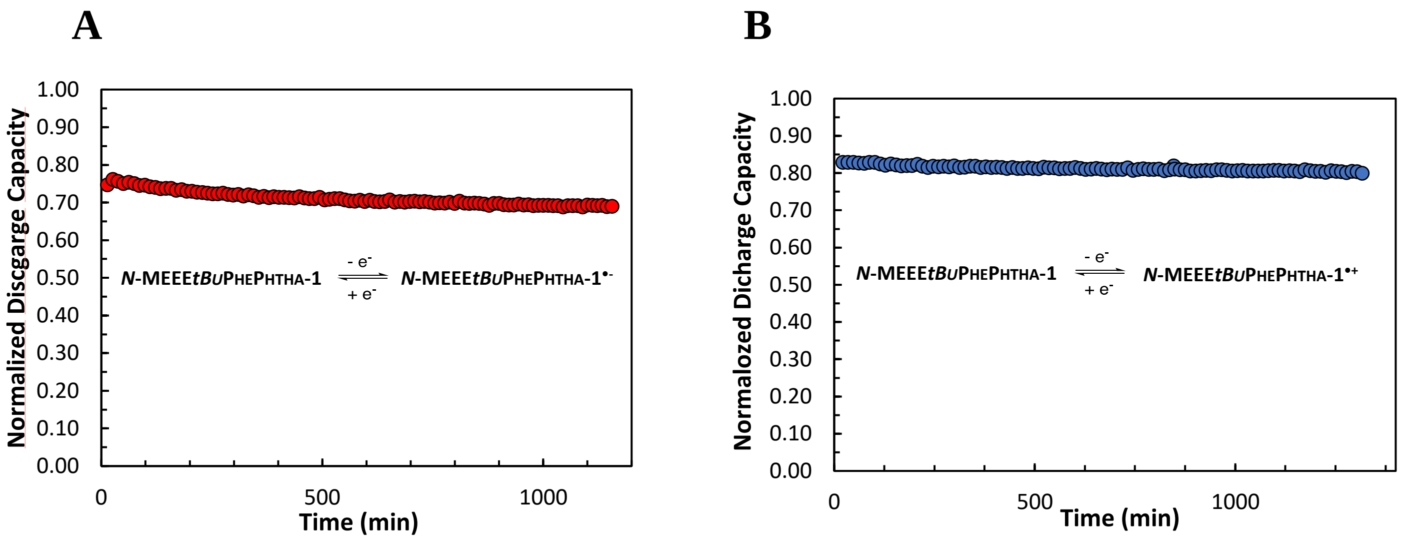

Figure SI-13: H-Cell cycling of ***N*-MEEE*t*BuPhePhtha-1** (5 mM) in 0.5 M $TBAFPF_6$ in acetonitrile at a current of 5 mA. A: Reductive cycling (100 cycles) and B: Oxidative cycling (100 cycles).

2-(2-(2,6-diisopropylphenyl)-1,3-dioxo-2,3-dihydropyrrolo[3,4-*b*]phenothiazin-10(1*H*)-yl)-*N*,*N*,*N*-trimethylethan-1-aminium hexafluorophosphate (***N*-(NMe$_4$)EPhePhtha-1**)

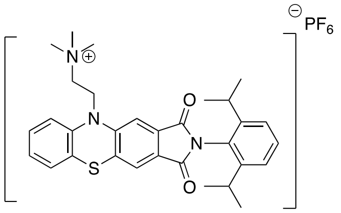

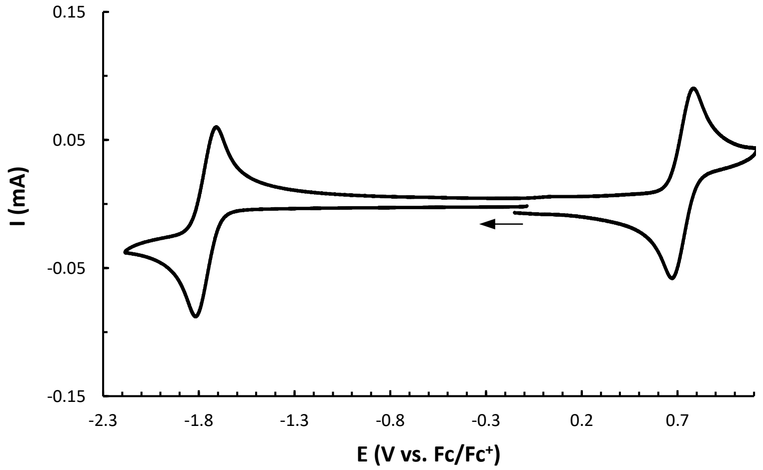

Figure SI-14: Cyclic voltammetry of ***N*-(NMe$_4$)EPhePhtha-1** (5 mM) in 0.5 M TBAFPF$_6$ in acetonitrile at a scan rate of 100 mV/s.

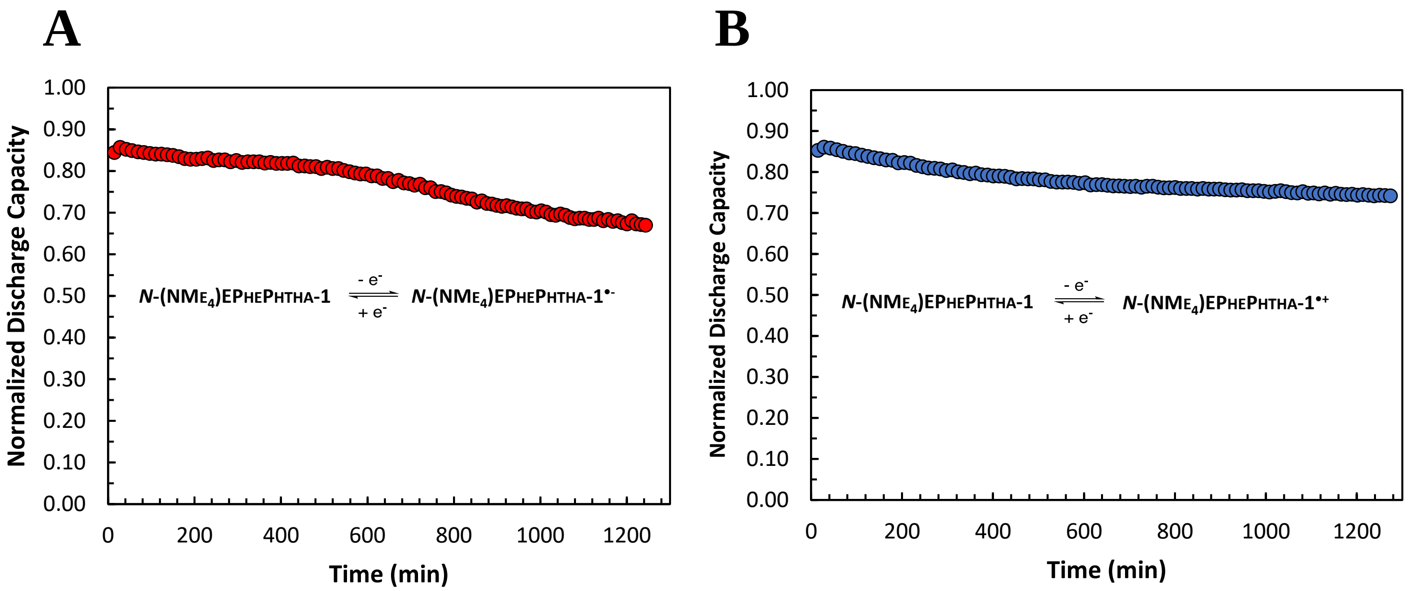

Figure SI-15: H-Cell cycling of ***N*-(NMe$_4$)EPhePhtha-1** (5 mM) in 0.5 M TBAFPF$_6$ in acetonitrile at a current of 5 mA. A: Reductive cycling (100 cycles) and B: Oxidative cycling (100 cycles).

Diffusion coefficient:

Diffusion coefficients for all compounds were calculated from the slopes of peak current densities vs square root of scan rate and the Randles-Ševčík equation (*equation 1*) before being averaged to a single value.

$$i_p = 0.4463nFAC\left(\frac{nFvD}{RT}\right)^{1/2} \qquad (equation\ 1)$$

Where i_p is the peak current (A), n is the number of electrons transferred, A is the active area of the electrode (cm^2), C is the concenrtation in mol/cm^3, F is Faraday's constant (C/mol), ν is scan rate (V/s), D is the diffusion coefficient we are interested in calculating (cm^2/s), R is the ideal gas constant ($JK^{-1}\,mol^{-1}$), and T is temperature (K).

A: *N*-MEEEPhePhtha-1

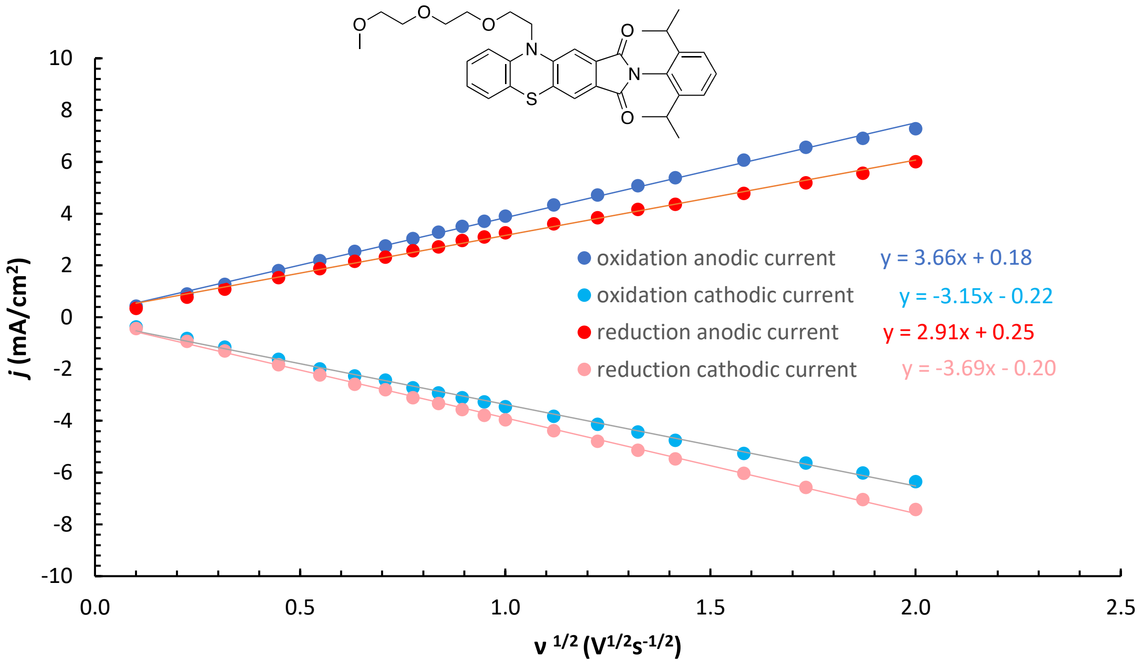

Figure SI-16: Plots of anodic and cathodic peak current densities (j) vs the square root of the sweep rate ($v^{1/2}$) for oxidation (blue) and reduction (red) of ***N*-MEEEPhePtha-1**.

B: *N*-MEEPhePtha-1

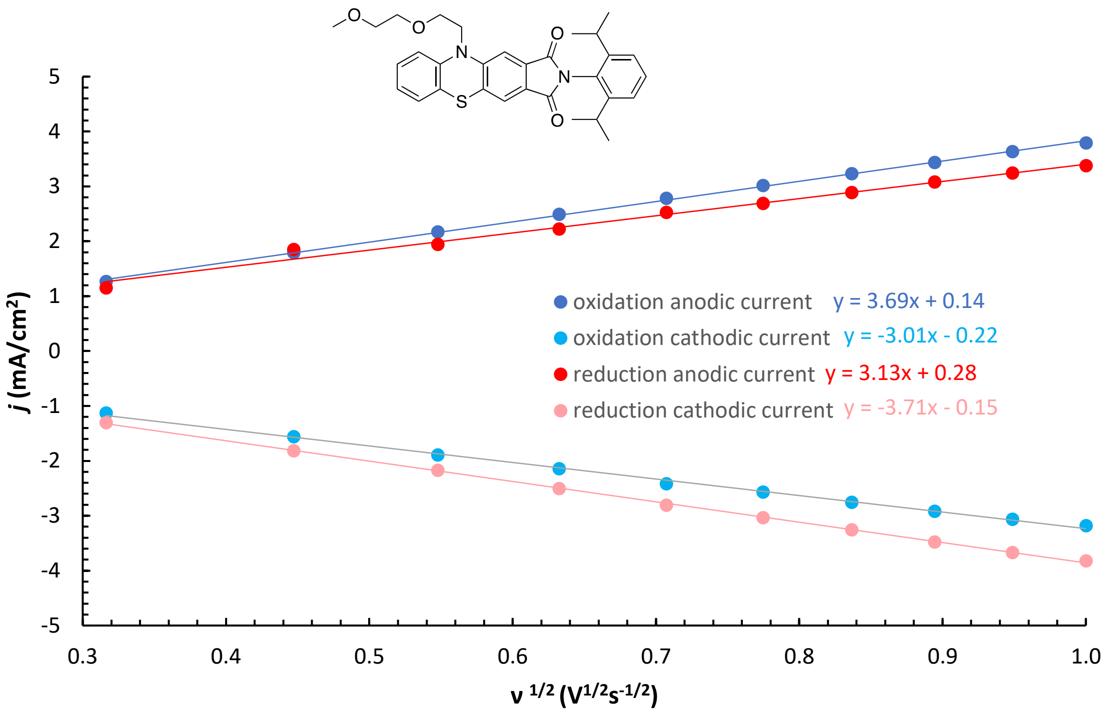

Figure SI-17: Plots of anodic and cathodic peak current densities (j) vs the square root of the sweep rate ($\nu^{1/2}$) for oxidation (blue) and reduction (red) of ***N*-MEEPhePtha-1.**

C: *N*-NMEEPhePhtha-2

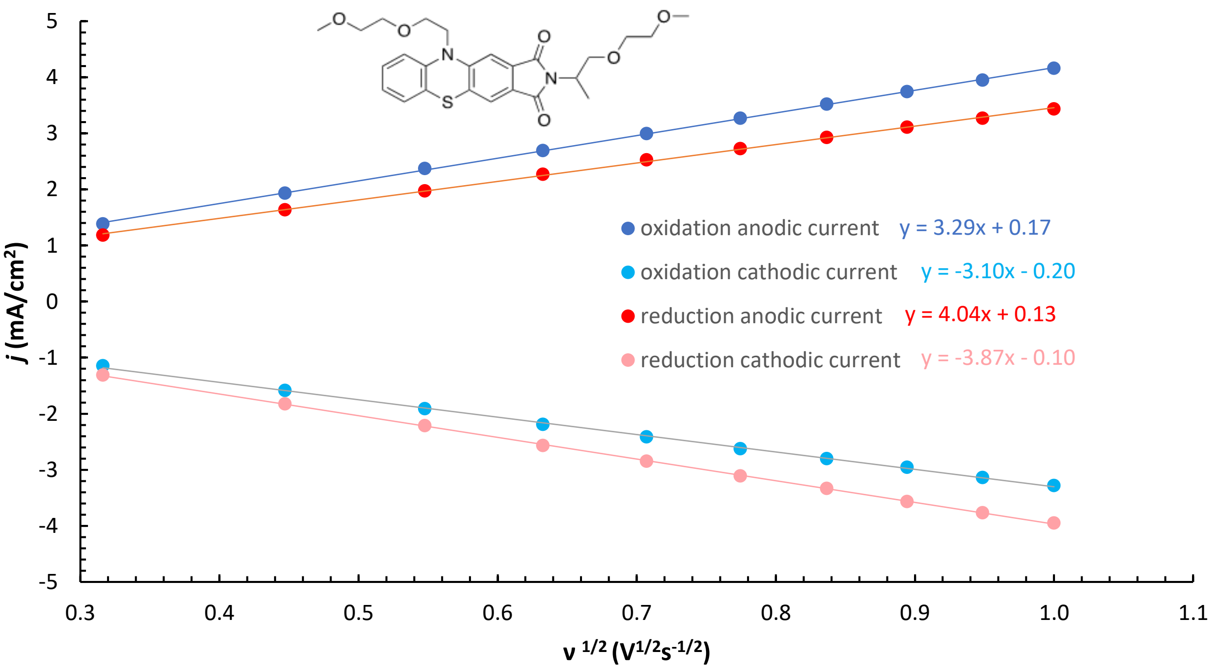

Figure SI-18: Plots of anodic and cathodic peak current densities (j) vs the square root of the sweep rate ($\nu^{1/2}$) for oxidation (blue) and reduction (red) of ***N*-MEEPhePtha-2.**

D: ***N*-MEEE*t*BuPhePhtha-1**

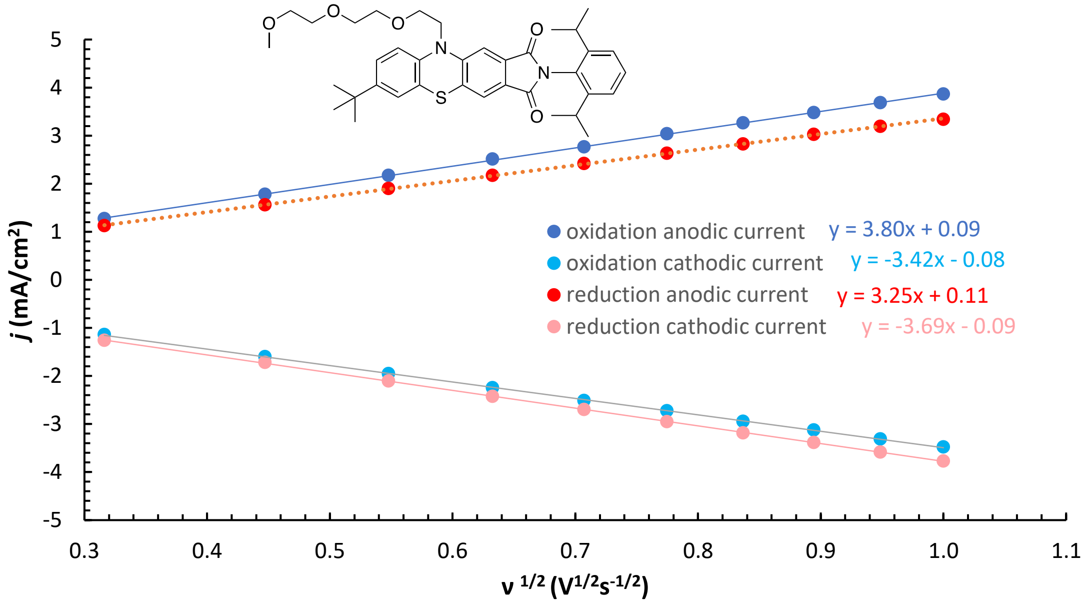

Figure SI-19: Plots of anodic and cathodic peak current densities (j) vs the square root of the sweep rate ($\nu^{0.5}$) for oxidation (blue) and reduction (red) of ***N*-MEEE*t*BuPhePtha-1.**

E: ***N*-(NMe_4)EPhePhtha-1**

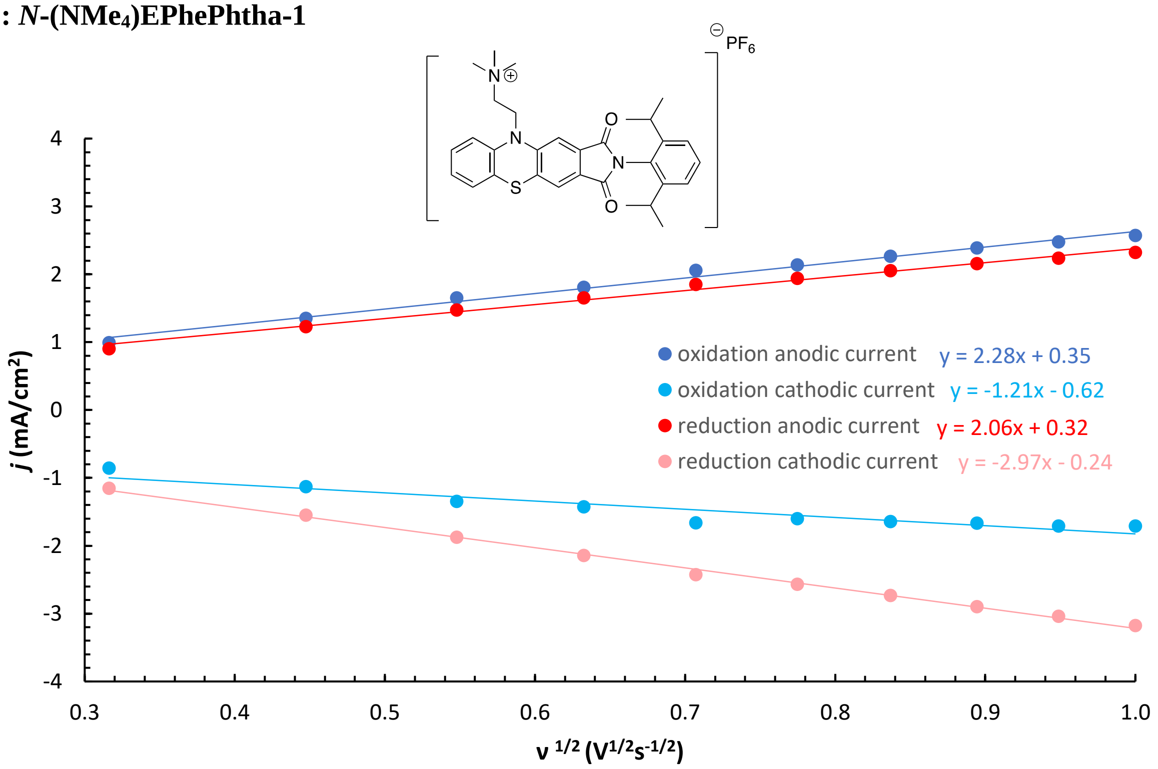

Figure SI-20: Plots of anodic and cathodic peak current densities (j) vs the square root of the sweep rate ($\nu^{1/2}$) for oxidation (blue) and reduction (red) of ***N*-(NMe_4)EPhePtha-1.**

Table SI-1: Averaged diffusion coefficients for the oxidation (D_O) and reduction (D_R) redox couple as well as the overall averaged diffusion coefficient D of compounds A: ***N*-MEEEPhePtha-1**, B: ***N*-MEEPhePtha-1,** C: ***N*-MEEPhePtha-2**, D: ***N*-MEEE*t*BuPhePtha-1,** and E: ***N*-(NMe_4)EPhePtha-1**.

	A	B	C	D	E
D_o ($cm^2\,s^{-1}$)	$6.5 \cdot 10^{-6}$	$6.3 \cdot 10^{-6}$	$7.2 \cdot 10^{-6}$	$7.2 \cdot 10^{-6}$	$1.8 \cdot 10^{-6}$
D_R ($cm^2\,s^{-1}$)	$6.1 \cdot 10^{-6}$	$6.5 \cdot 10^{-6}$	$7.1 \cdot 10^{-6}$	$6.7 \cdot 10^{-6}$	$3.6 \cdot 10^{-6}$
D ($cm^2\,s^{-1}$)	$6.3 \cdot 10^{-6}$	$6.4 \cdot 10^{-6}$	$7.2 \cdot 10^{-6}$	$7.0 \cdot 10^{-6}$	$2.7 \cdot 10^{-6}$

Electron-transfer rate constant k_0:

The standard rate constant k_0 was calculated according to Nicholson's analysis[8] as modified by Magno and coworkers:[9]

$$k_0 = \psi \left(\frac{\pi\, D\, F\, n\, \nu}{R\, T}\right)^{1/2} \qquad (equation\ 2)$$

$$\psi = \frac{(-0.6288 + 0.0021\, \Delta E_p)}{(1 - 0.017\, \Delta E_p)} \qquad (equation\ 3)$$

Where k_0 is the standard rate constant in cm s^{-1}, ψ is the Nicholson dimensionless parameter, π is a mathematical constant, D is the according diffusion coefficient in cm^2 s^{-1}, n is the number of transferred electrons, F is the Faraday constant (96485 C mol^{-1}), ν is the scan rate in V s^{-1}, R is the ideal gas constant (8.3145 J mol K^{-1}), T is the temperature in K and ΔE_p is the peak potential separation at the according scan rate in mV. The electron-transfer rate constant was calculated from the slopes of the Nicholson parameter vs $\nu^{-0.5}$ and equation 2.

A: *N*-MEEEPhePhtha-1

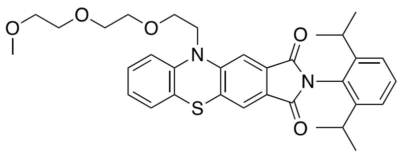

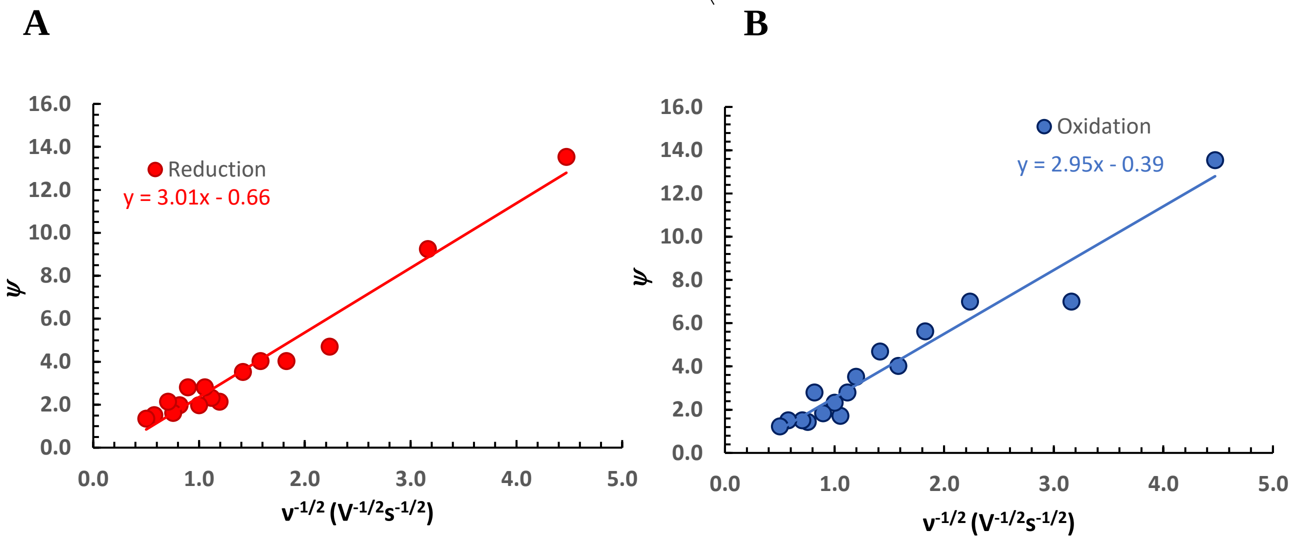

Figure SI-21: Plots of Nicholson parameter ψ vs the $\nu^{-0.5}$ of ***N*-MEEEPhePtha-1** showing scan rates from 50 to 4000 mV/s. **A**: Reduction and **B**: Oxidation.

B: ***N*****-MEEPhePtha-1**

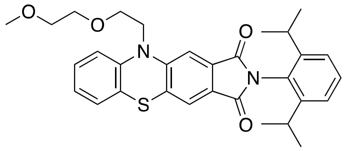

A **B**

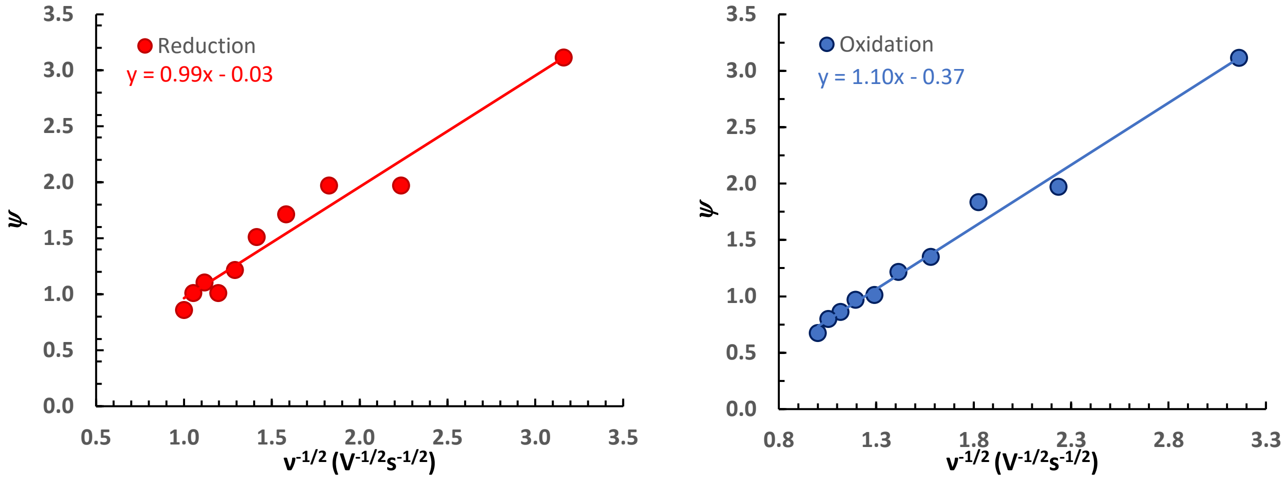

Figure SI-22: Plots of Nicholson parameter ψ vs the $v^{-0.5}$ of ***N*****-MEEPhePtha-1** showing scan rates from 100 to 1000 mV/s. **A**: Reduction and **B**: Oxidation.

C: ***N*****-MEEPhePhtha-2**

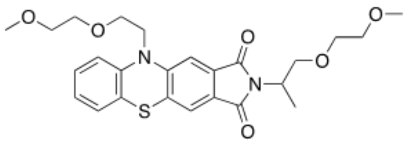

A **B**

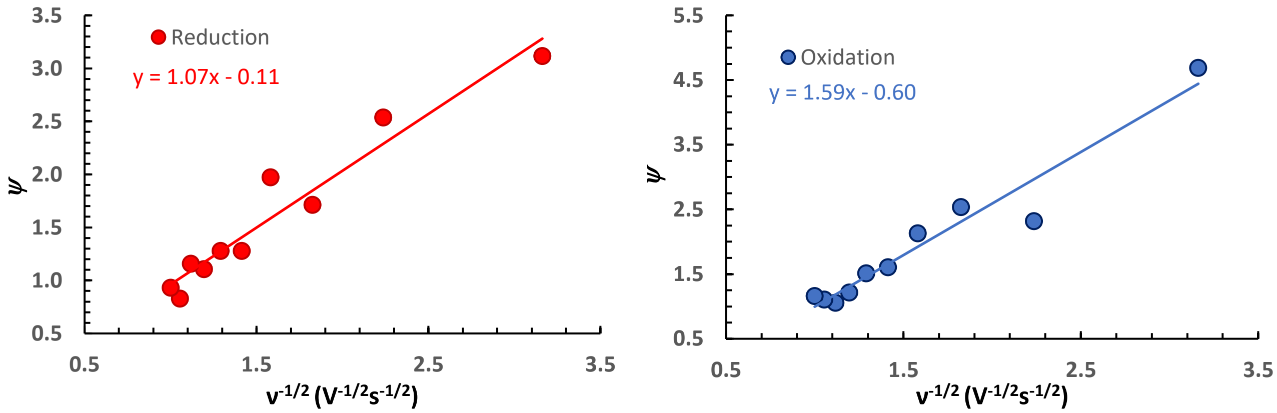

Figure SI-23: Plots of Nicholson parameter ψ vs the $v^{-0.5}$ of ***N*****-MEEPhePtha-2** showing scan rates from 100 to 1000 mV/s. **A**: Reduction and **B**: Oxidation.

D: *N*-MEEE*t*BuPhePhtha-1

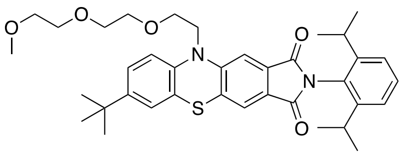

A

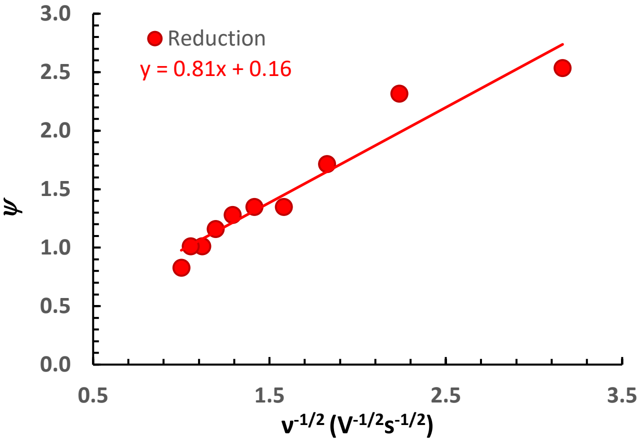

B

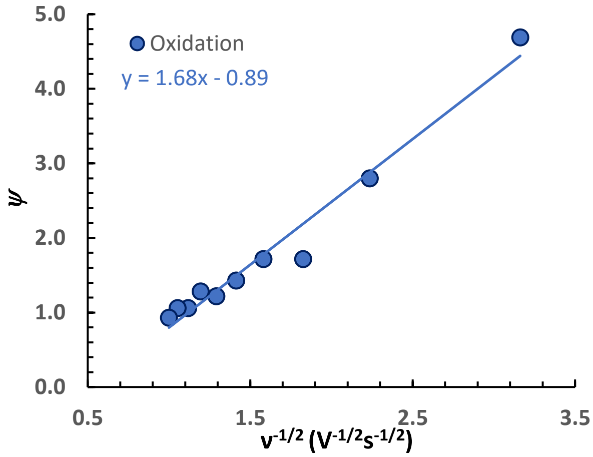

Figure SI-24: Plots of Nicholson parameter ψ vs the $v^{-0.5}$ of ***N*-MEEE*t*BuPhePtha-1** showing scan rates from 100 to 1000 mV/s. **A**: Reduction and **B**: Oxidation.

E: *N*-(NMe$_4$)EPhePhtha-1

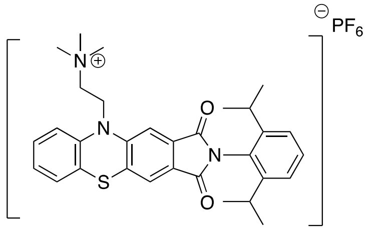

A

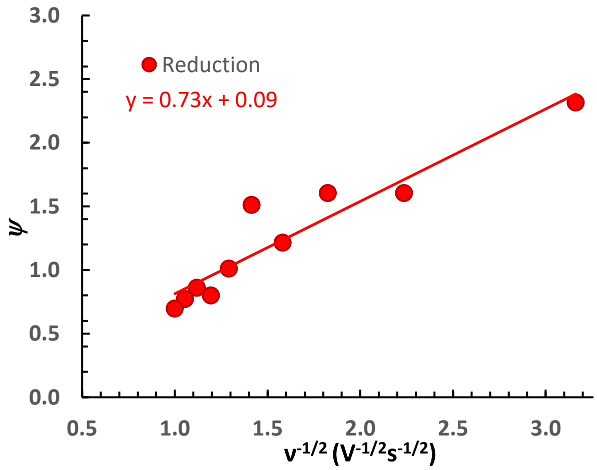

B

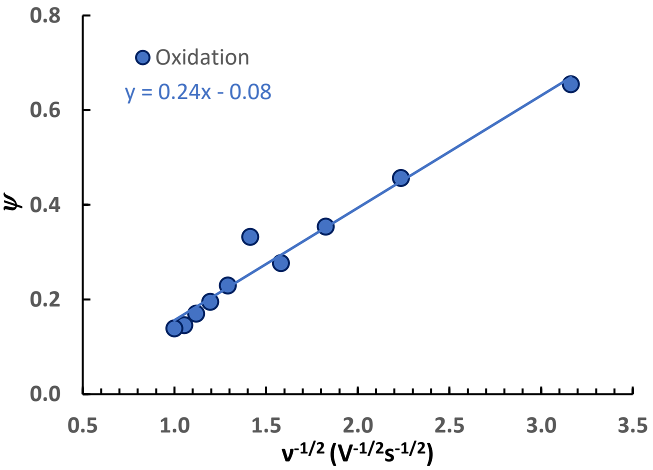

Figure SI-25: Plots of Nicholson parameter ψ vs the $v^{-0.5}$ of ***N*-(NMe$_4$)EPhePhtha-1** showing scan rates from 100 to 1000 mV/s. **A**: Reduction and **B**: Oxidation.

Table SI-2: Averaged standard rate constants k_0 for oxidation and reduction of compounds A: ***N*-MEEEPhePhtha-1**, B: ***N*-MEEPhePhtha-1,** C: ***N*-MEEPhePhtha-2**, D: ***N*-MEEE*t*BuPhePhtha-1,** and E: ***N*-(NMe$_4$)EPhePhtha-1.**

	A	B	C	D	E
Average k_0 **M$^+$ / M** (cm s^{-1})	$8.3 \cdot 10^{-2}$	$3.0 \cdot 10^{-2}$	$4.7 \cdot 10^{-2}$	$5.0 \cdot 10^{-2}$	$3.6 \cdot 10^{-3}$
Average k_0 **M / M$^-$** (cm s^{-1})	$8.2 \cdot 10^{-2}$	$2.8 \cdot 10^{-2}$	$3.2 \cdot 10^{-2}$	$2.3 \cdot 10^{-2}$	$1.5 \cdot 10^{-2}$

Possible insights into capacity loss/increased resistance in flow cycling: Some possible insights into increased cell resistance over time during flow cycling experiments were observed in initial exploratory work with ***N*-MEEEPhePhtha-1** when the flow cell was operated under the following conditions: two stacked Celgard 2400 membranes, 10 mL of 20 mM ***N*-MEEEPhePhtha-1** in 0.5 M TBAPF$_6$/MeCN in each of the catholyte and anolyte reservoirs, and with charging and discharging at constant current densities of 8 mA/cm^2 until reaching voltage cut offs ± 350 mV from the cell's theoretical ΔE^0 of 2.36 V for a total of 17 cycles without any polarity reversals. Under these conditions, (Figure SI-26) efficient charge-discharge cycling was observed over the first 11 cycles followed by a precipitous drop in both charge and discharge capacity until just 21% of the original capacity remained by the end of cycle 17. Despite such a significant drop in cell capacity, post analysis CVs showed little change in the electrochemically active material before and after the flow cycling experiment (Figure SI-27). However, a visual analysis of the two layers of Celgard 2400 membrane after washing with dry and electrolyte-free acetonitrile revealed a significantly larger amount of membrane fouling on the Celgard membrane in direct contact with the anolyte side of the flow battery (labeled A in Figure SI-28) as compared to the Celgard membrane in direct contact with the catholyte side of the flow battery (labeled C in Figure SI-28). This fouling might suggest that the increase in cell resistance observed in the flow cycling experiments could be occurring through a possible interaction of the radical anion with the membrane directly or through a slow chemical reaction following reduction of the neutral molecule to the radical anion (EC mechanism) whose resulting product might subsequently precipitate out of solution and/or directly interact with the membrane. While these explanations are plausible, future investigations are necessary to definitively determine the mechanism of fouling with Celgard 2400 membranes and to determine whether a similar fouling process may or may not be operating with Daramic 175 membranes and when utilizing ***N*-MEEE*t*BuPhePhtha-1**.

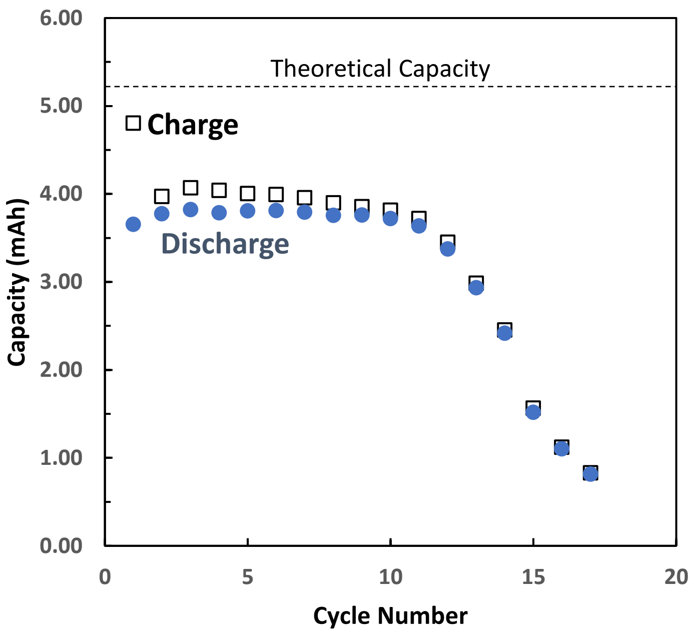

Figure SI-26: Flow cycling data showing charge and discharge capacities for conditions described above. Both cell reservoirs were charged with 10 mL of 25 mM of **MEEEPhePhtha-1** in 0.5 M $TBAPF_6$/MeCN. Charging and discharging were performed at 8 mA/cm2 with voltage cut offs ± 350 mV from the cell's ΔE^0 of 2.36 V.

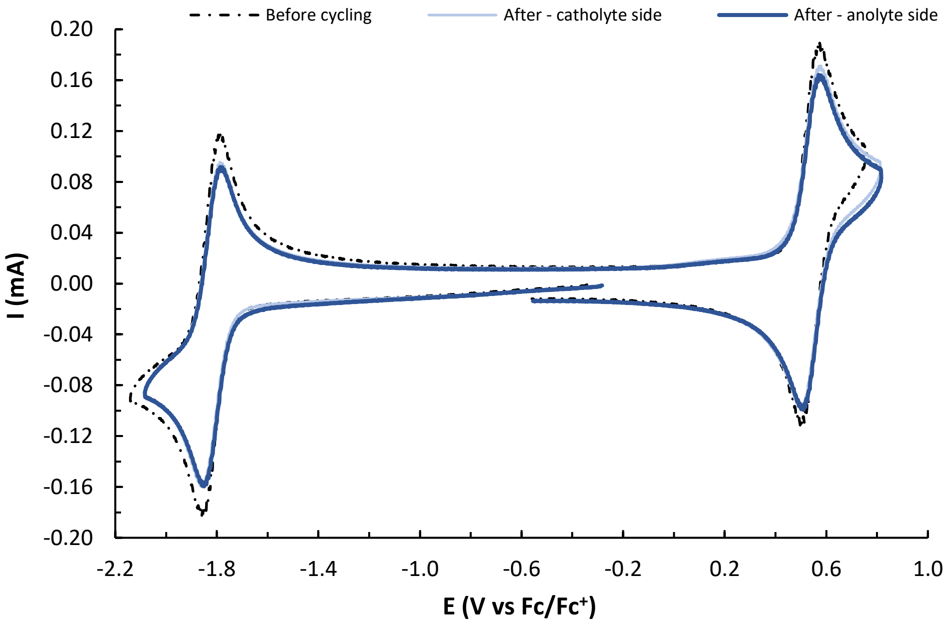

Figure SI-27: CVs (500 mV/s) before and after flow cell cycling of **MEEEPhePhtha-1** as described above. All solutions were diluted in a 1:1 ratio with 0.5 M $TBAPF_6$/MeCN before data acquisition.

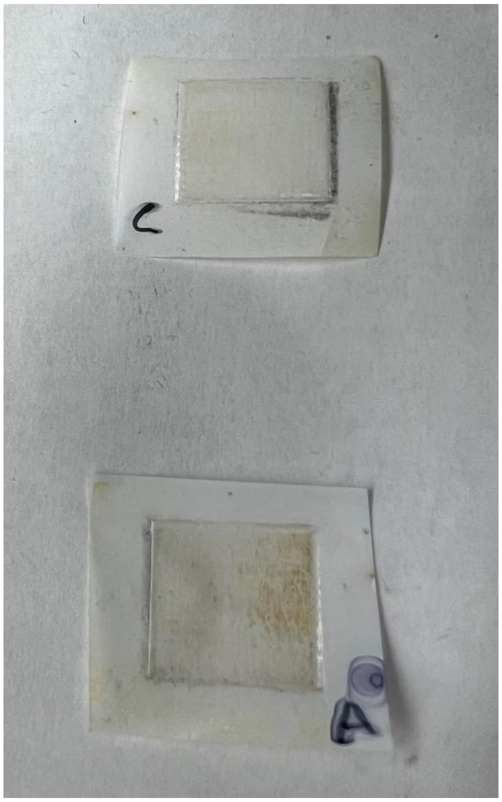

Figure SI-28: Celgard 2400 membranes after flow cell cycling as described above. The membrane labeled "A" was in direct contact with the anolyte side of the flow battery while the membrane labeled "C" was in direct contact with the catholyte side of the flow battery during operation. Membranes were washed with dry acetonitrile and dried before photography.

IV. Computational Details

Initial geometries of neutral, radical cation, and radical anion structures were prepared by hand and subjected to conformational search using the Crest software package, using the GFn2 semiempirical method and GBSA implicit solvent model for acetonitrile.[10] Lowest energy conformers of each were then re-optimized using Gaussian16's implementation of B3LYP/6-31+G* and Truhlar's SMD implicit solvent model for acetonitrile.[11],[12] Frequency calculations were performed at the same level to verify that all structures converged to a minimum. Redox potentials were derived by comparison against the Li/Li+ redox couple calculated with the above computational method. The energy table provides uncorrected Free energies and Free energies (in hartrees) corrected for anharmonicity and concentration (0.025M).[13] NBO calculations on optimized structures were conducted in Gaussian16, at a level of theory identical to that used in the optimization and frequency stages. Molecular orbitals were generated in Gaussian16, using an isovalue of 0.04 and rendered in VMD.

Molecular Orbital Renderings

Neutral ***N*-MEEEPhePhtha-1** HOMO

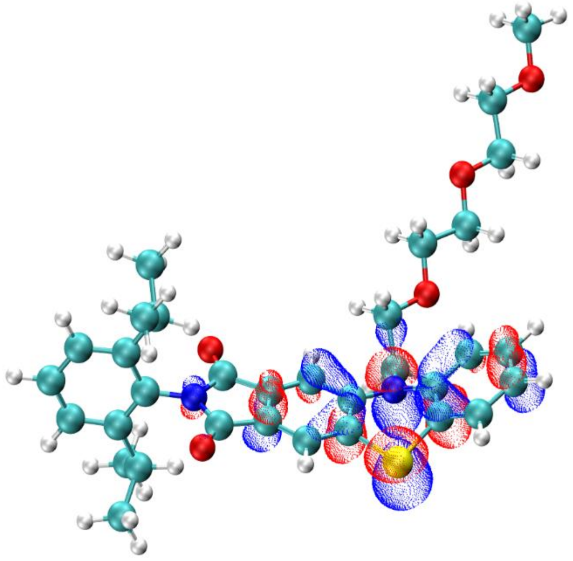

Neutral ***N*-MEEEPhePhtha-1** LUMO

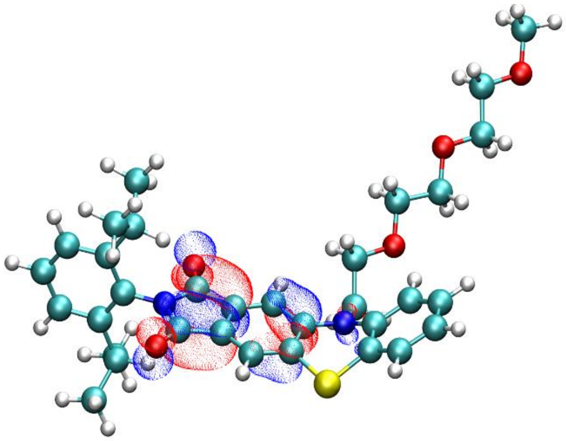

Radical Cation ***N*-MEEEPhePhtha-1** SOMO

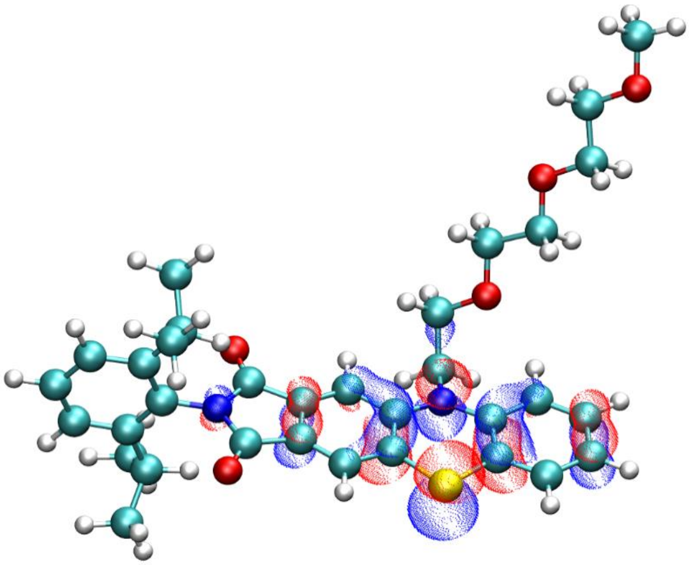

Radical Anion ***N*-MEEEPhePhtha-1** SOMO

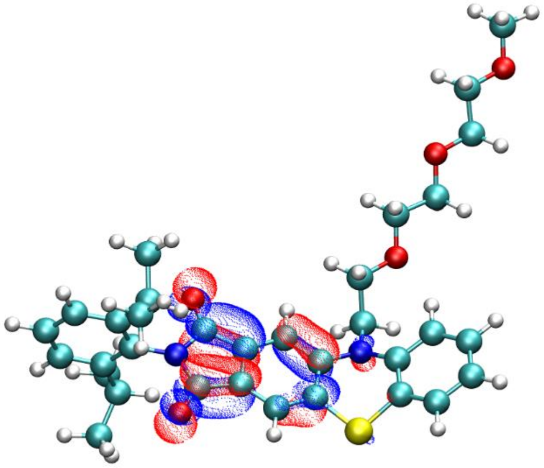

Phthalimide **2** LUMO

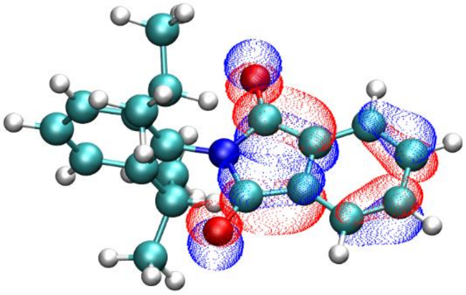

Phthalimide **2** Radical Anion SOMO

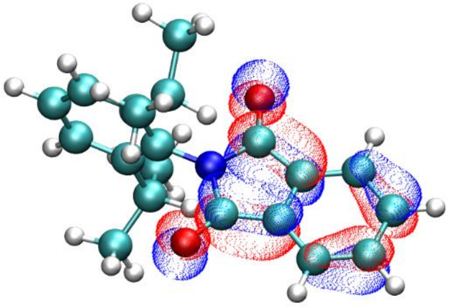

Phenothiazine **1** HOMO

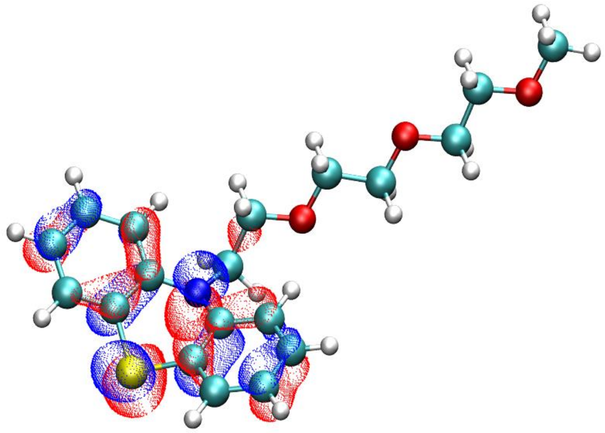

Phenothiazine **1** Radical Cation SOMO

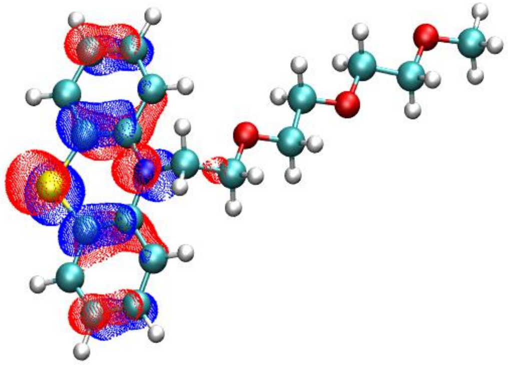

Neutral ***N*-MEEE*t*BuPhePtha-1** HOMO

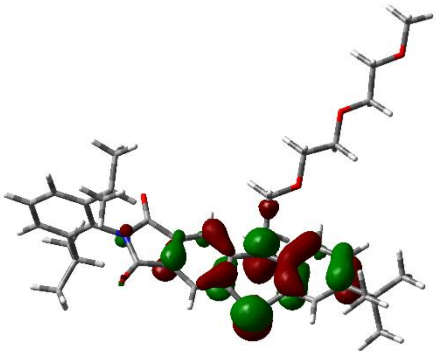

Neutral ***N*-MEEE*t*BuPhePtha-1** LUMO

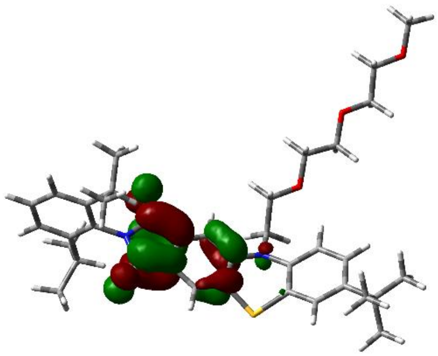

Radical Cation ***N*-MEEE*t*BuPhePtha-1** SOMO

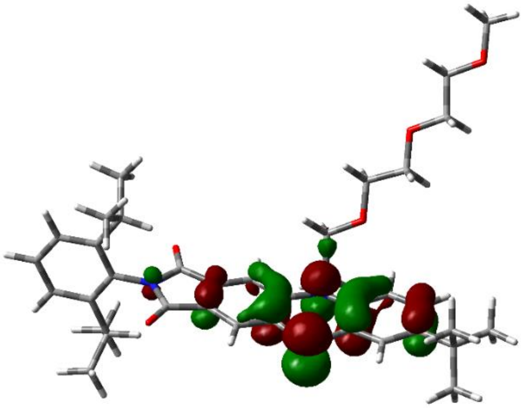

Radical Anion ***N*-MEEE*t*BuPhePtha-1** SOMO

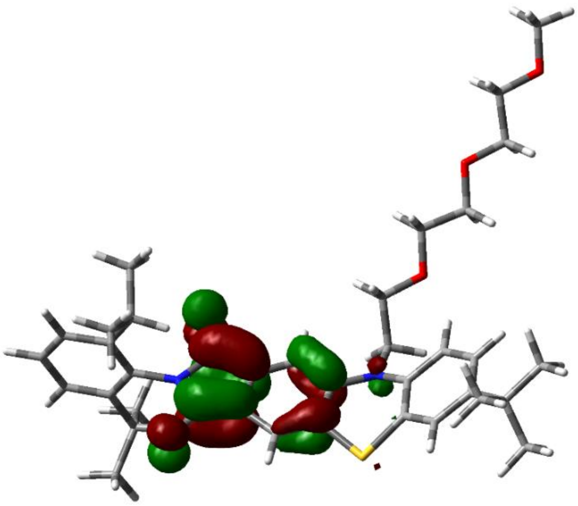

Energy Table

Structure	ΔE	ΔZPE	ΔH	TΔS	TΔS$_{(qh)}$	ΔG(T)	ΔG(T)$_{(qh)}$
Neutral **N-MEEEPhePhtha-1**	-2164.31954	0.655121	-2163.622697	0.121829	0.110529	-2163.744526	-2163.733225
Radical Anion **N-MEEEPhePhtha-1**	-2164.421264	0.652532	-2163.726905	0.122044	0.111065	-2163.848949	-2163.83797
Radical Cation **N-MEEEPhePhtha-1**	-2164.13489	0.657063	-2163.436475	0.120668	0.110015	-2163.557143	-2163.54649
Li+	-7.424485	0	-7.422125	0.015572	0.015572	-7.437697	-7.437697
Li	-7.49278	0	-7.49042	0.016227	0.016227	-7.506646	-7.506646
Phthalimide **2** Neutral	-980.086381	0.365127	-979.699017	0.073524	0.069741	-979.772541	-979.768758
Phthalimide **2** Anion Radical	-980.188964	0.362125	-979.804403	0.074796	0.070764	-979.879199	-979.875167
Phthalimide **2** Cation Radical	-979.856011	0.36387	-979.469425	0.075773	0.071695	-979.545197	-979.54112
Phenothiazine **1** Neutral	-1416.50018	0.391211	-1416.084608	0.082092	0.076233	-1416.1667	-1416.160841
Phenothiazine **1** Anion Radical	-1416.546639	0.386594	-1416.135085	0.083442	0.077864	-1416.218527	-1416.212949
Phenothiazine **1** Cation Radical	-1416.324433	0.392367	-1415.907755	0.083349	0.077006	-1415.991104	-1415.984761
Neutral ***N*-MEEE*t*BuPhePtha-1**	-2321.579308	0.767848	-2320.764228	0.133016	0.120691	-2320.897245	-2320.884919
Radical Anion ***N*-MEEE*t*BuPhePtha-1**	-2321.680458	0.764885	-2320.868037	0.135801	0.122468	-2321.003838	-2320.990505
Radical Cation ***N*-MEEE*t*BuPhePtha-1**	-2321.397135	0.768674	-2320.58125	0.1339	0.121282	-2320.715149	-2320.702532

Redox Properties

Reaction	ΔΔG (kcal/mol)$_{corr}$	E
Neutral **N-MEEEPhePhtha-1** + Li → Radical Anion **N-MEEEPhePhtha-1** + Li^+	-22.5	-0.97
**N-MEEEPhePhtha-1** Radical Cation + Li → Neutral **N-MEEEPhePhtha-1** + Li^+	-73.9	-3.21
Neutral Phthalimide **2** + Li → Radical Anion **2** + Li^+	-23.5	-1.02
Phenothiazine Radical Cation **1** + Li → Neutral **1** + Li^+	-67.2	-2.92
***N*-MEEE*t*BuPhePtha-1**+ Li → Radical Anion ***N*-MEEE*t*BuPhePtha-1** + Li^+	-23.0	-1.00
***N*-MEEE*t*BuPhePtha-1** Radical Cation + Li → Neutral ***N*-MEEE*t*BuPhePtha-1** + Li^+	-71.2	-3.09

Structure XYZ Files

phth.out

C	0.7741886127	2.9638792952	0.021421369
C	-0.0580809117	1.9499281948	-0.47369555
C	0.4220159748	0.6440488659	-0.6588230335
H	-0.2225080416	-0.1416677184	-1.0423948586
C	1.7498976089	0.399730828	-0.3324452504
C	2.5797618038	1.4107761509	0.1612926263
C	2.1146779107	2.7062415527	0.3482116378
H	2.759664698	3.4915458568	0.7318368354
C	2.546127429	-0.8563287303	-0.4130038528
O	2.1927512112	-1.9606009311	-0.7910987874
C	3.9270306398	0.826036087	0.4086451589
O	4.9290983421	1.373313287	0.8371875829
N	3.8380403444	-0.5316261257	0.0446313669
C	4.9285603198	-1.4681759073	0.1294293627
C	5.7813847616	-1.6219364364	-0.9858610847
C	5.1059935395	-2.1991881128	1.3248272435
C	6.8353703485	-2.5406095702	-0.8760734224
C	6.1758155914	-3.1043493019	1.3801799014
C	7.0324940158	-3.2749541046	0.293024659
H	7.5093908709	-2.6820386415	-1.7167692944
H	6.3393060248	-3.6821374161	2.2859987089
H	7.8561823139	-3.9822931386	0.3570731928
C	4.1897255928	-2.0396825292	2.5351913091
H	3.4266495687	-1.2940630955	2.2914650844
C	4.9602360728	-1.5223435826	3.7663277296
H	5.71469016	-2.2461412838	4.0986775475
H	5.4698976015	-0.5751290852	3.5532341207
H	4.26693188	-1.354183962	4.6001693306
C	3.456806755	-3.3541942803	2.8696940645
H	2.8844587188	-3.7254715673	2.0112419283
H	4.161444057	-4.1386657446	3.1723954914
H	2.7571988704	-3.1936525446	3.6997623031
C	5.5973930452	-0.8366295942	-2.2817792755
H	4.7290596869	-0.1809222234	-2.1647991404
C	6.8144482196	0.0619467543	-2.5789531423
H	7.7177288054	-0.534712688	-2.7567642089
H	6.6273580135	0.6627902919	-3.4779646373
H	7.0197366861	0.74872891	-1.7491577778
C	5.3104898791	-1.7696899059	-3.4751535546
H	6.1639491601	-2.427060819	-3.6825473869
H	4.4332610831	-2.4011281809	-3.2904102908
H	5.1171914431	-1.1765349503	-4.3779514298
H	-1.091640242	2.1791650331	-0.7180133126
H	0.3745533948	3.9654526244	0.1542439568

phth_an.out

C	0.7966612748	2.9622404371	0.0226813488
C	-0.053226505	1.9276855588	-0.4726693375
C	0.4261717226	0.6351656345	-0.6500797965
H	-0.2261545693	-0.1497309246	-1.0282460471
C	1.7700718773	0.3565765146	-0.3324167467
C	2.6249934787	1.3973994343	0.1664328939
C	2.1245667048	2.7026581153	0.3407338635
H	2.7681127963	3.4950593396	0.7182863369
C	2.5310072612	-0.8668718362	-0.4124602691
O	2.1961016118	-2.0167978594	-0.7892120408
C	3.9350908243	0.8425028774	0.4075317062
O	4.9848676065	1.3786712628	0.8399266309
N	3.8322299848	-0.5265935723	0.0441507015
C	4.9166333751	-1.4578191938	0.1284855192
C	5.7768055666	-1.618879242	-0.9821684639
C	5.1027483993	-2.1952555315	1.3205155513
C	6.832115884	-2.5371142756	-0.8750152191
C	6.1720457927	-3.1016480966	1.3785786354
C	7.0309337474	-3.2735743261	0.2929035958
H	7.5062881896	-2.6773401942	-1.7164504278
H	6.3342764162	-3.6797128483	2.2850590838
H	7.8548089425	-3.9811305192	0.3569672789
C	4.1852454505	-2.0347754702	2.5299963873
H	3.4272767951	-1.2869473531	2.2781330069
C	4.951611102	-1.5195366666	3.7646269563
H	5.7036144629	-2.2447268939	4.1005708389
H	5.4645457721	-0.573554561	3.5540300786
H	4.2564419195	-1.3492064916	4.5968616963
C	3.4479864228	-3.3465367283	2.8658672893
H	2.8768158911	-3.7182296997	2.0068862948
H	4.149575041	-4.133007981	3.1716885603
H	2.7461059872	-3.1842673288	3.6940727812
C	5.591685016	-0.8324147239	-2.2773942273
H	4.7214426773	-0.1811517552	-2.1519610252
C	6.8057751827	0.0694805708	-2.5767565224
H	7.7106197441	-0.524408596	-2.7579671287
H	6.6165102503	0.6724973472	-3.4742198077
H	7.0120059852	0.7551494062	-1.7463494281
C	5.3060887963	-1.7613085091	-3.4743597321
H	6.1606301938	-2.4166474651	-3.6854608001
H	4.4305731647	-2.3956488847	-3.2915720047
H	5.1103571616	-1.1665241757	-4.3759188208
H	-1.0884999447	2.1588261562	-0.7133206289
H	0.3951744064	3.9649206086	0.1515146585

phth_cat.out

C	0.7448325582	2.9913755518	0.014762368
C	-0.0900052251	1.975286285	-0.4716652716
C	0.387817952	0.6682426644	-0.65279196
H	-0.2585457189	-0.1188328147	-1.02982531
C	1.7171895217	0.4250519344	-0.3308110472
C	2.5503011735	1.43902538	0.1547024499
C	2.0865278043	2.7357170855	0.3373514219
H	2.7334366447	3.5225657176	0.714018675
C	2.504502399	-0.8331996676	-0.4111283704
O	2.1604515826	-1.94123569	-0.7788064097
C	3.8984390712	0.8631134524	0.4011769393
O	4.9064594828	1.4012053196	0.8205143142
N	3.8045266416	-0.5027988806	0.0421527487
C	4.8853763486	-1.4312869075	0.1260556935
C	5.7443172939	-1.5967636221	-0.9755680494
C	5.0764965469	-2.1675134963	1.3093253137
C	6.8355954729	-2.5487440371	-0.8615664187
C	6.1845191058	-3.1051877892	1.3661113357
C	7.0449189434	-3.2863145628	0.2938487705
H	7.4982472284	-2.6771117359	-1.7107709741
H	6.3321712943	-3.6737894648	2.2780391462
H	7.8664091781	-3.9921513902	0.3576369957
C	4.1955927822	-2.0371129471	2.518538512
H	3.4219005109	-1.2959529226	2.3066317954
C	5.006486868	-1.5430652257	3.743108096
H	5.7649528161	-2.2711227365	4.0516799714
H	5.5009474997	-0.5881383248	3.5369176827
H	4.3123262937	-1.400755425	4.5792980579
C	3.493875894	-3.38142412	2.8381909937
H	2.9084466917	-3.7402038102	1.9853417236

H	4.2114589858	-4.1590187702	3.1227728784
H	2.8139927286	-3.2209990327	3.6827947072
C	5.5943442206	-0.8414119225	-2.264796732
H	4.7312445188	-0.1775258282	-2.1803139727
C	6.8435199905	0.0327798999	-2.5418858016
H	7.7407242616	-0.5757055709	-2.7010602454
H	6.6605751364	0.6131639003	-3.4534794638
H	7.0345722565	0.7300730403	-1.7196302315
C	5.3364114612	-1.8102843327	-3.4464382415
H	6.192906886	-2.4686653809	-3.6294685769
H	4.4502291736	-2.4288961801	-3.2707482213
H	5.168548382	-1.2124092684	-4.3496029897
H	-1.1243532008	2.2039891933	-0.7125332291
H	0.346042403	3.9935039927	0.1444241462

thia.out

C	-4.1802997149	4.0398251415	1.0538374974
C	-3.5061036891	3.0641576402	0.3126684257
C	-2.1380192051	3.2033324566	0.0116280202
C	-1.4714683883	4.3516396948	0.4916663172
C	-2.1404365624	5.3026176607	1.2665320808
C	-3.5037685476	5.1591536952	1.5419065431
C	0.7902641062	2.9648378784	-0.044444308
C	-0.0897108467	1.9464949664	-0.471180289
C	0.4320436174	0.6467758609	-0.6098150889
H	-0.2072910298	-0.1692504581	-0.9260163442
C	1.7793701617	0.3795689222	-0.3481159797
C	2.6328465965	1.3923983961	0.0929202415
C	2.1257187592	2.6850967023	0.2563923439
H	-5.2397827574	3.9071593323	1.2581645957
H	-4.0565569605	2.1965137794	-0.0326418802
H	-1.5961646981	6.1693858733	1.6336196817
H	-4.0227123276	5.9118485063	2.1290070806
H	2.7742836597	3.4885643136	0.5970486344
N	-1.4419091922	2.2430190122	-0.7599641432
S	0.2163735372	4.6529964002	-0.001124157
C	-2.2140864594	1.2893482439	-1.5643376806
H	-1.5660631846	0.9578217596	-2.3794835149
H	-3.041151117	1.8396043951	-2.021741047
C	-2.802303543	0.0711419991	-0.8158867779
H	-2.3385251525	-0.8650880002	-1.158938898
H	-2.6269802786	0.1552844765	0.2668720245
C	-4.8410443973	-1.090876575	-0.4579812711
H	-4.3863455543	-2.0330266951	-0.800700047
H	-4.7272506376	-1.0321670829	0.6355112268
C	-6.3138569613	-1.0506576237	-0.8367951858
H	-6.4264130501	-1.1175073149	-1.9299554659
H	-6.7674614051	-0.1052409793	-0.5013613368
O	-4.2039399478	0.0194723976	-1.0805208583
O	-6.951108492	-2.155161642	-0.2061303068
C	-8.3406484851	-2.2345461059	-0.5026896344
H	-8.4934391993	-2.3468578849	-1.5872376932
H	-8.8537283808	-1.3166889473	-0.1764841263
C	-8.9039790304	-3.4401704019	0.2342297783
H	-8.7546592069	-3.3256376026	1.3195225332
H	-8.3862277581	-4.3575392809	-0.0877336264
C	-10.9231581511	-4.6217876693	0.5774921724
H	-11.9769085178	-4.6034874059	0.2852248901
H	-10.4774067097	-5.578245848	0.2642634141
H	-10.8494316253	-4.5365629916	1.6724189984
O	-10.2906358272	-3.5237329768	-0.0675433699
H	2.1507778192	-0.6337270467	-0.4780805571
H	3.6771644349	1.1855161786	0.3100092278

thia_an.out

C	-3.3839681635	5.3902410507	1.271242089
C	-3.152873243	4.1401521736	0.6979876272
C	-1.8680497194	3.7521491983	0.2349703013
C	-0.8213796758	4.7164219026	0.3639241533
C	-1.0628645556	5.9517472668	0.9742760361
C	-2.3370915661	6.3091418342	1.4292088458
C	0.7221900403	2.5314500011	0.1013245401
C	-0.4300167677	1.8345604328	-0.4265076229
C	-0.286507062	0.5245127126	-0.8901530407
H	-1.1400065489	-0.0325370263	-1.2649226346
C	0.9616558304	-0.1448008725	-0.8402405446
C	2.0873430004	0.5222864257	-0.3037063733
C	1.9665918057	1.8352786634	0.1399738865
H	-4.3872571156	5.6346989697	1.6135436136
H	-3.9878579236	3.4535676885	0.6240932208
H	-0.2333177954	6.6486057042	1.0767763412
H	-2.5051963243	7.2778290843	1.8929041086
H	2.8436693007	2.3576682482	0.5262922179
N	-1.6790737302	2.5088871576	-0.4070466428
S	0.7749001465	4.356056398	-0.3101019209
C	-2.8476073588	1.8326211339	-0.9779563857
H	-2.5377383924	1.3161031613	-1.8916442107
H	-3.5771096022	2.5878897183	-1.2809771192
C	-3.5160575857	0.8217487193	-0.0383892781
H	-2.793438743	0.0685017744	0.3050365349
H	-3.927833505	1.3183193276	0.8521477243
C	-5.2728204743	-0.7738523242	-0.0035885452
H	-4.5870384954	-1.5600151729	0.3484362996
H	-5.7334042148	-0.3006944824	0.8775561166
C	-6.3471461809	-1.3768985803	-0.8961755402
H	-5.8867018438	-1.8531007594	-1.7755839044
H	-7.0323846115	-0.5911161241	-1.2501430703
O	-4.5646951096	0.1924805332	-0.7706400747
O	-7.0567532548	-2.34130103	-0.127329031
C	-8.0906455848	-2.9839683598	-0.8638709403
H	-7.670157799	-3.5019522552	-1.7397656875
H	-8.8222570575	-2.242884949	-1.2214177711
C	-8.7665763856	-3.9839228087	0.0620612424
H	-9.1878649109	-3.4656413945	0.937974481
H	-8.0336341509	-4.7233929674	0.4215630292
C	-10.5008570433	-5.5908333126	0.1021817221
H	-11.2662848419	-6.0292113942	-0.5444373383
H	-9.827048112	-6.3861348589	0.4559248144
H	-10.9865191943	-5.123176894	0.9722833752
O	-9.7981915168	-4.628576521	-0.673801537
H	1.0348163807	-1.1682929431	-1.1999966766
H	3.0429253555	0.0050008999	-0.2192542911

thia_cat.out

C	-4.5284997641	4.1275097798	-0.0261094439
C	-3.7096585318	3.1249624724	-0.5136681405
C	-2.3408180702	3.0515045618	-0.1498360546
C	-1.8605697289	4.032546401	0.7643684156
C	-2.7048590311	5.0504514161	1.2458834748
C	-4.0295869023	5.1072627001	0.8512275305
C	0.4508515499	2.6073145909	0.7088542834
C	-0.2270754018	1.775023015	-0.2269639976
C	0.4722471182	0.6294701175	-0.689523305
H	0.0145299874	-0.0576607419	-1.3858393891
C	1.7511715974	0.3402209232	-0.2477325685

C	2.4044466097	1.1751601198	0.6759519954
C	1.7524809895	2.300457894	1.1471175437
H	-5.5732273762	4.14356873	-0.3220634475
H	-4.1435864559	2.364438792	-1.1452130107
H	-2.3060910105	5.7894061009	1.9361712278
H	-4.676718291	5.8932543457	1.2281961201
H	2.2402669363	2.9595178407	1.860656628
N	-1.5164485776	2.0591331712	-0.6890004518
S	-0.2173103462	4.0874411056	1.334257771
C	-2.0007329467	1.2996905466	-1.877851327
H	-1.1388519285	1.1464410055	-2.526545676
H	-2.6825599457	1.9429037502	-2.4314736928
C	-2.6518878464	-0.053637491	-1.5915994301
H	-2.6768587077	-0.6016829171	-2.5459733697
H	-2.0597244801	-0.6408502677	-0.8771743905
C	-4.6397454038	-1.1233755262	-0.8846597145
H	-4.6877731571	-1.6900732858	-1.8266373233
H	-4.0933332913	-1.7269135445	-0.1446749885
C	-6.0431972538	-0.8158075589	-0.3854210498
H	-6.5787475255	-0.195023234	-1.1201116832
H	-5.9986672268	-0.2635183258	0.5658265068
O	-3.9717536642	0.1189815856	-1.095663536
O	-6.7129806838	-2.0574786923	-0.2064817248
C	-8.0559337962	-1.9025692718	0.2391183177
H	-8.6302584093	-1.3026922035	-0.4835793916
H	-8.0787302613	-1.3856483762	1.2108585195
C	-8.6640614838	-3.2909952796	0.3673724025
H	-8.092049514	-3.8912035918	1.0925428506
H	-8.6343226518	-3.8083130234	-0.6045971374
C	-10.6786746938	-4.3821462072	0.9511893588
H	-11.6968981171	-4.1621075644	1.2841605764
H	-10.720063556	-4.92708923	-0.00426175
H	-10.1814392693	-5.0160916934	1.7011091727
O	-10.0079879569	-3.136884663	0.8040058154
H	2.2461755851	-0.550947779	-0.6217481535
H	3.4073785849	0.941664653	1.0195677775

neutralX.out

C	-0.2753216717	-4.9300605345	-0.9914721412
C	1.1090456681	-4.808313605	-0.8347535901
C	1.781005652	-3.6522051749	-1.2685096518
C	1.0165635109	-2.6197432267	-1.8462992515
C	-0.3708035376	-2.7289061787	-1.9659955957
C	-1.0235981146	-3.8933780751	-1.5511682883
C	3.1926363201	-1.0806090153	-1.3784898403
C	3.7655550357	-2.2736346198	-0.8574311074
C	4.9399981312	-2.1716535432	-0.0757765315
H	5.4173495199	-3.0468702857	0.3470041883
C	5.4797978596	-0.9164911443	0.1481462729
C	4.8900844616	0.2477208026	-0.3422749622
C	3.7292844819	0.181890019	-1.1029352666
H	-0.7647856741	-5.8412795834	-0.6574254976
H	1.6591101769	-5.6210193046	-0.3740116779
H	-0.9356575949	-1.9101251408	-2.4045582075
H	-2.1005070936	-3.9821130072	-1.6642682513
H	3.2601392204	1.0770860714	-1.5016699859
C	6.6984413627	-0.5329072067	0.9138870726
O	7.4917178725	-1.2551644282	1.4953083778
C	5.704337441	1.4056950224	0.0932457979
O	5.5300195305	2.594471514	-0.121278995
N	6.7767678045	0.8691851013	0.8419042031
N	3.1888476787	-3.5193675786	-1.1365079327
S	1.843076231	-1.1952736843	-2.5310485777
C	7.8156502954	1.654789315	1.453881252
C	8.9793097286	1.9440824688	0.7074683825
C	7.6368597081	2.1045921361	2.7806962285
C	9.9792209223	2.7049529188	1.3304433943
C	8.6683122535	2.861200495	3.3556333636
C	9.8278456483	3.1594664593	2.6402783746
H	10.8868713986	2.9433636893	0.782412495
H	8.561147677	3.2203651576	4.3757296906
H	10.6158629355	3.7477624098	3.1048967403
C	6.3816211506	1.7984936564	3.5933861603
H	5.7033073143	1.211099732	2.9667648426
C	5.6359492264	3.0869671054	3.9939800107
H	6.241245649	3.7072344447	4.6665574036
H	5.3779100096	3.6913669388	3.1161793764
H	4.7050561843	2.83504391	4.517633304
C	6.7054501782	0.9506270135	4.8396192942
H	7.2153281738	0.018147392	4.5697659141
H	7.3485291996	1.4984536454	5.539507424
H	5.7798910307	0.6900133208	5.3684581274
C	9.1805099515	1.4636666552	-0.7273093526
H	8.2914328904	0.9003744918	-1.0275083661
C	9.3280415703	2.6449419601	-1.7067919001
H	10.2326262275	3.2287491024	-1.4960507054
H	9.4022208123	2.2722340834	-2.736277431
H	8.4673671434	3.3221858119	-1.6528992934
C	10.3874830041	0.5114757989	-0.8427606451
H	11.326374261	1.0254831684	-0.6023379637
H	10.2884917424	-0.3468039475	-0.1675613929
H	10.4679418811	0.1287147592	-1.8680790286
C	3.9971605911	-4.7311007103	-0.9316212727
H	4.9976122507	-4.5153566162	-1.3134617038
H	3.5789083014	-5.5142819646	-1.5690095507
C	4.0769799548	-5.267229534	0.5155713414
H	5.1176440944	-5.2704428511	0.871168359
H	3.4896758381	-4.6367530016	1.1993450248
C	3.6239033749	-7.19287273	1.826039917
H	4.6623757433	-7.2265699452	2.1893420942
H	3.0293480856	-6.5999296787	2.537835181
C	3.0592115317	-8.60108541	1.7140913208
H	3.6540601279	-9.1942380569	1.0024221255
H	2.020880542	-8.5665778231	1.3498783994
O	3.5741756563	-6.6022735721	0.5311114366
O	3.1091292404	-9.1877938971	3.0089703014
C	2.6035196474	-10.5178452642	3.0339202215
H	3.1861748572	-11.1603700843	2.3558694126
H	1.5530685055	-10.5330839606	2.7044987646
C	2.7122956025	-11.0274698216	4.4630912926
H	2.131834395	-10.3819604233	5.1410393638
H	3.7633981765	-11.0124256945	4.7921114234
C	2.2583821882	-12.9309912762	5.7897894855
H	1.8496745688	-13.9423614064	5.7109961323
H	3.2940832732	-12.986863054	6.1582272937
H	1.6565612861	-12.3519117464	6.5067346015
O	2.2049797263	-12.3551887636	4.4907027743

anionX.out

C	-0.3468470556	-4.8252562682	-0.986540093
C	1.0399473966	-4.7359233143	-0.832087398
C	1.7464246496	-3.5977891768	-1.2704287783
C	1.0023425363	-2.5493565325	-1.8599996865
C	-0.3878767933	-2.6309442007	-1.9783801915
C	-1.0716567005	-3.7739267704	-1.5525818137
C	3.2153288047	-1.0487644039	-1.419144562

C	3.7754419511	-2.2599835476	-0.878181766
C	4.9243899376	-2.1766476676	-0.093606192
H	5.3689980924	-3.062713984	0.3453783192
C	5.5172501308	-0.9203581501	0.1498063217
C	4.9290479089	0.2763230513	-0.3667030748
C	3.767187107	0.1963292214	-1.1497435852
H	-0.8581995754	-5.721702563	-0.6441606788
H	1.5686475768	-5.5609312223	-0.3682069105
H	-0.9325899644	-1.8033891029	-2.4267642828
H	-2.1511877213	-3.8355351288	-1.6608758871
H	3.3121981662	1.0930433937	-1.5653409688
C	6.6884184361	-0.5678866246	0.9096324658
O	7.5068736158	-1.2840883708	1.5379610065
C	5.7195919085	1.4079608449	0.0585404044
O	5.5765360902	2.6343172732	-0.1618050031
N	6.7732087305	0.852027971	0.8263381115
N	3.15046795	-3.5044199024	-1.1634250757
S	1.8714254379	-1.1734094711	-2.5840943702
C	7.8035717071	1.626387949	1.449022537
C	8.9868741809	1.9080864468	0.7280984149
C	7.6159317158	2.0906406829	2.771476435
C	9.9831597929	2.6671514005	1.3601361419
C	8.6415515598	2.8452702178	3.3605188696
C	9.8153919205	3.1335237016	2.6640720907
H	10.9012464348	2.8955185103	0.8244077484
H	8.5191906477	3.211901152	4.3767591659
H	10.5993111334	3.7207530579	3.1375248958
C	6.3461262482	1.7961193599	3.5658901821
H	5.6803987776	1.2086775012	2.9264247421
C	5.5978041093	3.0883907899	3.9490942849
H	6.1930968423	3.7096957626	4.6301145239
H	5.3572143133	3.6908327148	3.0650285543
H	4.6564241205	2.8431053639	4.4574965657
C	6.6425583227	0.9542899466	4.8231168153
H	7.1538414294	0.0183802495	4.5683907027
H	7.2749297218	1.5032765104	5.5323278849
H	5.7067935852	0.6995394728	5.3372324942
C	9.2090480676	1.4140440998	-0.6990142543
H	8.3176530158	0.8571629236	-1.0031183655
C	9.3845908621	2.5831236661	-1.6885048123
H	10.2907183414	3.1617823166	-1.4687468648
H	9.4730284868	2.2003952425	-2.7134751069
H	8.5294029987	3.2686545568	-1.6565443827
C	10.4094086337	0.4505723873	-0.7881924054
H	11.3493745658	0.9578235586	-0.536303687
H	10.29208341	-0.4013400328	-0.1079023257
H	10.5043089595	0.0579055447	-1.8088194105
C	3.92001901	-4.7318653651	-0.9399133377
H	4.927352047	-4.5511956734	-1.3240213914
H	3.4787970246	-5.5180873494	-1.559360753
C	3.9900129453	-5.2505417843	0.5151341775
H	5.0218282167	-5.2222148853	0.8942724252
H	3.3704500925	-4.6320757144	1.181189434
C	3.5855087445	-7.1843739428	1.8306872689
H	4.6224547552	-7.1866606626	2.2004504805
H	2.9703129512	-6.606808352	2.5378686565
C	3.063624678	-8.6093392246	1.7221965055
H	3.6762840827	-9.1855381691	1.0116536154
H	2.02469036	-8.6075179123	1.3579903533
O	3.5256791648	-6.6009903182	0.5339457706
O	3.1313926002	-9.1924094274	3.0181413267
C	2.6673302075	-10.5372094027	3.0453211
H	3.2676122711	-11.1620044479	2.3659899726
H	1.616874935	-10.5854331049	2.7189720669
C	2.7961978116	-11.0421895061	4.4744777499
H	2.1985170164	-10.4142147699	5.1539282025
H	3.8474058547	-10.9947736827	4.7999979184
C	2.4071285207	-12.9581515167	5.8038310408
H	2.0276486354	-13.9810661667	5.7276743715
H	3.4460232428	-12.983565546	6.1666406827
H	1.7926177025	-12.3965579444	6.5239272959
O	2.3298346084	-12.3848555384	4.5049073215

cationX.out

C	-4.6960359184	4.0058944266	-0.1130704559
C	-3.8023299263	3.1110127165	-0.6725889044
C	-2.4784074415	2.987703621	-0.1790713961
C	-2.1289516706	3.804129886	0.9366332673
C	-3.0481331326	4.7121447702	1.4926938959
C	-4.323896253	4.8227683304	0.9701757665
C	0.2380478864	2.474475385	0.8716455593
C	-0.3151112857	1.7949503621	-0.2573304193
C	0.4697690078	0.7678231401	-0.8576062688
H	0.117946032	0.1949045844	-1.7023582753
C	1.7054519583	0.4829314802	-0.3324982084
C	2.2313659865	1.1618369283	0.7737849994
C	1.5144140842	2.1592523832	1.3880392098
H	-5.7023966618	4.0637334907	-0.5165355365
H	-4.1501734567	2.4731191525	-1.4700362429
H	-2.7468829074	5.3227345385	2.3397030349
H	-5.0305441977	5.5245294197	1.4019554403
H	1.9056034721	2.6987125552	2.246413502
C	2.7030680912	-0.5391011895	-0.7659903323
O	2.6228477398	-1.331353159	-1.6862865777
C	3.5762775105	0.5964251057	1.0753927697
O	4.3532102789	0.9142223524	1.9564876926
N	3.7908981577	-0.4125692311	0.1167707984
N	-1.5707767227	2.1092126444	-0.7808113472
S	-0.5632062744	3.7812533864	1.6898329501
C	4.9837664146	-1.2199367989	0.048917295
C	6.0660717139	-0.7642696182	-0.7348884926
C	5.0218613516	-2.4341733527	0.7683481154
C	7.2126920828	-1.5701287887	-0.784320313
C	6.1936346757	-3.1997777895	0.6826036327
C	7.2778138468	-2.7746928861	-0.0845996043
H	8.0640448466	-1.2493803733	-1.3786339571
H	6.2564375376	-4.1397198722	1.2242421789
H	8.1770211983	-3.3840643216	-0.1367171101
C	3.8561787545	-2.9307070826	1.6194928435
H	3.0450593102	-2.1986695148	1.5535530867
C	4.2493374751	-3.0457274739	3.1057690095
H	5.0298770233	-3.8018883233	3.2549375708
H	4.6218818886	-2.0918640773	3.4977579631
H	3.3775401791	-3.3410224265	3.7031722579
C	3.3052612574	-4.2730759769	1.0989267348
H	3.0014677634	-4.202396265	0.0477303709
H	4.0529242499	-5.0712486819	1.1829472267
H	2.4281197265	-4.5721688084	1.6865171603
C	6.0309124975	0.5480913527	-1.5137083437
H	5.0602427933	1.0244138382	-1.3430193308
C	7.1146341364	1.52815131	-1.0220580105
H	8.1220051784	1.1347549367	-1.2055539505
H	7.025700344	2.4837998201	-1.5539596305
H	7.0189223301	1.7274449641	0.051897579
C	6.1639348613	0.3099663463	-3.031118719
H	7.140029994	-0.1223215776	-3.2834782163
H	5.3863895378	-0.3688708326	-3.4011563644

H	6.0690175656	1.2609858344	-3.5702230702
C	-1.939315801	1.5055169174	-2.0966673715
H	-1.0367298126	1.4733622374	-2.7059615842
H	-2.6109802911	2.194989197	-2.6023351405
C	-2.5509751141	0.1075776768	-2.0255422982
H	-2.6141230833	-0.2631501545	-3.060195465
H	-1.9145982548	-0.5823175585	-1.4558638414
C	-4.4597539692	-1.1173358008	-1.3437597035
H	-4.5797152458	-1.5544012539	-2.3462888952
H	-3.8359161271	-1.7950873154	-0.7422226354
C	-5.8169766593	-0.9243692787	-0.6844303803
H	-6.4386952357	-0.2441419892	-1.2867195607
H	-5.6935522847	-0.4830316907	0.3165260913
O	-3.8414384764	0.1647775489	-1.4382321726
O	-6.4324846712	-2.2026101437	-0.5887460099
C	-7.7219015894	-2.1508103288	0.0121179915
H	-8.3911776669	-1.5048454021	-0.5767783202
H	-7.6518571942	-1.7373258795	1.0300180158
C	-8.2693569293	-3.5692588944	0.0590465614
H	-7.5991380742	-4.2153594168	0.6478010601
H	-8.3377566985	-3.9836116893	-0.9592216061
C	-10.1659621421	-4.7972794452	0.7573674356
H	-11.1429120838	-4.6538912198	1.227574189
H	-10.3052480439	-5.2495242446	-0.2363808453
H	-9.5624908766	-5.4781841936	1.3768031136
O	-9.5570818043	-3.516015369	0.6586729064

V. References

[1] H. Chen, P. Dao, F. Ye and G. Huang, *Faming Zhuanli Shenqing*, CN106046027 A, 2016.

[2] G. M. Fischer, M. Isomaki-Krondahl, I. Gottker-Schnetmann, E. Daltrozzo, A. Zumbusch, *Chem.*, 2009, **15**, 4857-4864.

[3] M. Banno, T. Yamaguchi, K. Nagai, C. Kaiser, S. Hecht, E. Yashima, *J. Am. Chem. Soc.* 2012, **134**, 8718-8728.

[4] A. Ali, H. Siddiki, K. Kon, J. Hasegawa and K.-I. Shimizu, *Chem. Eur. J.*, 2014, **20**, 14256-14260.

[5] C. S. Sevov, S. K. Samaroo and M. S. Sanford, *Adv. Energy Mat.*, 2017, **7**, 1602027.

[6] J. A. Kowalki, B. J. Neyhouse and F. R. Brushett, *Electrochem. Commun.*, 2020, **111**, 106625.

[7] (*a*) J. A. Kowalki, M. D. Casselman, A. P. Kaur, J. D. Milshtein, C. F. Elliott, S. Modekrutti, N. H. Attanayake, N. Zhang, S. R. Parkin, C. Risko, F. R. Brushett and S. A. Odom, *J. Mater. Chem. A*, 2017, **5**, 24371-24379. (*b*) J. D. Milshtein, J. L. Barton, R. M. Darling and F. R. Brushett, *J. Power Sources*, 2016, **327**, 151-159. (*c*) J. D. Milshtein, K M. Tenny, J. L. Barton, J. Drake, R. M. Darling and F. R. Brushett, *J. Electrochem. Soc.*, 2017, **164**, E3265-E3275. (*d*) J. D. Milshtein, E. L. Fisher, T. M. Breault, L. T. Thompson and F. R. Brushett, *ChemSusChem*, 2017, **10**, 2080-2088. (*e*) J. D. Milshtein, J. L. Barton, T. J. Carney, J. A. Kowalski, R. M. Darling and F. R. Brushett, *J. Electrochem. Soc.*, 2017, **164**, A2487-A2499.

[8] R. S. Nicholson, *Anal. Chem.* 1965, **37**, 1351-1355.

[9] I. Lavagnini, R. Antiochia and F. Magno, *Electroanalysis*, 2004, **16**, 505-506.

[10] P. Pracht, F. Bohle and S. Grimme, Automated Exploration of the Low-Energy Chemical Space with Fast Quantum Chemical Methods, *Phys. Chem. Chem. Phys.* 2020, **22** (14), 7169–7192.

[11] Gaussian 16, Revision B.01, M. J. Frisch, G. W. Trucks, H. B. Schlegel, G. E. Scuseria, M. A. Robb, J. R. Cheeseman, G. Scalmani, V. Barone, G. A. Petersson, H. Nakatsuji, X. Li, M. Caricato, A. V. Marenich, J. Bloino, B. G. Janesko, R. Gomperts, B. Mennucci, H. P. Hratchian, J. V. Ortiz, A. F. Izmaylov, J. L. Sonnenberg, D. Williams-Young, F. Ding, F. Lipparini, F. Egidi, J. Goings, B. Peng, A. Petrone, T. Henderson, D. Ranasinghe, V. G. Zakrzewski, J. Gao, N. Rega, G. Zheng, W. Liang, M. Hada, M. Ehara, K. Toyota, R. Fukuda, J. Hasegawa, M. Ishida, T. Nakajima, Y. Honda, O. Kitao, H. Nakai, T. Vreven, K. Throssell, J. A. Montgomery, Jr., J. E. Peralta, F. Ogliaro, M. J. Bearpark, J. J. Heyd, E. N. Brothers, K. N. Kudin, V. N. Staroverov, T. A. Keith, R. Kobayashi, J. Normand, K. Raghavachari, A. P. Rendell, J. C. Burant, S. S. Iyengar, J. Tomasi, M. Cossi, J. M. Millam, M. Klene, C. Adamo, R. Cammi, J. W. Ochterski, R. L. Martin, K. Morokuma, O. Farkas, J. B. Foresman, and D. J. Fox, Gaussian, Inc., Wallingford CT, 2016.

[12] A. V. Marenich, C. J. Cramer and D. G. Truhlar, Universal Solvation Model Based on Solute Electron Density and on a Continuum Model of the Solvent Defined by the Bulk Dielectric Constant and Atomic Surface Tensions, *J. Phys. Chem. B*, 2009, **113**, 6378– 6396.

[13] G. Luchini, J. V. Alegre-Requena, I. Funes-Ardoiz and R. S. Paton, GoodVibes: automated thermochemistry for heterogeneous computational chemistry data [version 1; peer review: 2 approved with reservations]. *F1000Research* 2020, **9**(Chem Inf Sci):291 (https://doi.org/10.12688/f1000research.22758.1).

VII. NMR Spectra

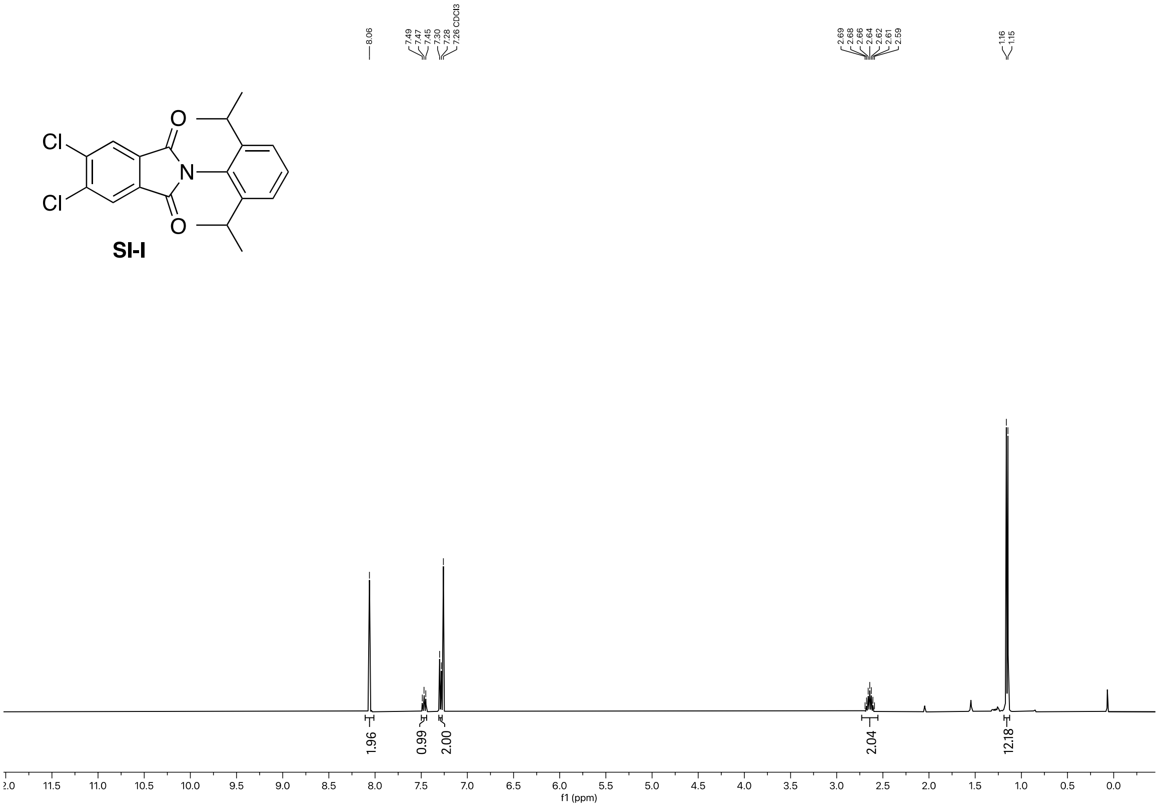

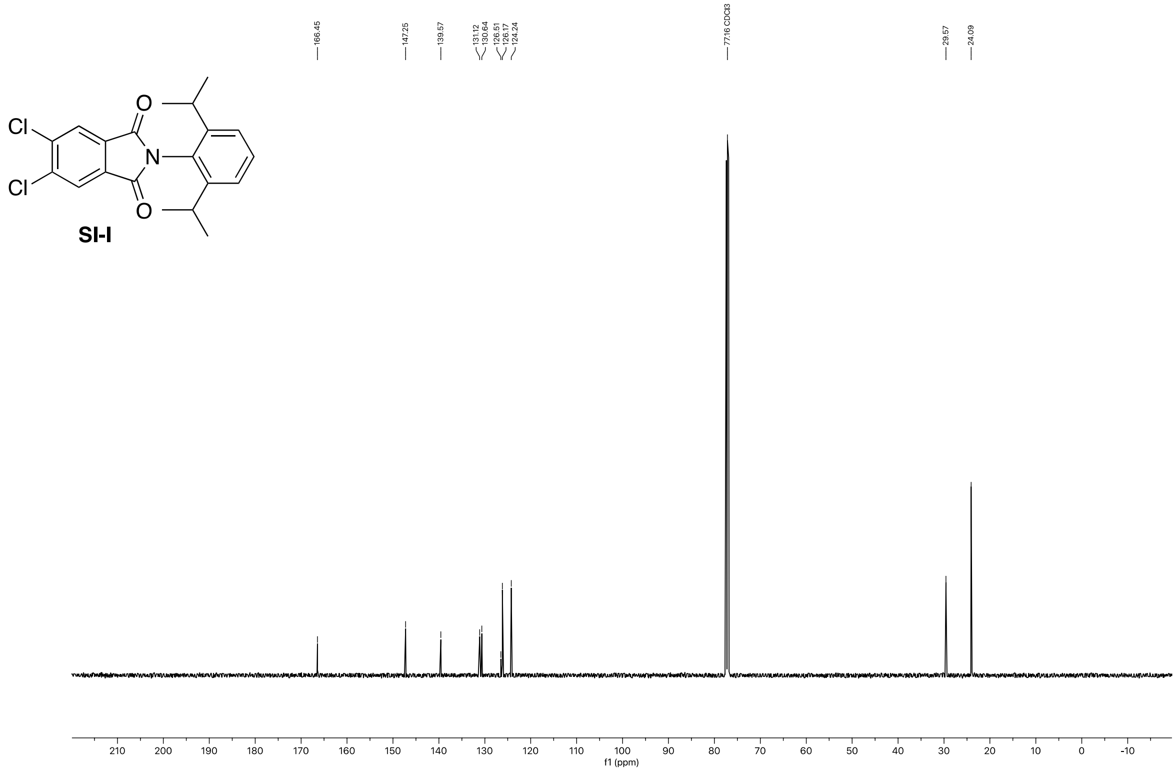

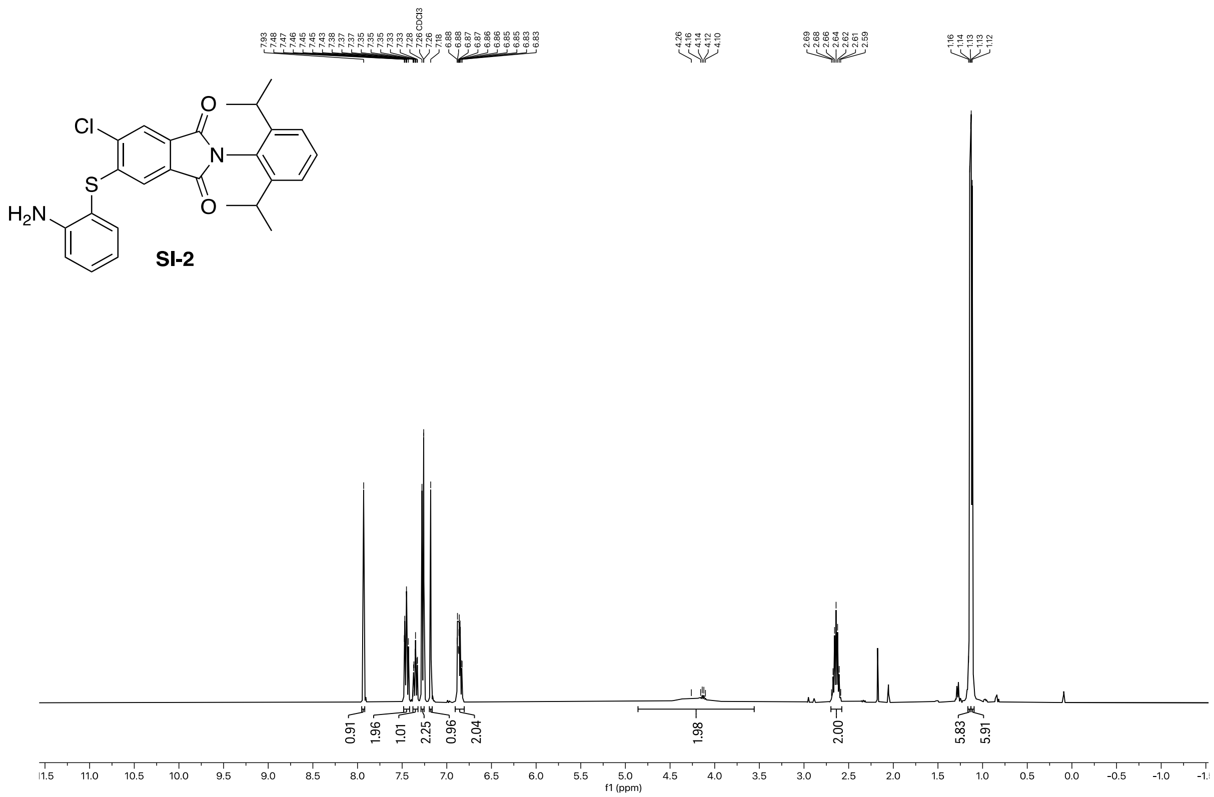
SI-2
f1 (ppm)

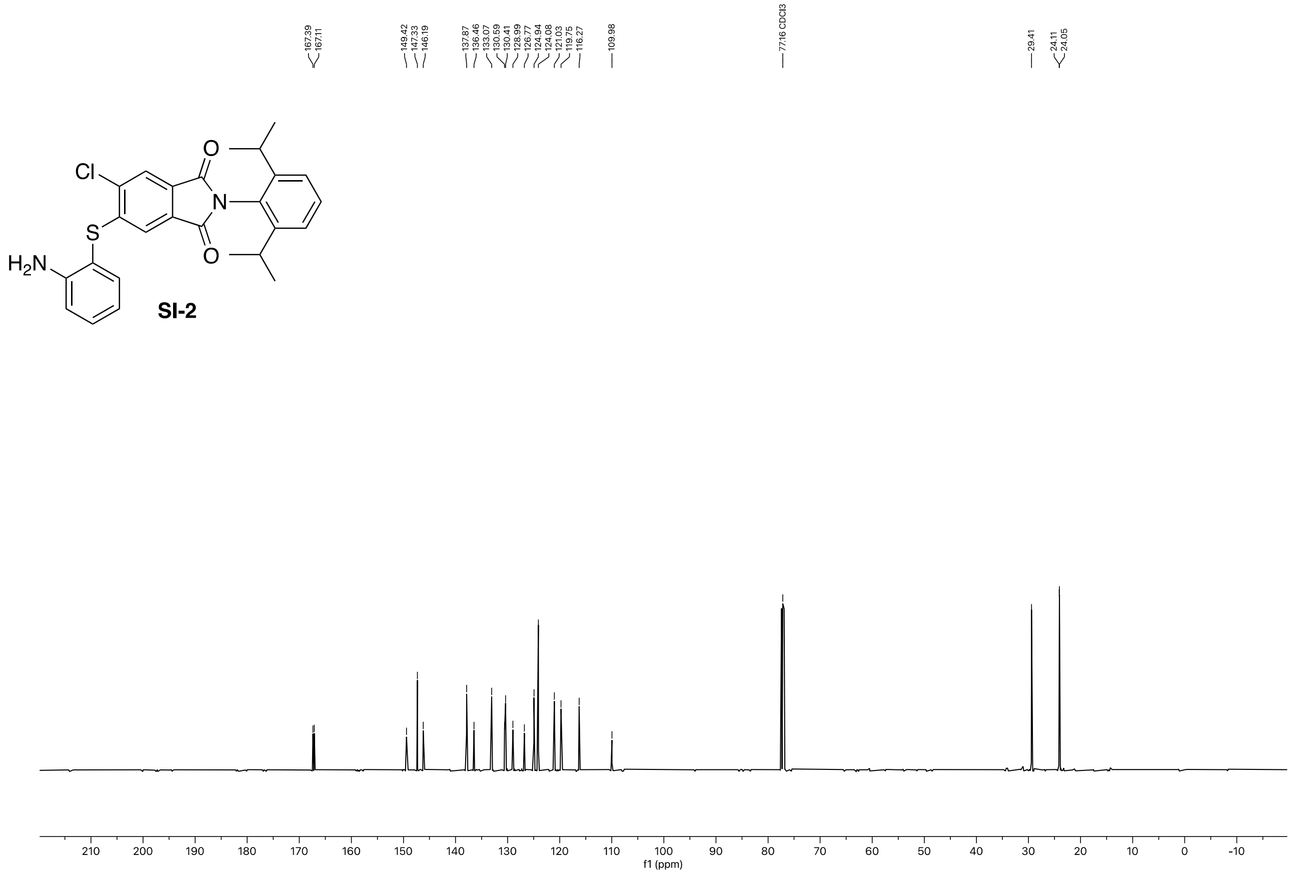
SI-2
77.16 CDCl3
f1 (ppm)

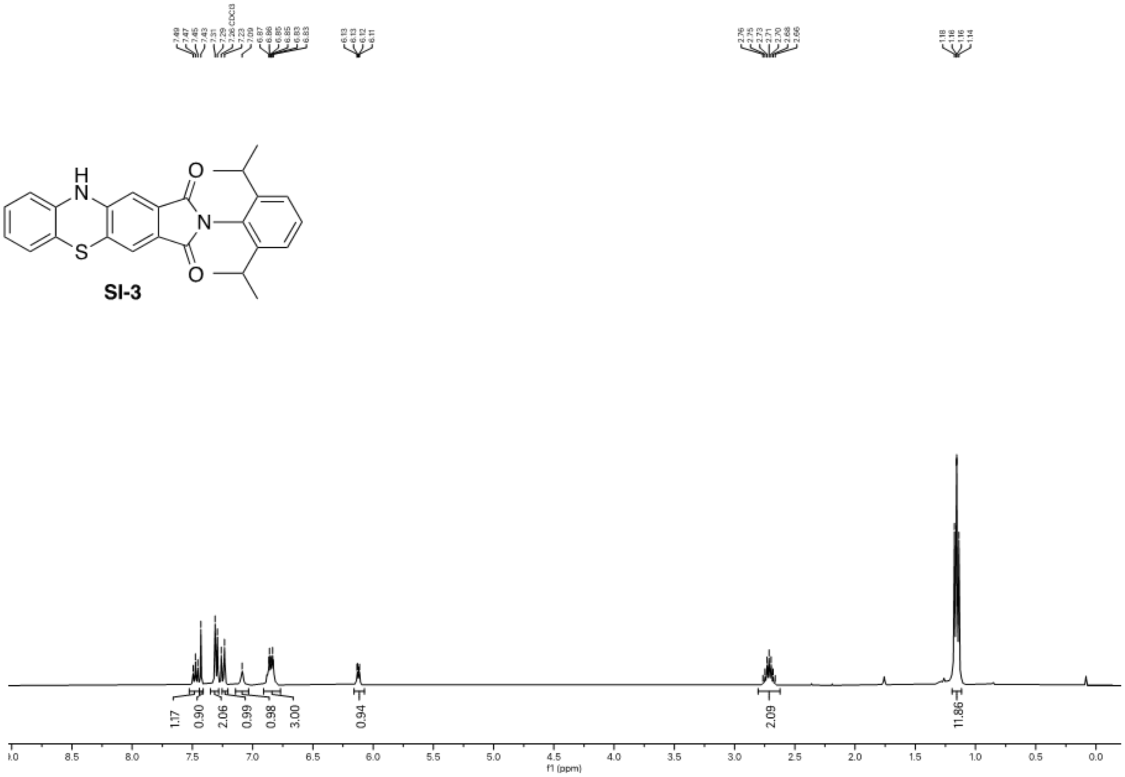
SI-3
f1 (ppm)

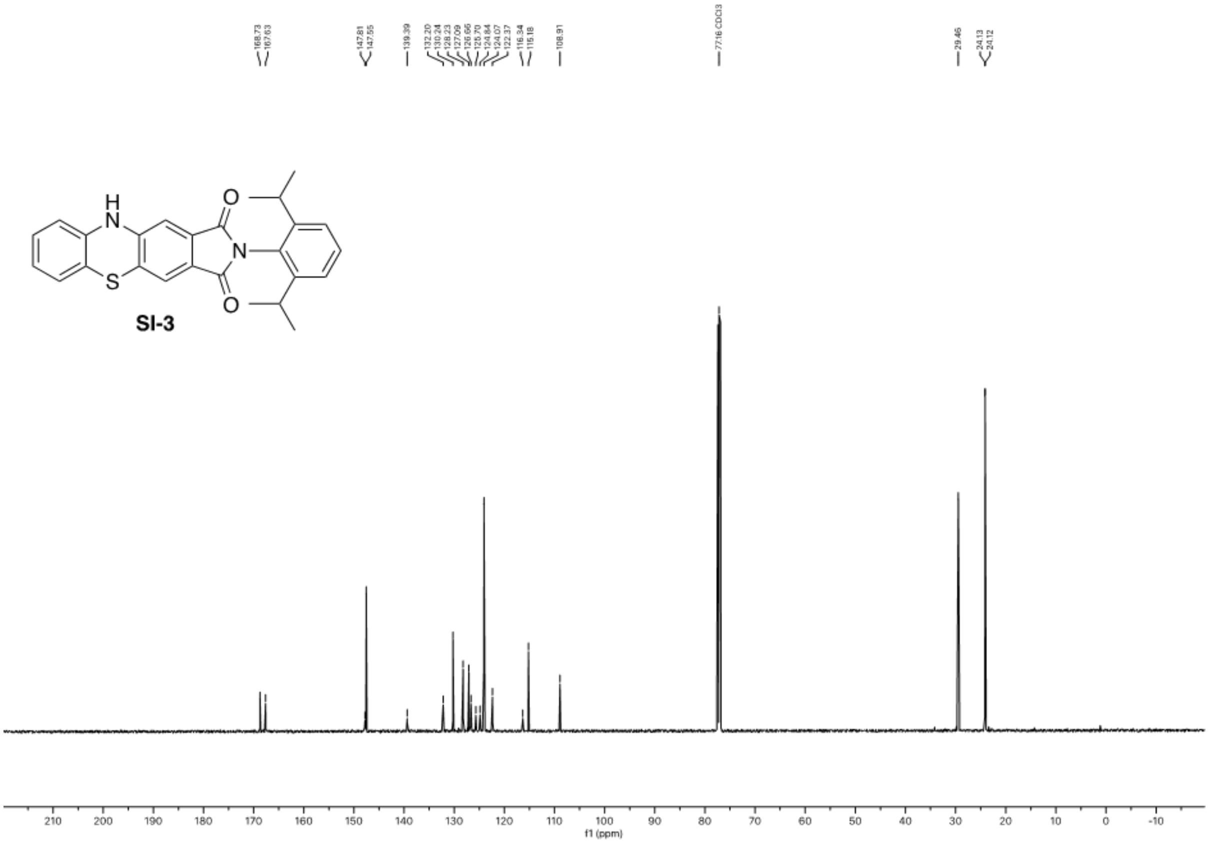
SI-3
f1 (ppm)

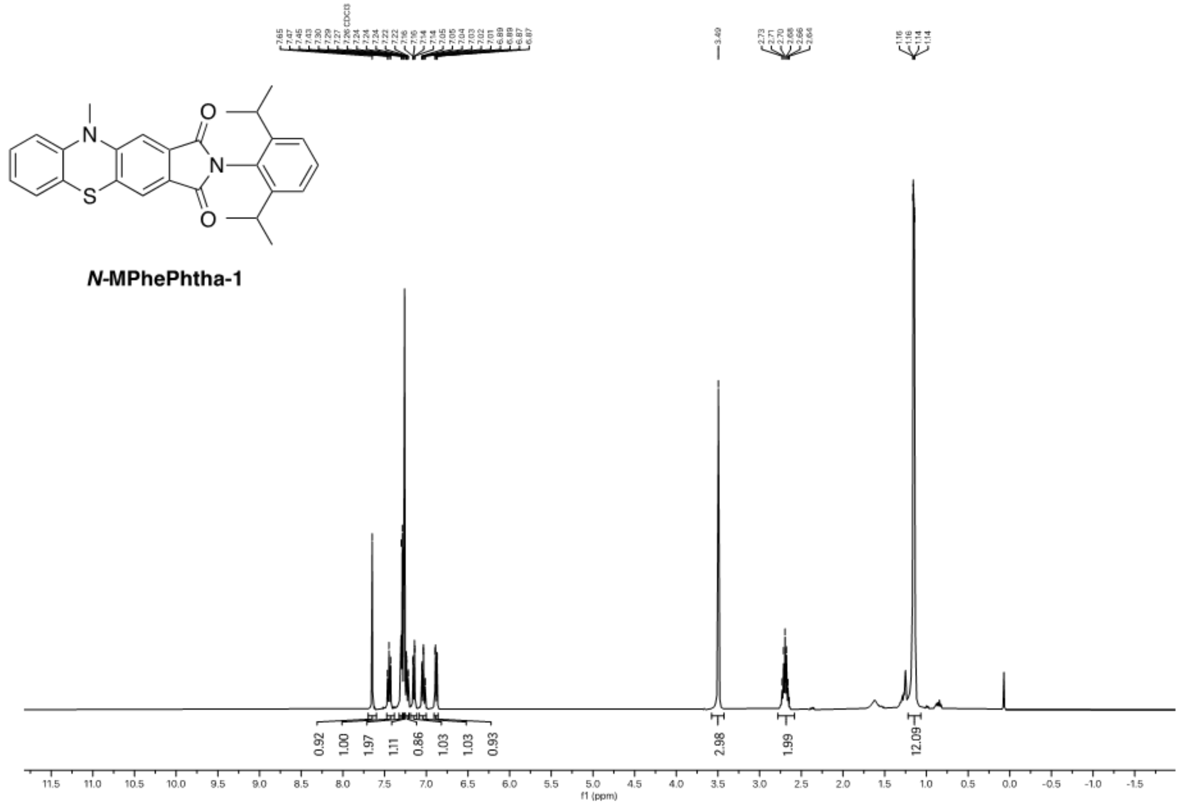

***N*-MPhePhtha-1**

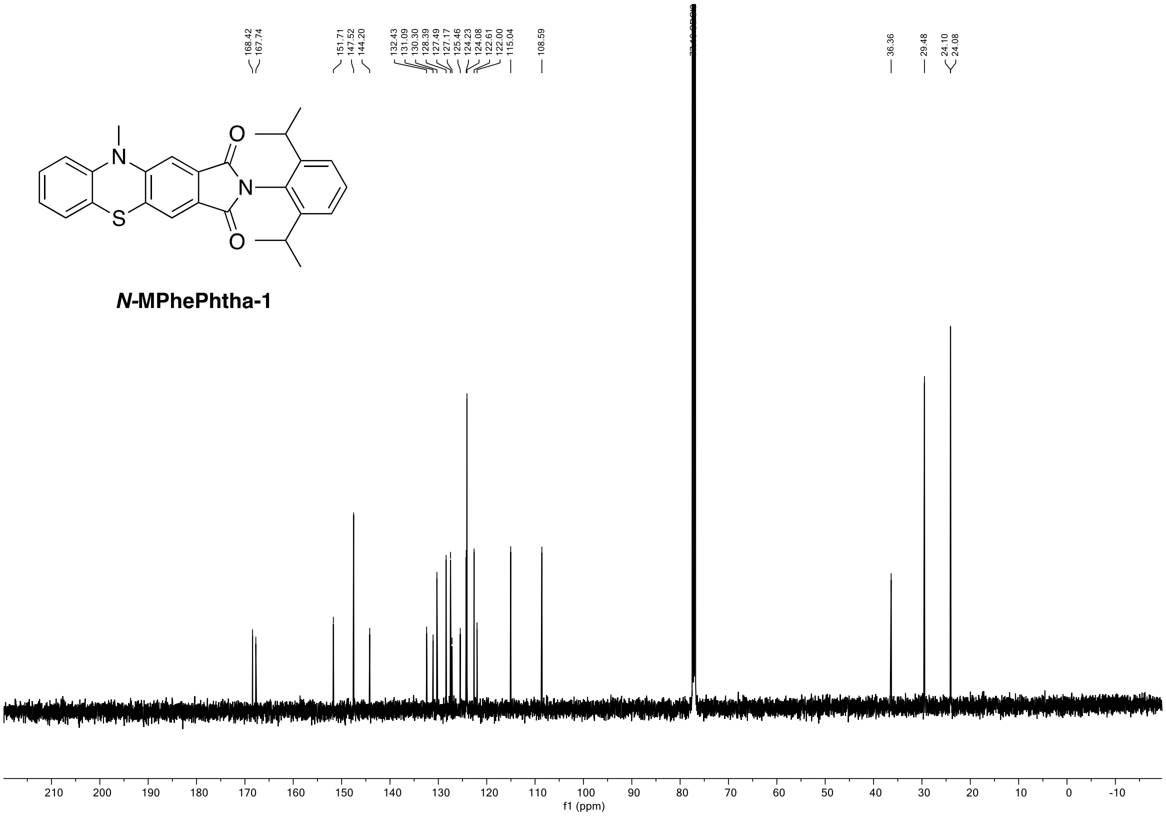

***N*-MPhePhtha-1**

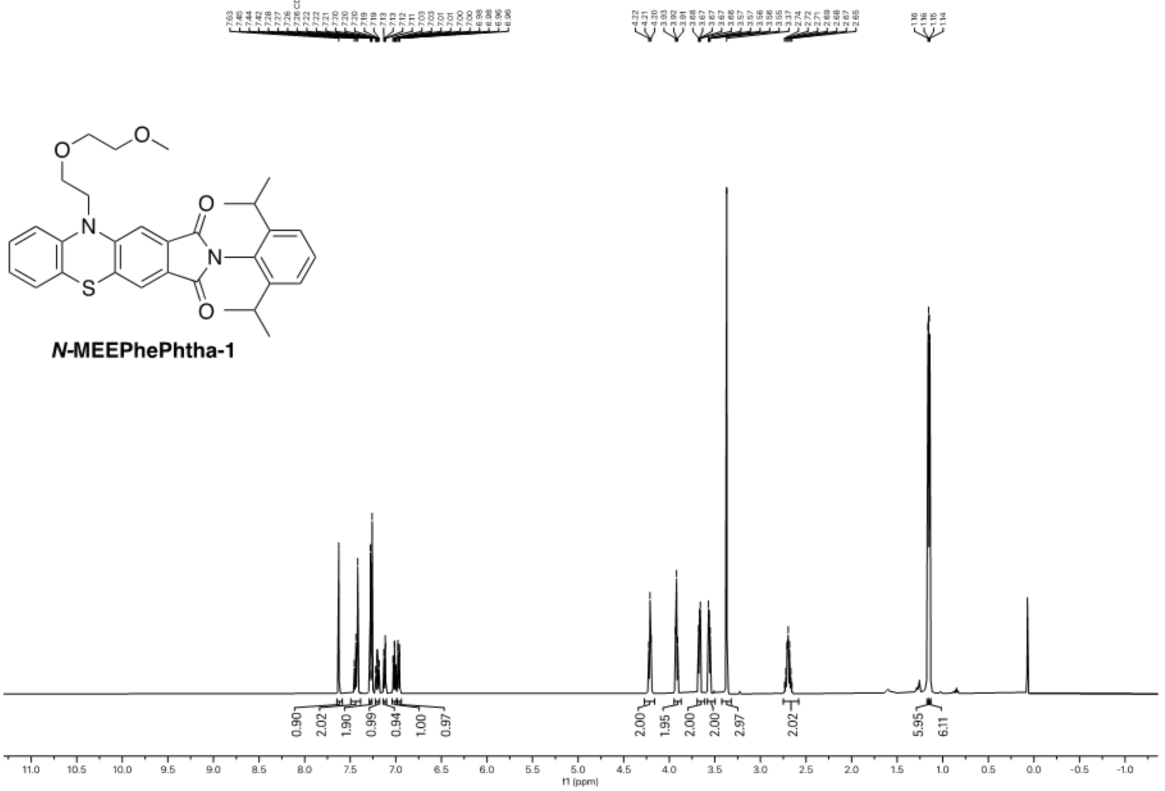
N-MEEPhePhtha-1
f1 (ppm)

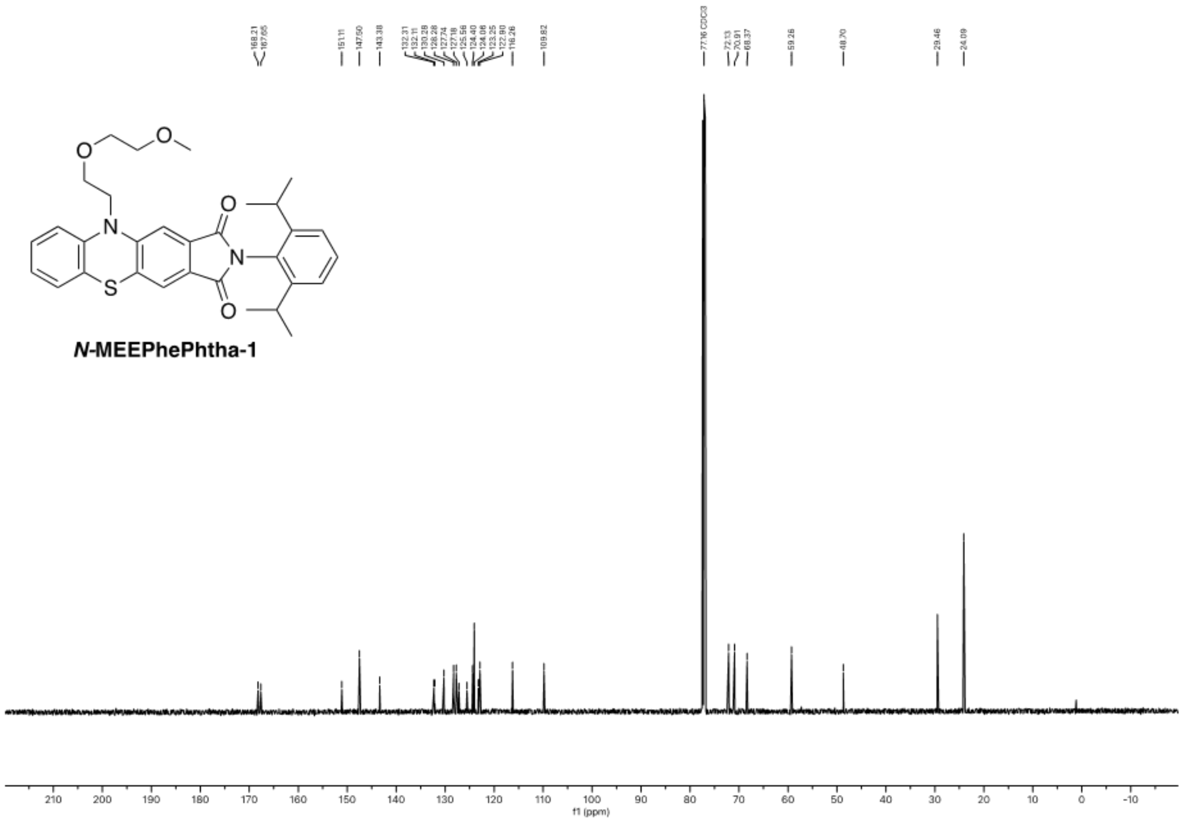
N-MEEPhePhtha-1
f1 (ppm)

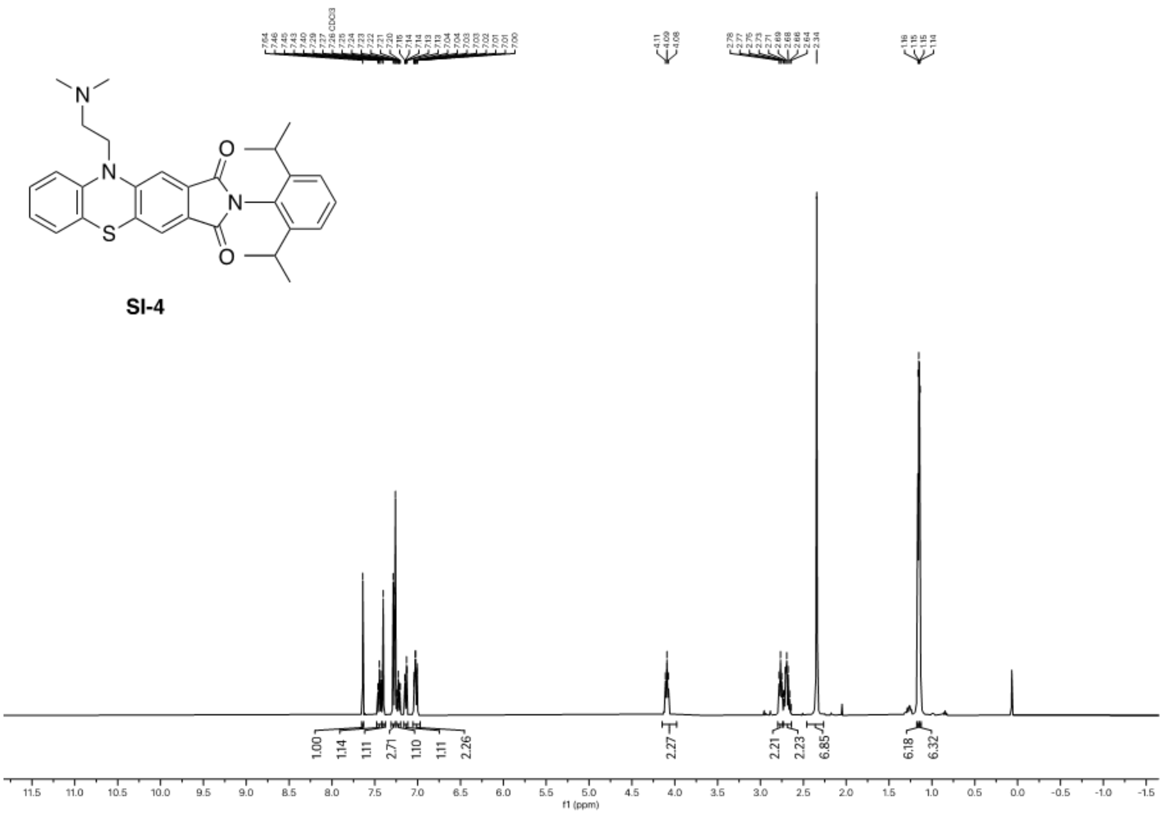

SI-4

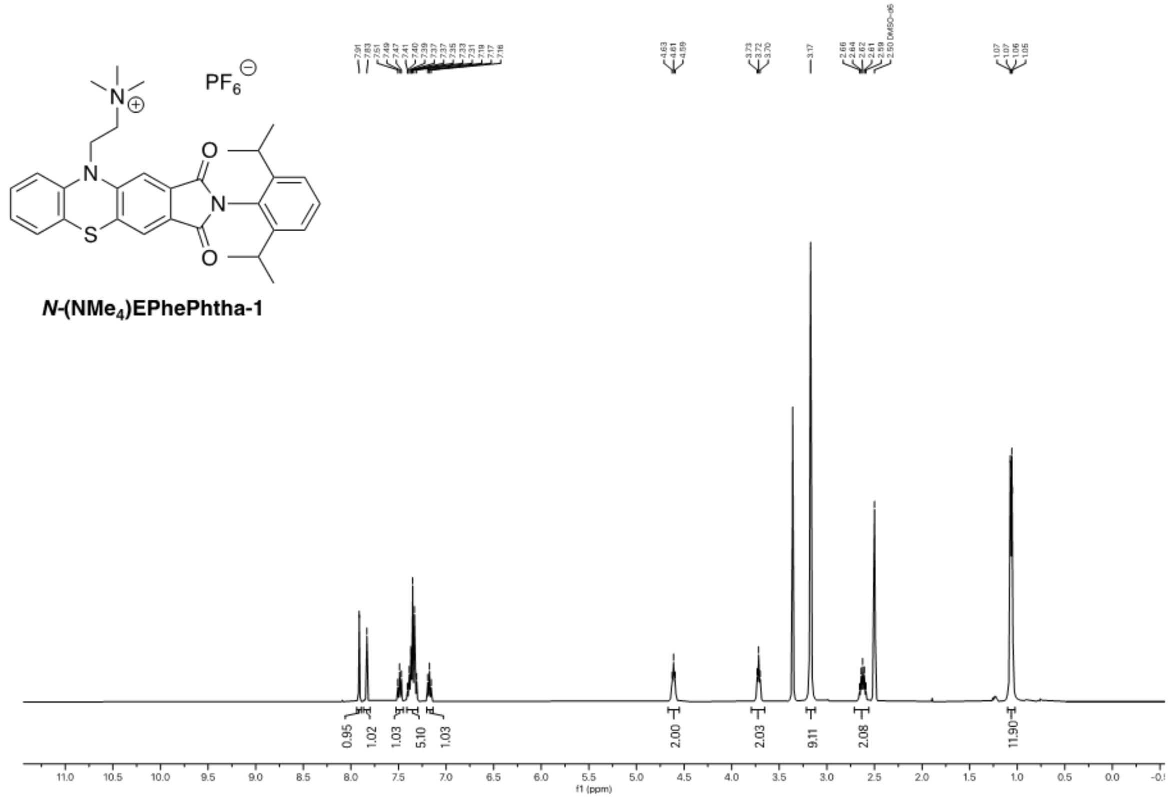

***N*-(NMe$_4$)EPhePhtha-1**

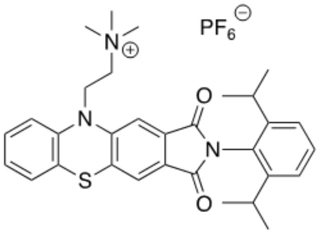

***N*-(NMe$_4$)EPhePhtha-1**

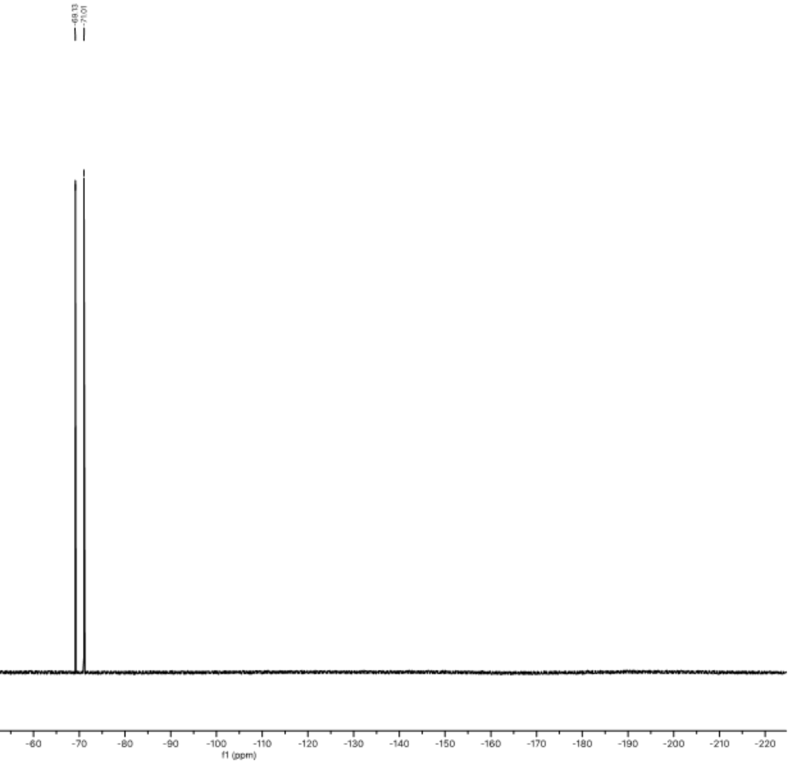

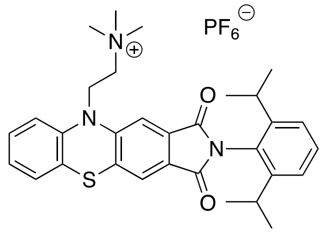

***N*-(NMe$_4$)EPhePhtha-1**

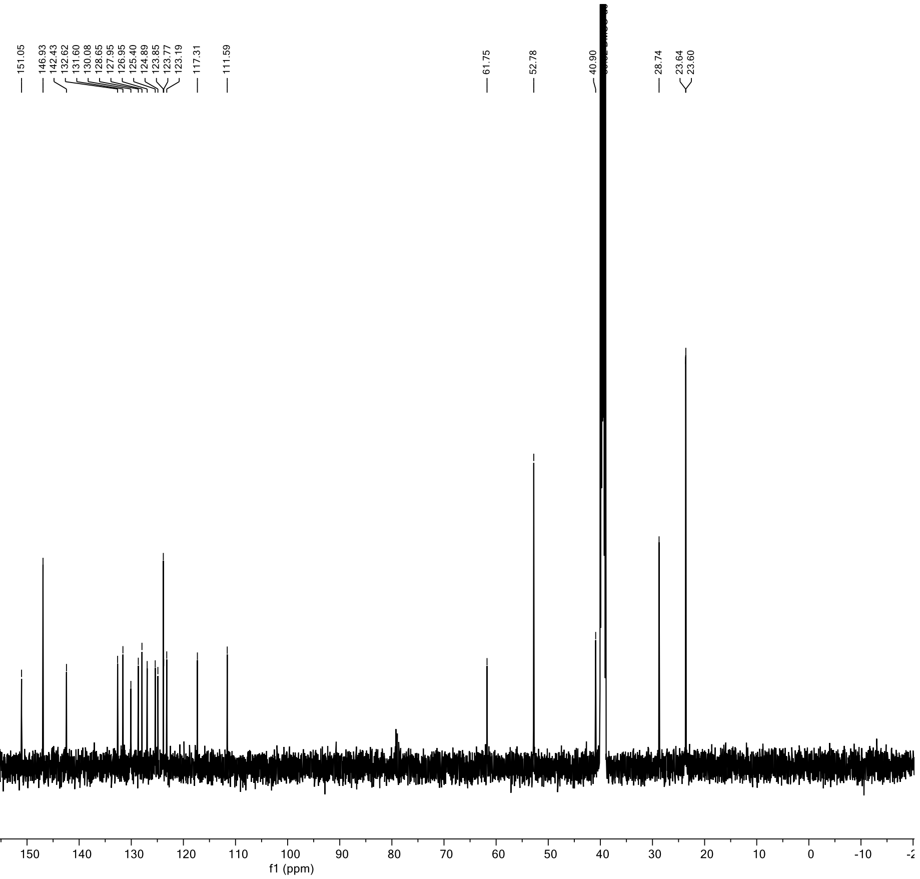

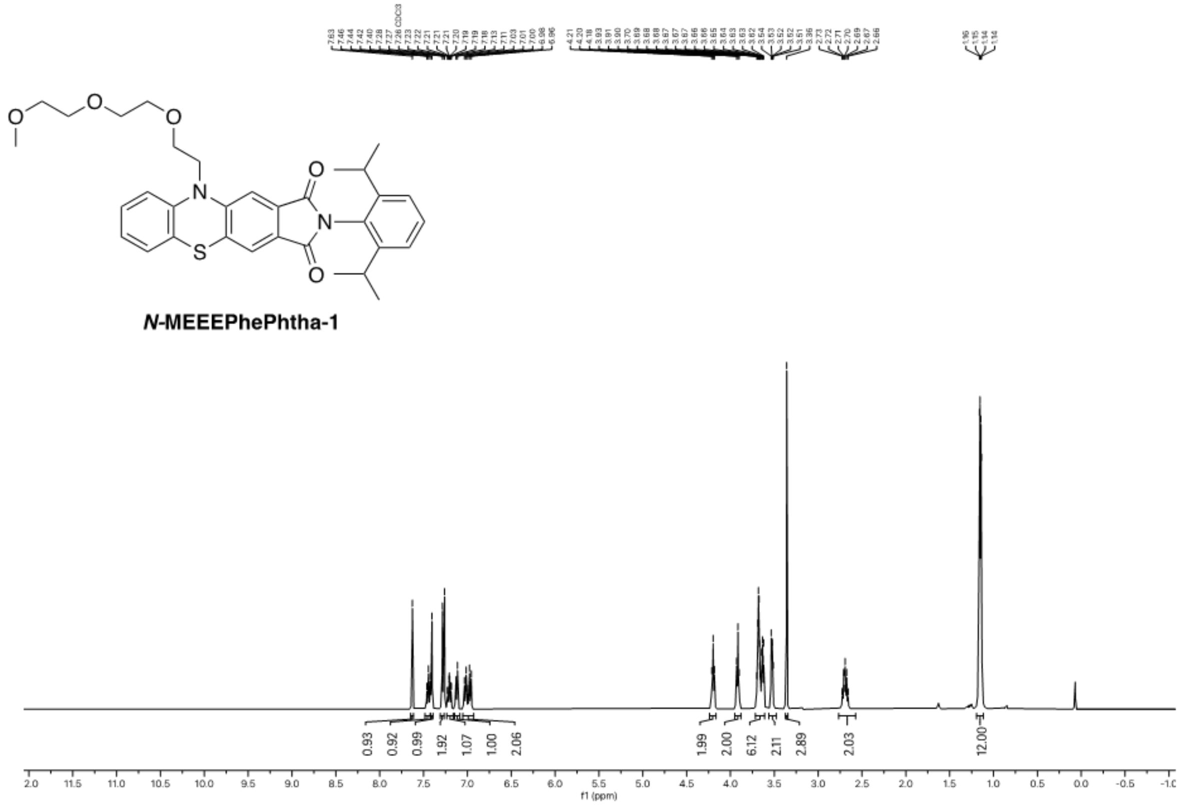
N-MEEEPhePhtha-1
0.93
0.92
0.99
1.92
1.07
1.00
2.06
1.99
2.00
6.12
2.11
2.89
2.03
12.00
f1 (ppm)

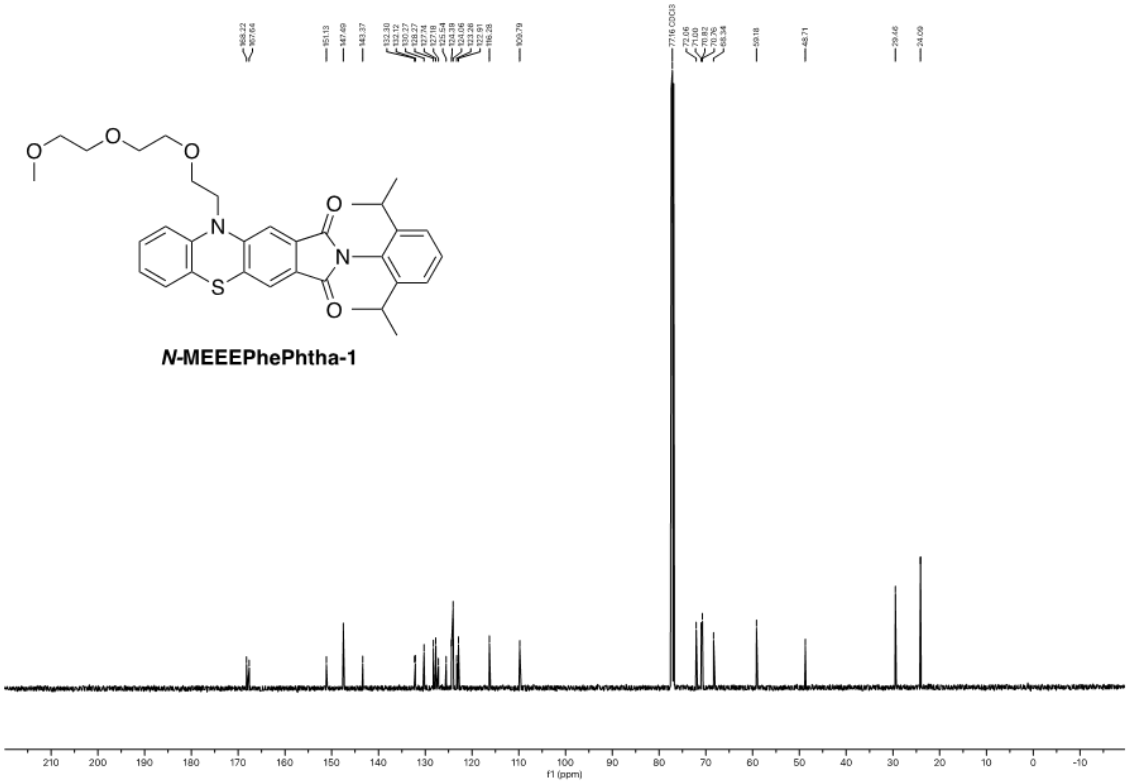
N-MEEEPhePhtha-1
f1 (ppm)

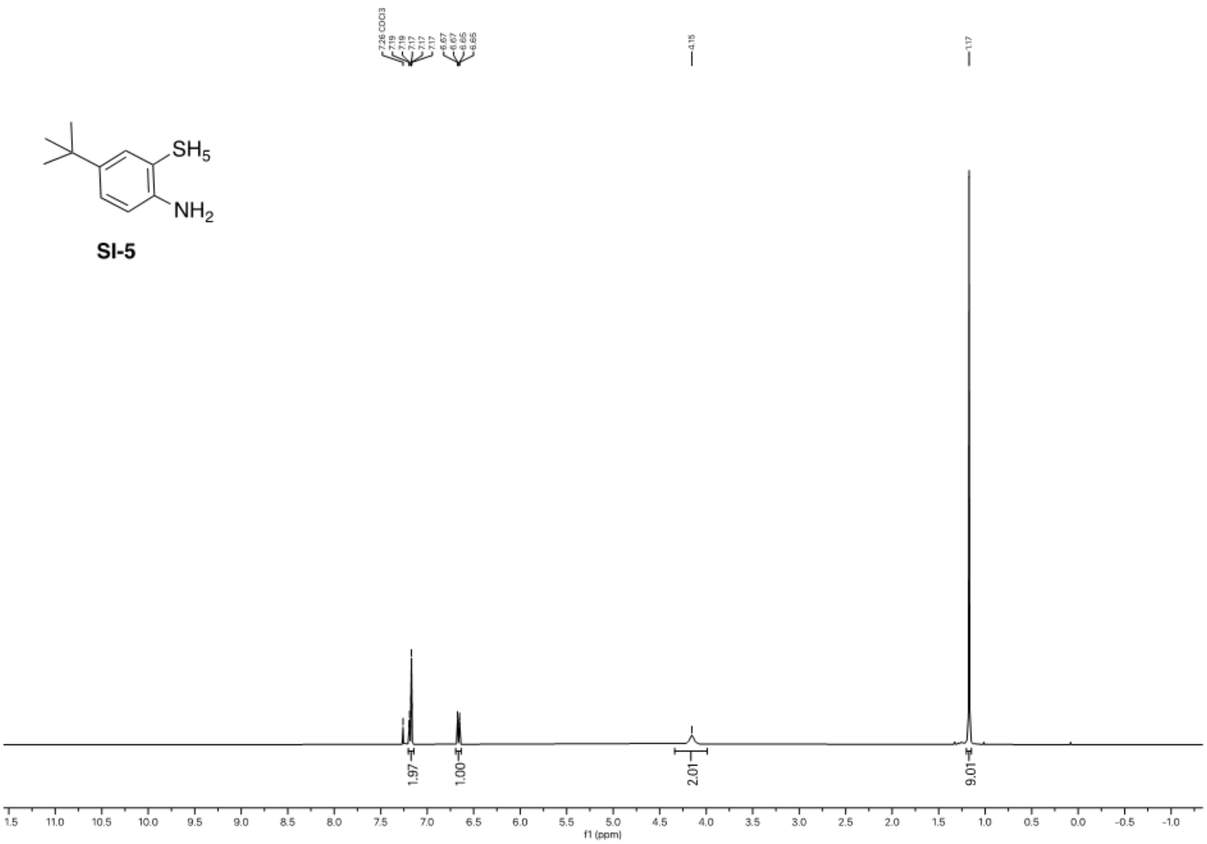
SI-5

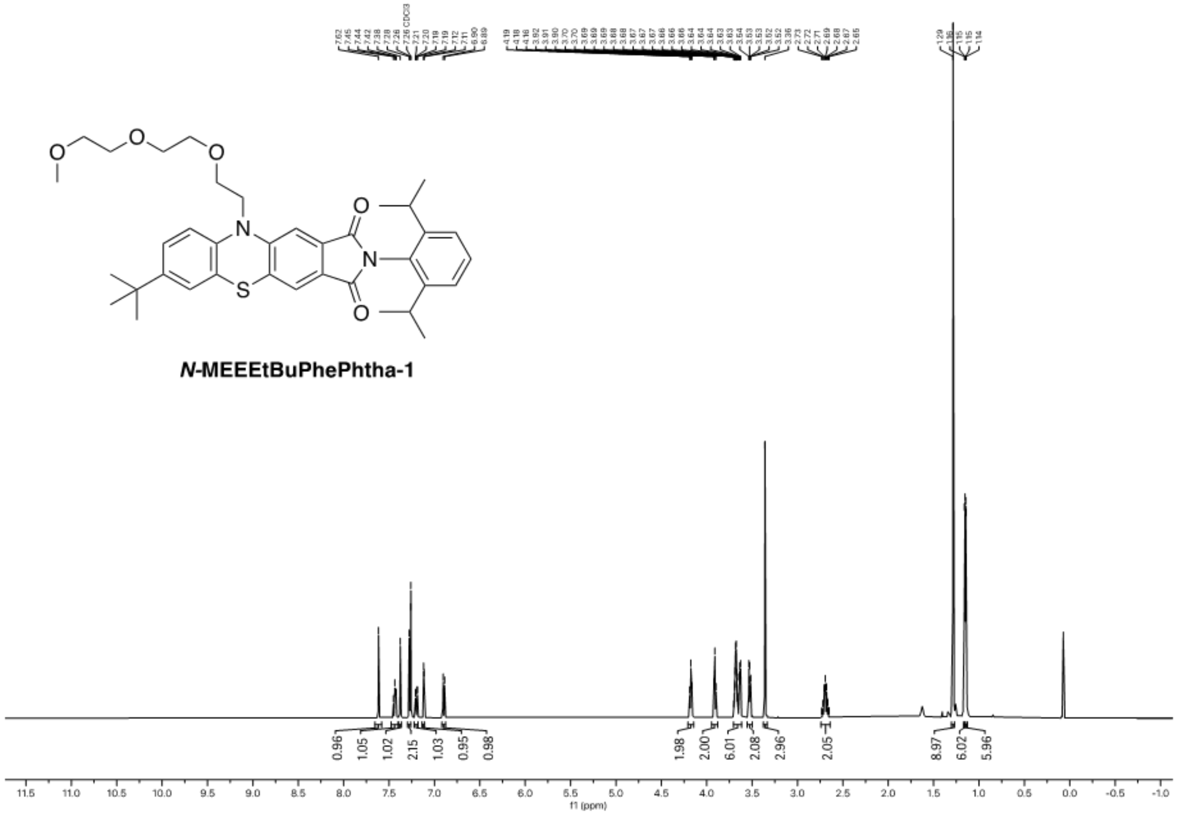
N-MEEEtBuPhePhtha-1
f1 (ppm)

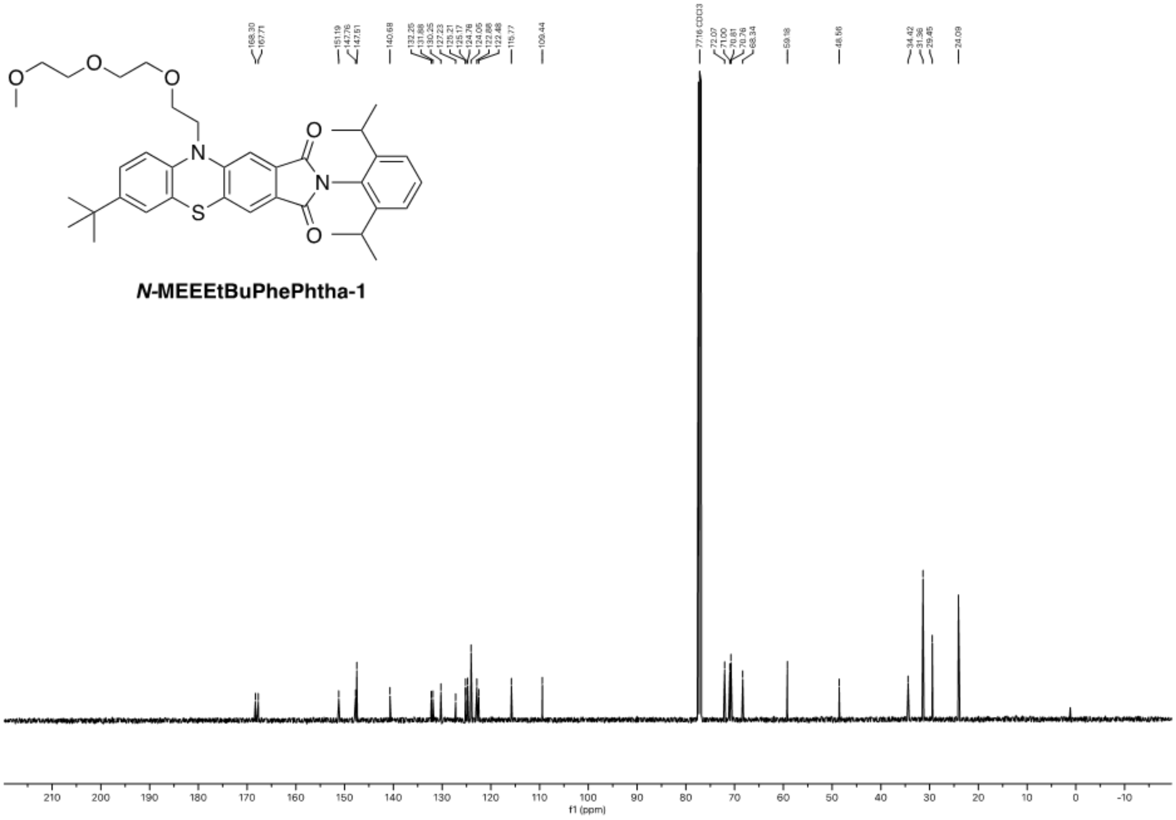
N-MEEEtBuPhePhtha-1
f1 (ppm)

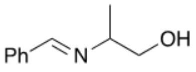

SI-7

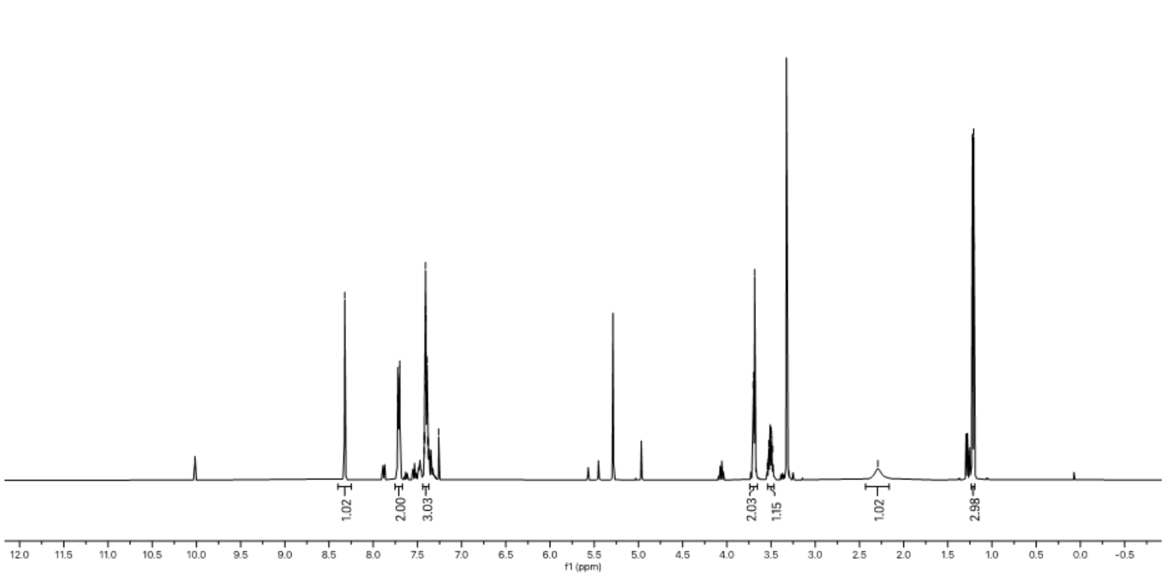

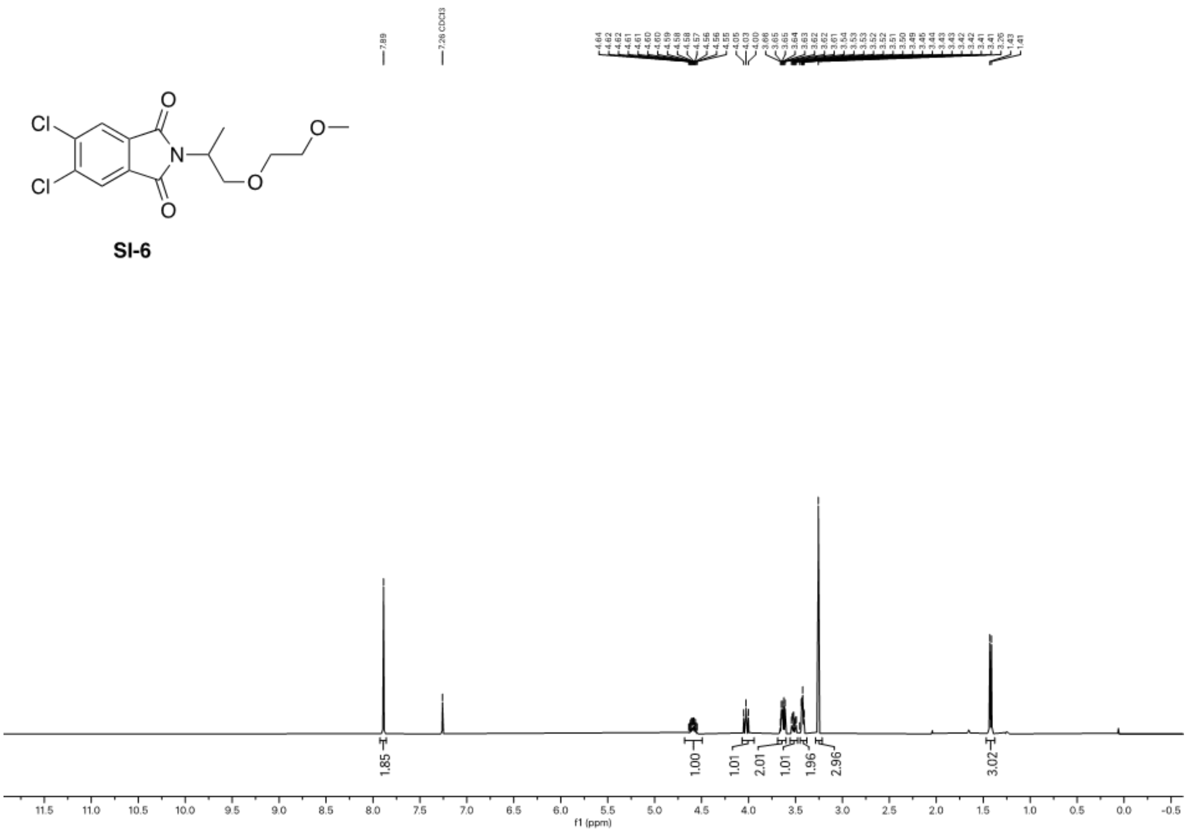
SI-6
f1 (ppm)

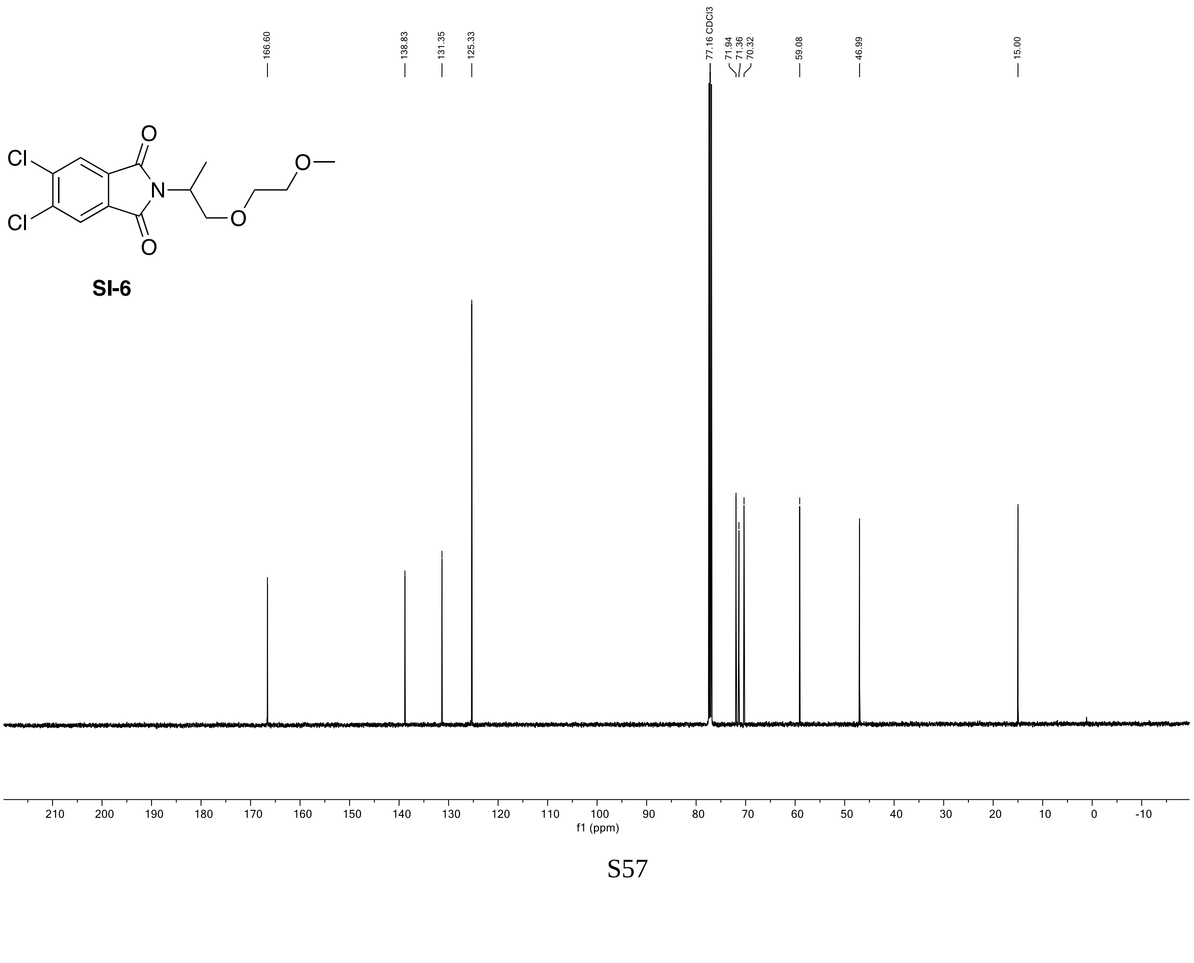
SI-6
f1 (ppm)

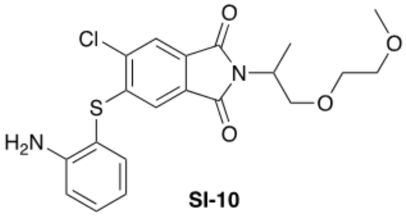

SI-10

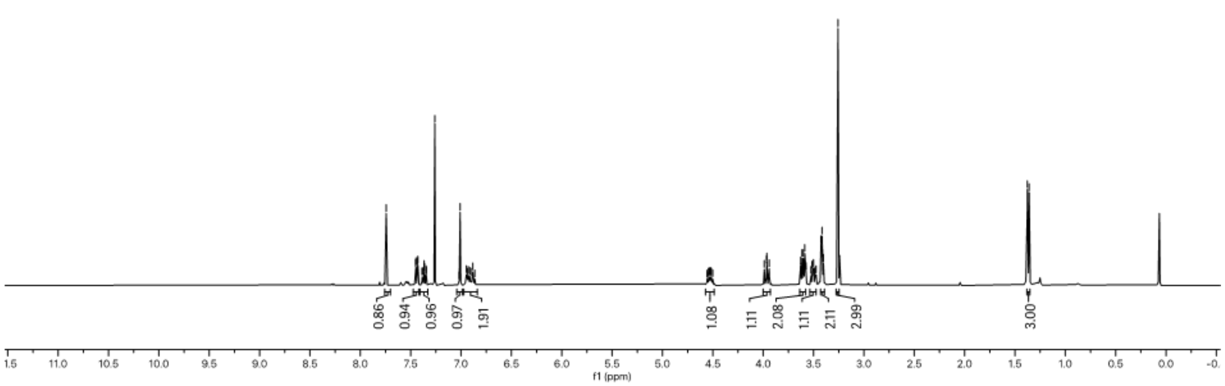

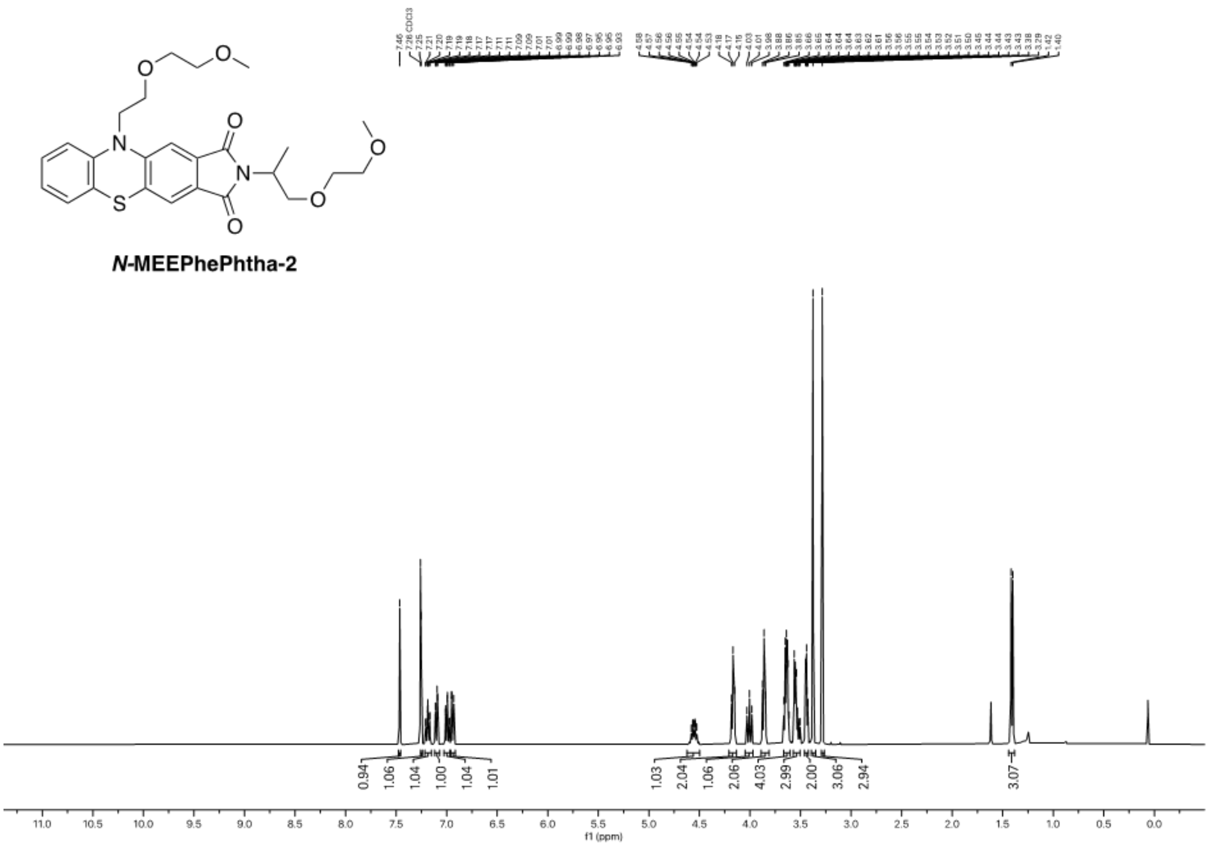
N-MEEPhePhtha-2
0.94
1.06
1.04
1.00
1.04
1.01
1.03
2.04
1.06
2.06
4.03
2.99
2.00
3.06
2.94
3.07
f1 (ppm)

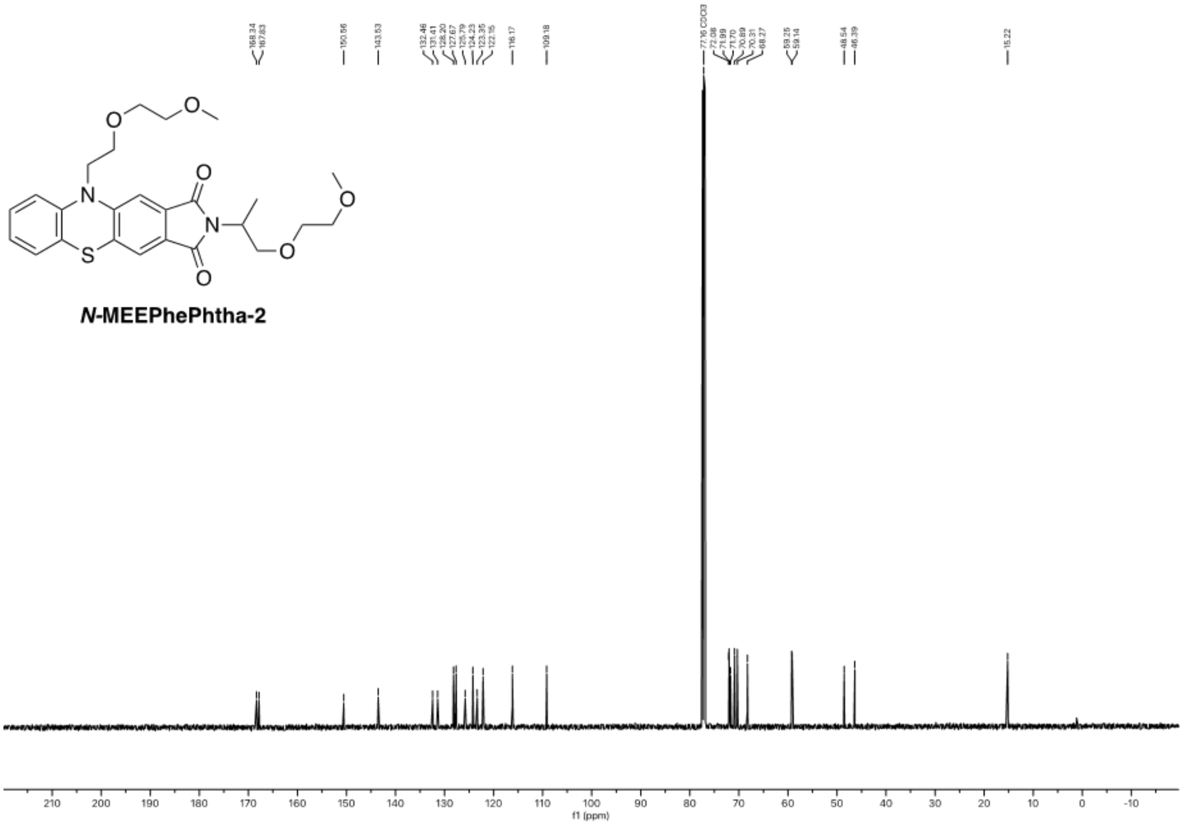
N-MEEPhePhtha-2
f1 (ppm)

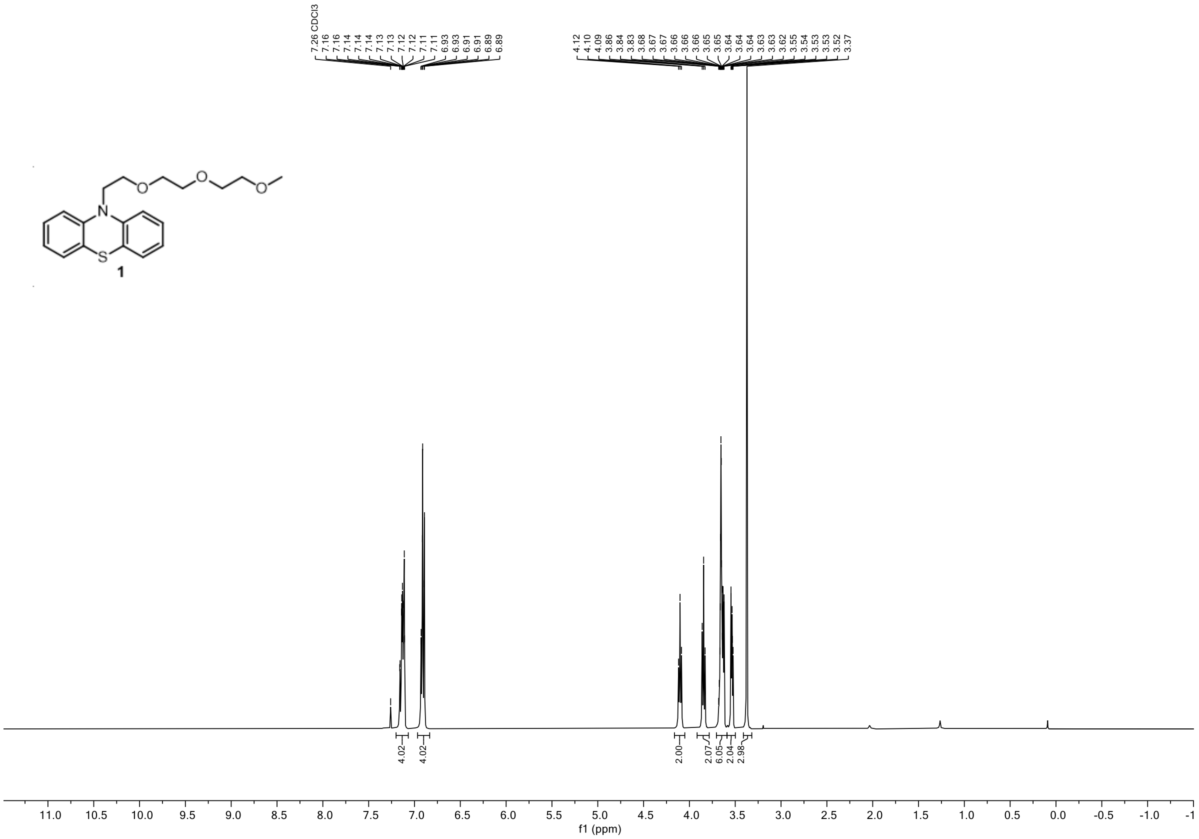
7.26 CDCl3
7.16
7.16
7.14
7.14
7.13
7.13
7.12
7.12
7.11
7.11
6.93
6.93
6.91
6.91
6.89
6.89
4.12
4.10
4.09
3.86
3.84
3.83
3.68
3.67
3.67
3.66
3.66
3.65
3.65
3.64
3.64
3.63
3.63
3.62
3.55
3.54
3.53
3.53
3.52
3.37
S
N
O
1
4.02
4.02
2.00
2.07
6.05
2.04
2.98
f1 (ppm)

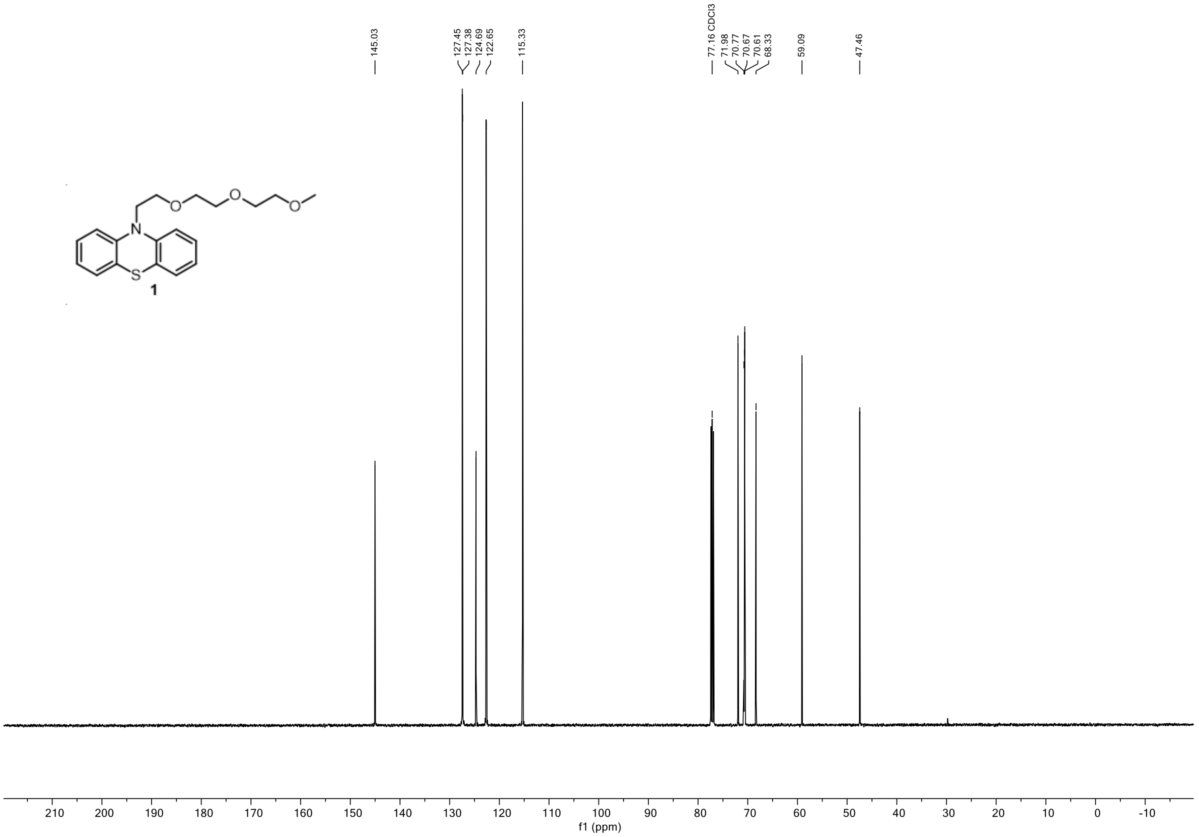
145.03
127.45
127.38
124.69
122.65
115.33
77.16 CDCl3
71.98
70.77
70.67
70.61
68.33
59.09
47.46
S
N
O
1
f1 (ppm)

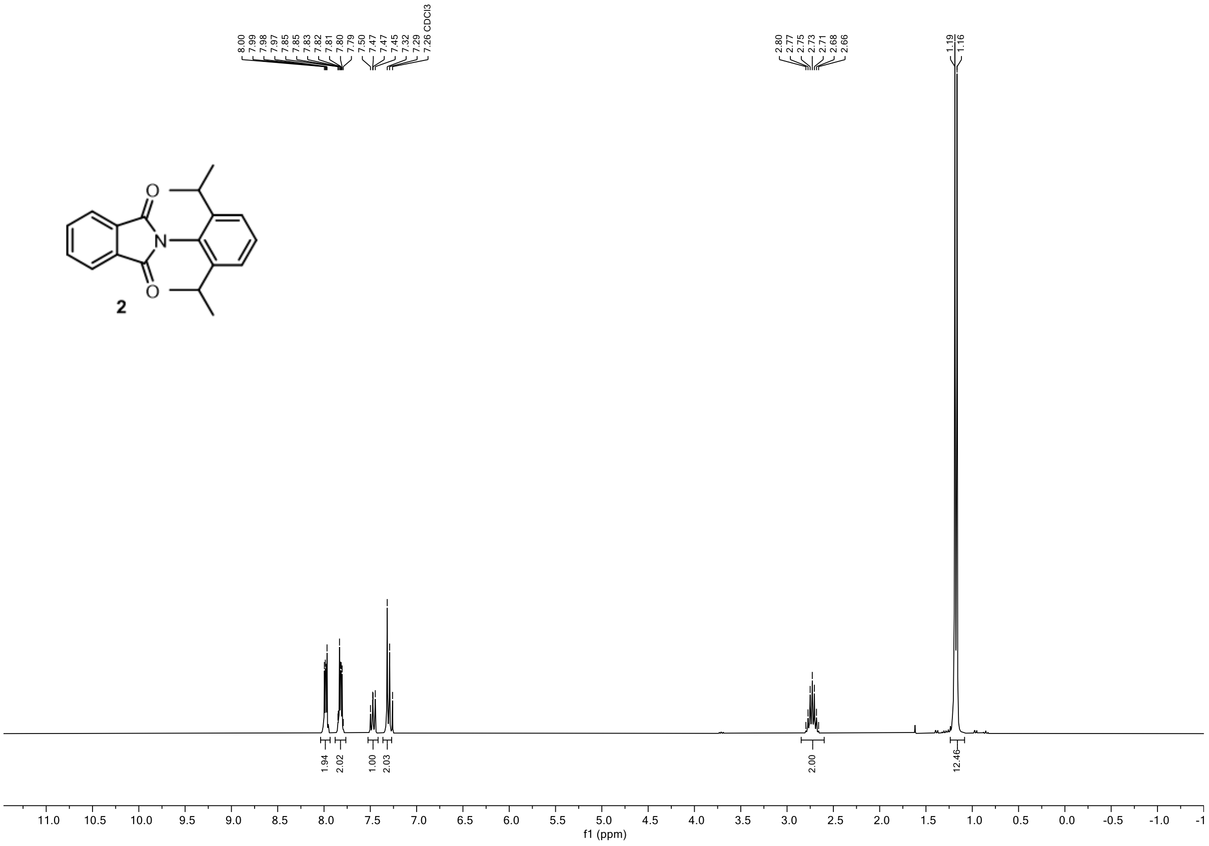
8.00 7.99 7.98 7.97 7.85 7.83 7.82 7.81 7.80 7.79 7.50 7.47 7.47 7.45 7.32 7.29 7.26 CDCl3
2.80 2.77 2.75 2.73 2.71 2.68 2.66
1.19 1.16
2
1.94 2.02 1.00 2.03 2.00 12.46
f1 (ppm)

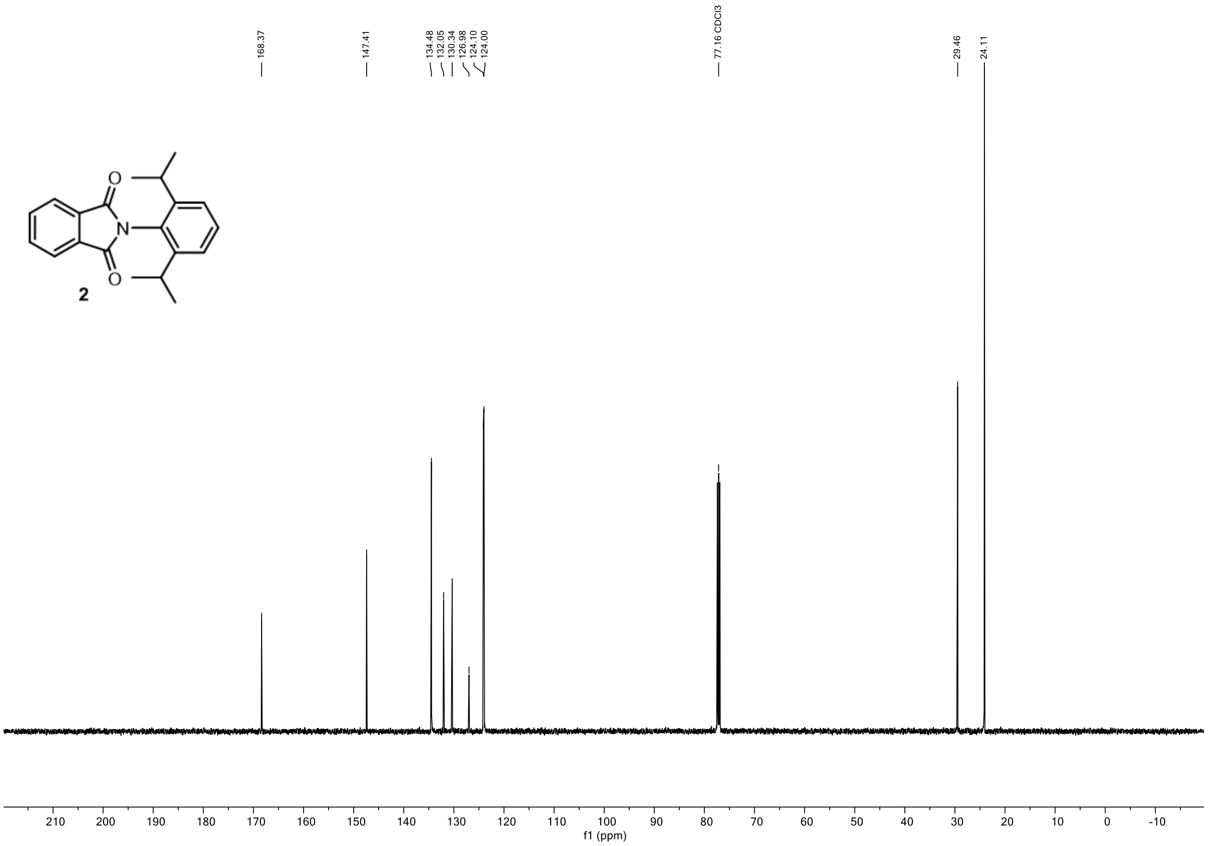
168.37
147.41
134.48 132.05 130.34 126.98 124.10 124.00
77.16 CDCl3
29.46
24.11
2
f1 (ppm)
